# Exploring the latest trends in chemistry, structure, coordination, and diverse applications of 1-acyl-3-substituted thioureas: a comprehensive review

**DOI:** 10.1039/d4ra02567a

**Published:** 2024-06-05

**Authors:** Sayyed Aqib Ullah, Aamer Saeed, Muhammad Azeem, Mian Bilal Haider, Mauricio F. Erben

**Affiliations:** a Department of Chemistry, Quaid-i-Azam University Islamabad 45320 Pakistan asaeed@qau.edu.pk; b Departamento de Química, CEQUINOR (UNLP, CONICET-CCT La Plata), Facultad de Ciencias Exactas, Universidad Nacional de La Plata Bv. 120 1465 La Plata 1900 Argentina

## Abstract

Acyl thioureas represent a privileged moiety with vast potential applicability across diverse fields, making them the subject of extensive research efforts. The inherent flexibility of thiourea facilitates the synthesis of a wide range of core structures with diverse functionalities and properties. The distinctive presence of hard and soft donor sites renders acyl thioureas inclined to act as versatile ligands, thereby engendering a diverse array of metal complexes incorporating acyl thiourea as a pivotal ligand. Extensive investigations into the synthesized acyl thioureas and their derivatives have culminated in the elucidation of their substantial potential across a spectrum of applications, spanning biological activities, materials chemistry, catalysis, and beyond. This literature review represents a continuation of our ongoing endeavor to compile comprehensive data on research endeavors concerning acyl thioureas over the past two years.

## Abstract 2

This literature update is a continuation of our efforts to compile comprehensive data on research endeavors concerning acyl thioureas over the past two years.

## Graphical abstract

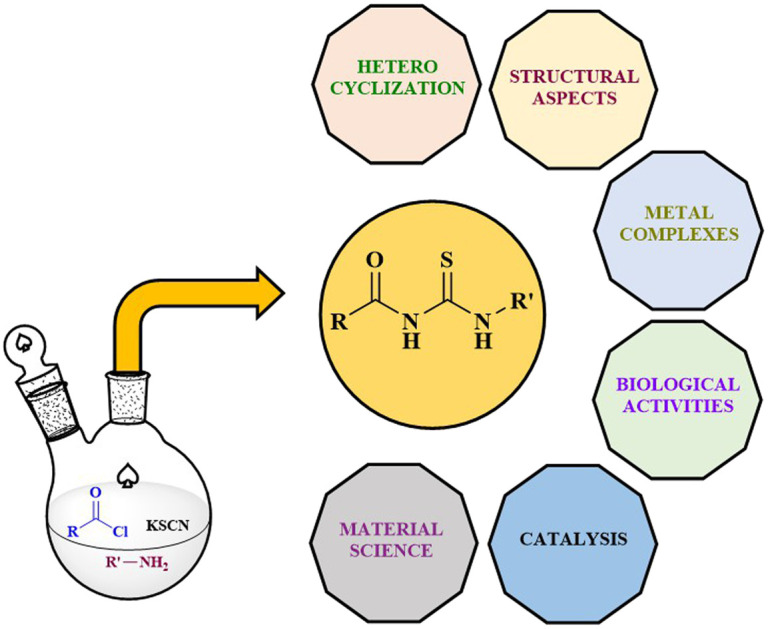

## 1. Introduction

**Fig. 1:**
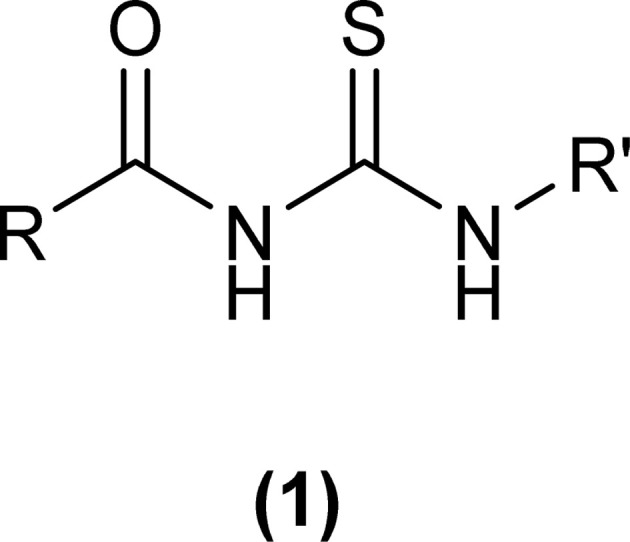
General structure of 1-acyl/aroyl thioureas.

1-(Acyl/aroyl)-3-(substituted)thioureas comprise a broad class of organosulfur compounds with the general formula [R^1^C(O)NHC(S)NR^2^R^3^], obtained by replacing the H atom of thiourea with an acyl/aroyl group. The general structure of 1-acyl/aroyl thioureas is shown in Fig. 1. Structural exploration demonstrates that acyl thiourea consists of a central hydrophilic part and lateral hydrophobic moieties. Acyl thioureas have garnered significant attention from the scientific community, becoming a focal point of research and admiration for many years.

Sulfur and nitrogen atoms within acyl thioureas offer numerous bonding opportunities, rendering them highly significant in coordinating with metal ions.^1^ These ligands show different coordination modes and have diverse applications in biological systems. Many remarkable studies have demonstrated the synthesis and exploration of a wide range of metal complexes featuring acyl thiourea derivatives, including compounds involving copper,^2^ cobalt,^3^ nickel,^4^ platinum,^5^ palladium,^6^ ruthenium^7^ and zinc,^3^ potentially leading to the development of new metal-based medications.

*N*-substituted-*N*-acyl thioureas appear as key building blocks for generating various heterocyclic products through cyclization,^8,9^ as well as serving as precursors for anion receptors,^10,11^ organocatalysts,^12–14^ corrosion inhibitors^15^ and non-ionic surfactants.^16^ Comprehensive research has documented the wide range of pharmacological benefits associated with acyl thiourea derivatives, including their potential as anticonvulsant,^17^ anticancer,^18–20^ antidiabetic,^21^ anti-inflammatory,^22,23^ anti-HIV,^24^ antimicrobial,^25,26^ urease inhibitory,^27^ herbicidal,^28^ and insecticidal agents.^29^

Several comprehensive reviews and compilation reports authored by Koch, Aly, and Saeed^14,30–36^ have systematically examined the literature pertaining to *N*-substituted-*N*-aroyl(acyl)thiourea chemistry. This review aims to provide a concise overview of the latest advancements in research on the mentioned structures. The focus will be on discussing progress in cyclization reactions, structure, applications, and biological activities since 2022, marking the timeframe of the most recent comprehensive review.

## 2. Synthesis

**Scheme 1:**
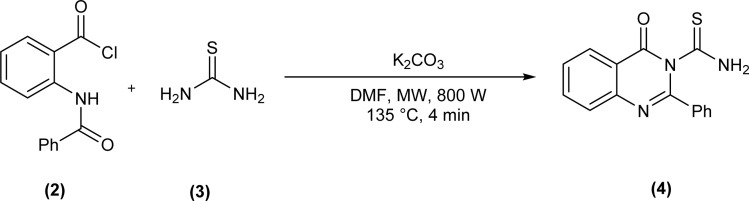
Microwave-assisted synthesis of 4-oxo-2-phenylquinazoline-3(4*H*)-carbothioamide.

Acyl/aroyl thioureas are a versatile class of organic compounds that can be synthesized by a variety of synthetic methods,^32^ but most widely used method for synthesis of acyl/aroyl thioureas is Douglas Dain's method,^37^ it involves the reaction of various amines with *in situ* generated acyl isothiocyanates in dry acetone or acetonitrile at a certain temperature.^38^ Following this methodology, Shankraiah *et al.* reported a new method for the synthesis of acyl thiourea in good yield from *in situ* generated acyl isothiocyanate and amino acid esters using Fe_2_O_3_ nanoparticles as heterogeneous catalysts.^39^ Sonoda *et al.* synthesized the aryloxy acetyl thiourea by reacting 2-phenoxyacetamide and phenyl isothiocyanate in dry DMF as solvent and sodium hydride as base.^40^ Microwave-assisted method for the synthesis of 4-oxo-2-phenylquinazoline-3(4*H*)-carbothioamide (4) was reported by Kumar Das *et al.* after reacting 2-benzamidobenzoyl chloride (2) and thiourea (3) in presence of potassium carbonate in dry DMF as solvent under microwave irradiation (800 W at 135 °C for 4 min) (Scheme 1).^41^

**Scheme 2:**
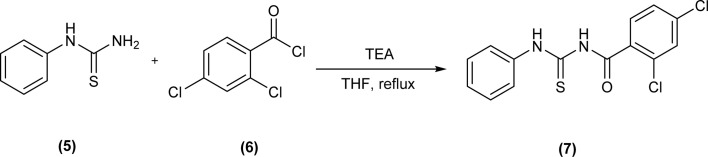
Synthesis of *N*-(2,4-dichloro)benzoyl-*N*′-phenylthiourea.

*N*-(2,4-Dichloro)benzoyl-*N*′-phenylthiourea (7) was synthesized by reacting *N*-phenylthiourea (5) and 2,4-dichlorobenzoyl chloride (6) in the presence of triethylamine and dry THF as solvent (Scheme 2).^42^

## 3. Heterocyclization reactions

**Scheme 3:**
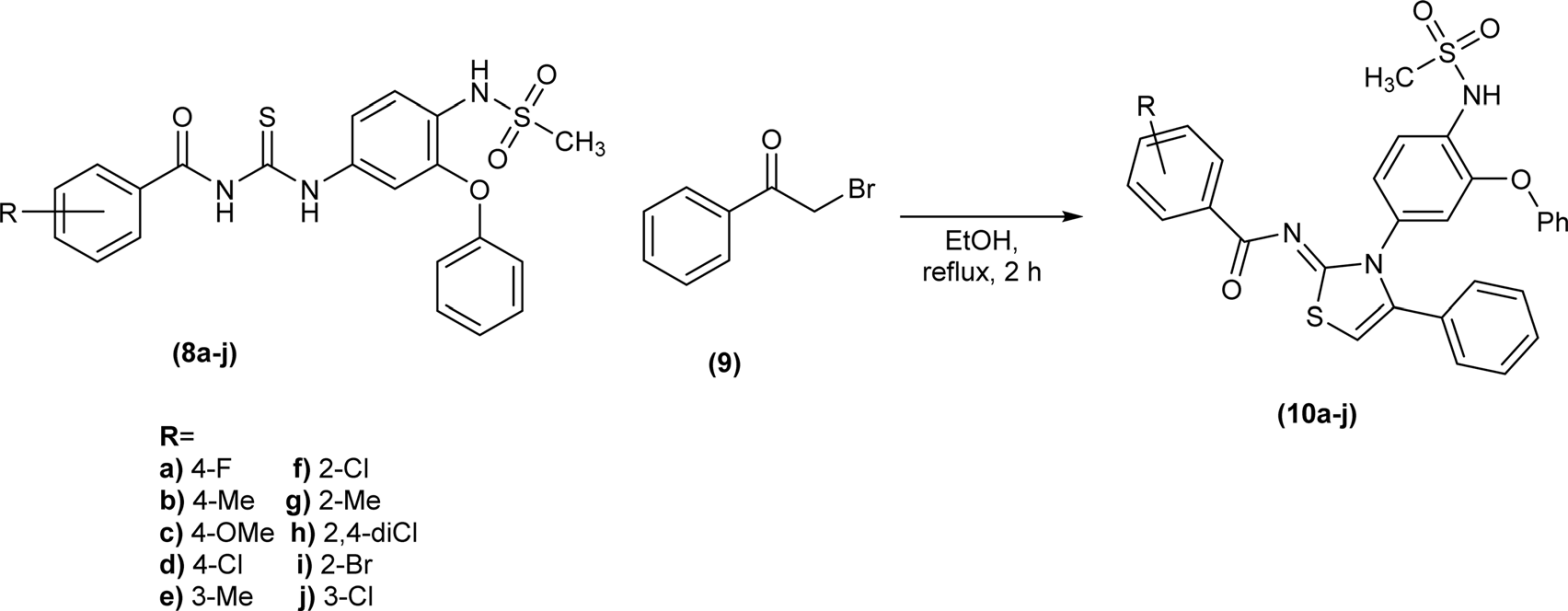
Synthesis of Nimesulide–iminothiazoline conjugates.

Shafique *et al.* synthesized a novel series of heterocyclic compounds, Nimesulide–iminothiazoline conjugates, first by reduction of the nitro group to form corresponding amine followed by its conversion to substituted acyl thioureas (8a–j). The reaction of these acyl thioureas (8a–j) with phenacyl bromide (9) in dry ethanol under reflux conditions furnished iminothiazoline derivatives (10a–j) in good yield (Scheme 3).^43^

**Scheme 4:**
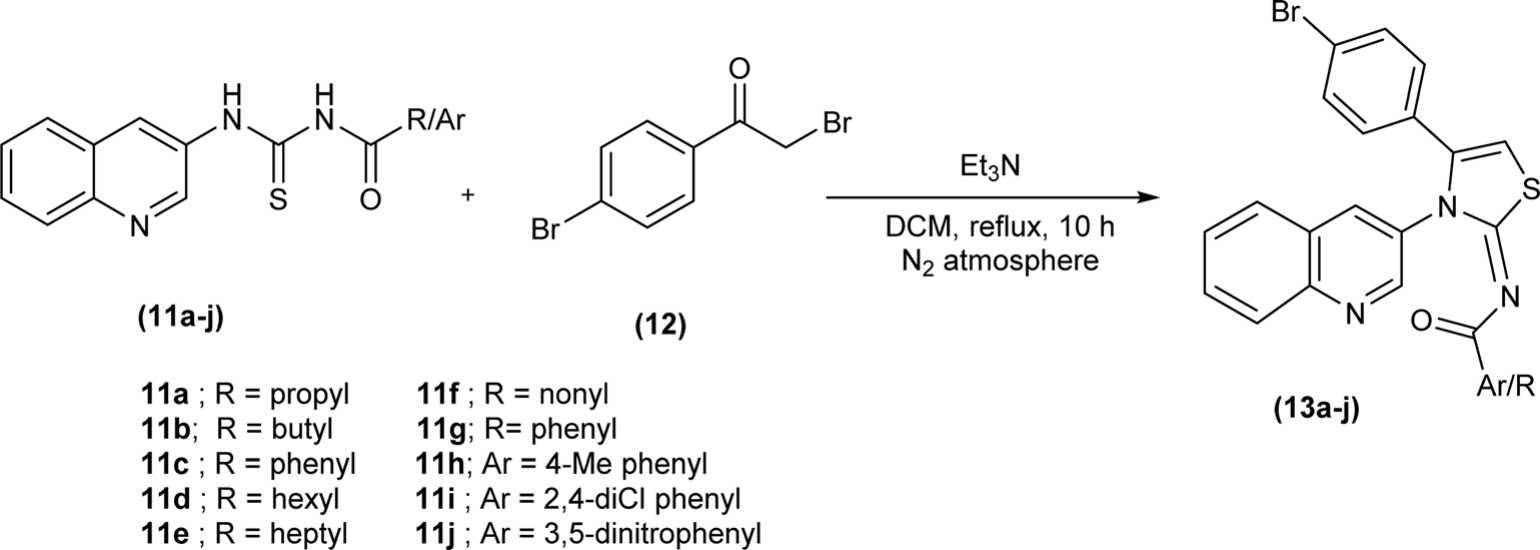
Synthesis of 4-bromo-*N*-(4-butyl-3-(quinolin-3-yl)thiazol-2(3*H*)-ylidene)benzamides.

Mustafa *et al.* synthesized the quinoline-based iminothiazoline, 4-bromo-*N*-(4-butyl-3-(quinolin-3-yl)thiazol-2(3*H*)-ylidene)benzamide by refluxing *p*-bromophenacyl bromide and acyl thiourea in DCM using trimethylamine as a base under nitrogen atmosphere for 10 hours.^44^ Similarly, a series of quinoline-based iminothiazoline derivatives (13a–j) were synthesized using the same procedure to obtain respective compounds in good yield (Scheme 4).^45^

**Fig. 2:**
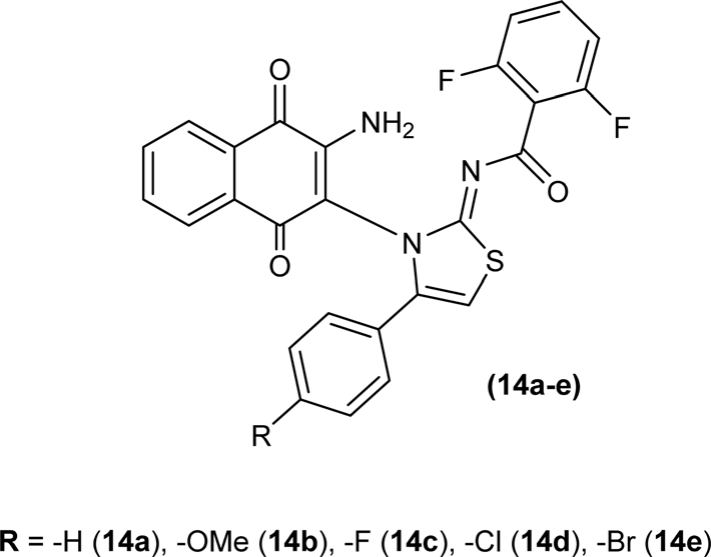
Naphthoquinone linked iminothiazoline.

Naphthoquinone-linked iminothiazoline derivatives were synthesized by Efeoglu *et al.* and the synthesized compounds were characterized by NMR and FT-IR spectroscopy, HRMS and stereochemistry of one of the compounds was determined using single crystal XRD analysis (Fig. 2). XRD study showed that the compound (14a) was crystalized in a triclinic crystal system adopting a *P*1̄ space group and one unit cell consisting of two iminothiazoline molecules.^46^

To synthesize further derivatives of naphthoquinone-linked iminothiazoline, the naphthoquinone-based acyl thioureas were reacted with differently substituted α-bromoketones. Compounds synthesized were then characterized using various spectroscopic techniques by Efeoglu *et al.*^47^

**Scheme 5:**
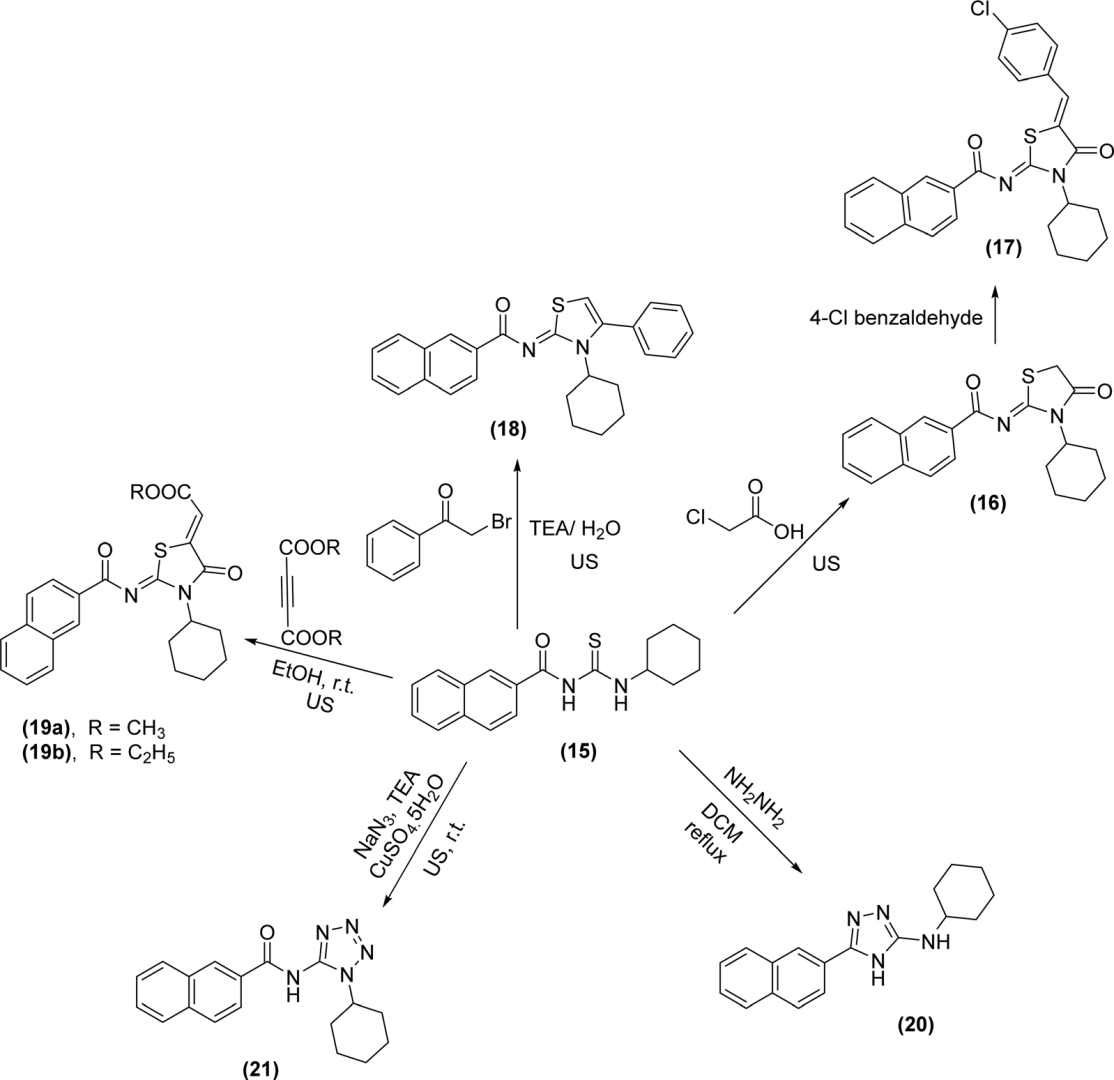
Heterocyclization of *N*-(cyclohexylcarbamothioyl)-2-naphthamide.

*N*-Naphthoyl acyl thiourea derivatives were synthesized by Arafa *et al.* and one of the synthesized derivatives *N*-(cyclohexylcarbamothioyl)-2-naphthamide (15) was subjected to different heterocyclization reactions. First, on reaction with chloroacetic acid in the presence of triethylamine produced thiazolidine analog (16), which *via* Knoevenagel condensation with 4-Cl-benzaldehyde furnished arylidene thiazolidine scaffold (17). Second, *via* cyclization with α-bromoacetophenone in the presence of trimethylamine under sonication give thiazole-2-imine (18). Third, thiazole derivatives (19a-b) of the acyl thiourea were synthesized by dropwise addition of dialkyl acetylenedicarboxylates to a stirred solution of acyl thiourea (15) in ethanol at room temperature. Cyclization of acyl thiourea derivative with hydrazine hydrate in DCM as solvent under reflux conditions furnished 1,2,4-triazole (20) and finally, tetrazole derivative (21) was synthesized by reacting thiourea and sodium azide under basic conditions using CuSO_4_·5H_2_O (50.0 mol%) as a catalyst (Scheme 5).^48^

**Scheme 6:**
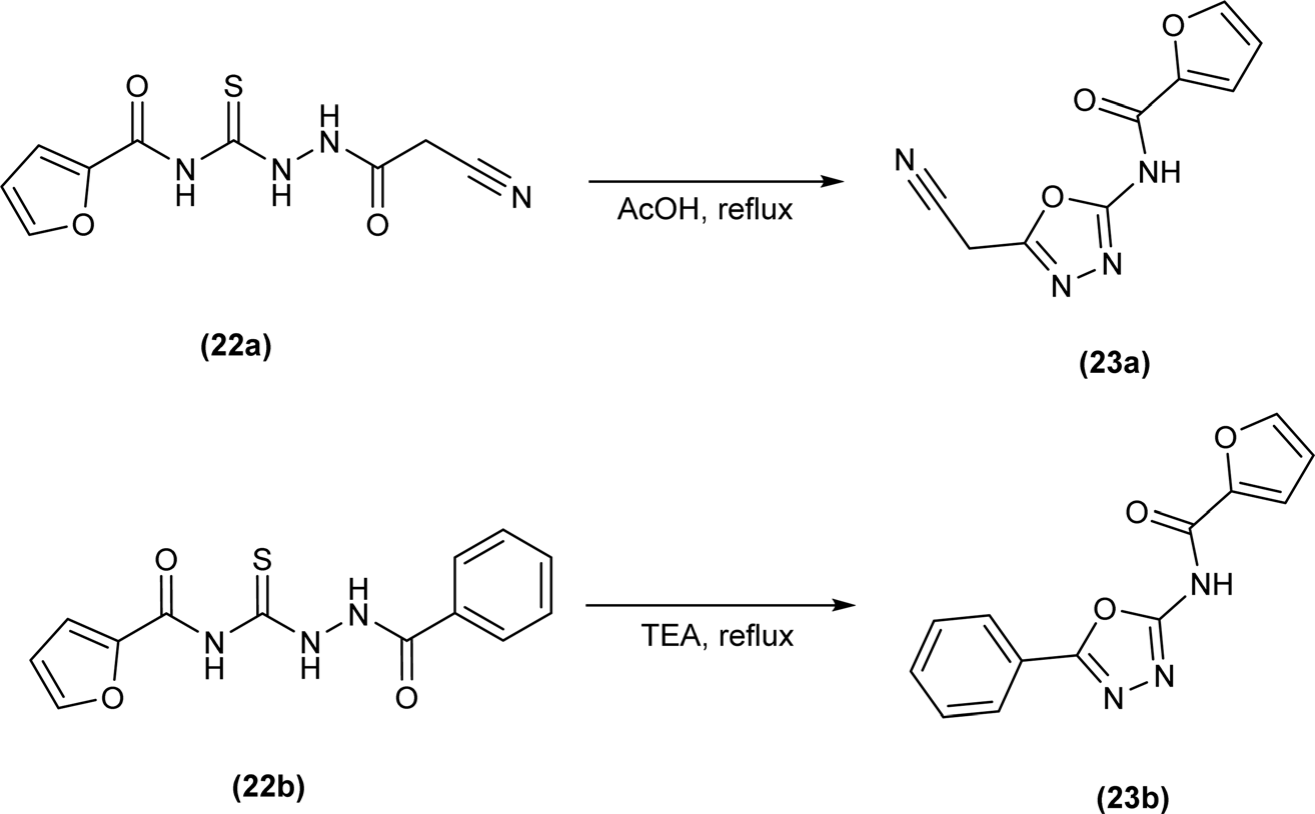
Synthesis of oxadiazole derivatives from thiosemicarbazides.

Novel oxadiazole derivatives (23a and 23b) were synthesized by Abdelhamid *et al.* from thiosemicarbazides (22a and 22b) *via* cyclization reaction by refluxing thiosemicarbazides in acetic acid and in trimethylamine, respectively to give oxadiazole (Scheme 6).^49^

**Scheme 7:**
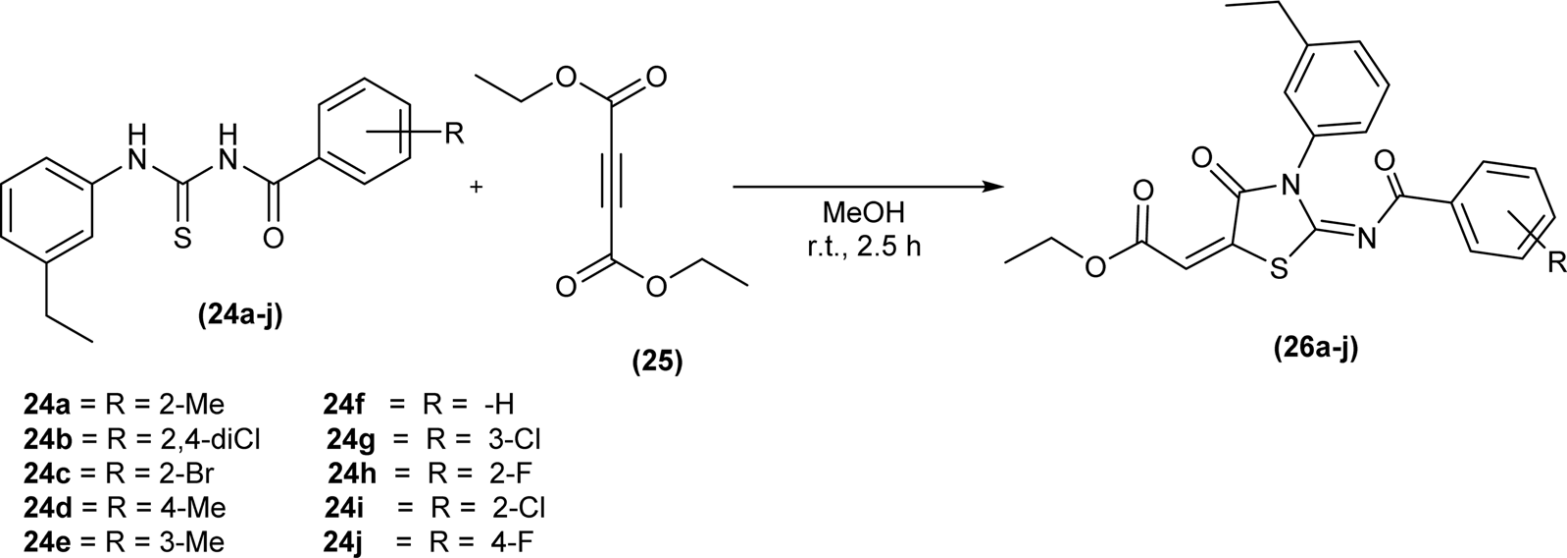
Synthesis of iminothiazolidinones.

Ahmed *et al.* synthesized a series of iminothiazolidinone derivatives (26a–j) by reacting equimolar amount of acyl thioureas (24a–j) and ethyl 4-ethoxypent-4-en-2-ynoate (25) in dry methanol as solvent at room temperature (Scheme 7).^50^ The same procedure was applied with success for the preparation of a novel series of amantadine thiazolidinones analogs from acyl thioureas and ethyl 4-ethoxypent-4-en-2-ynoate.^51^

**Scheme 8:**
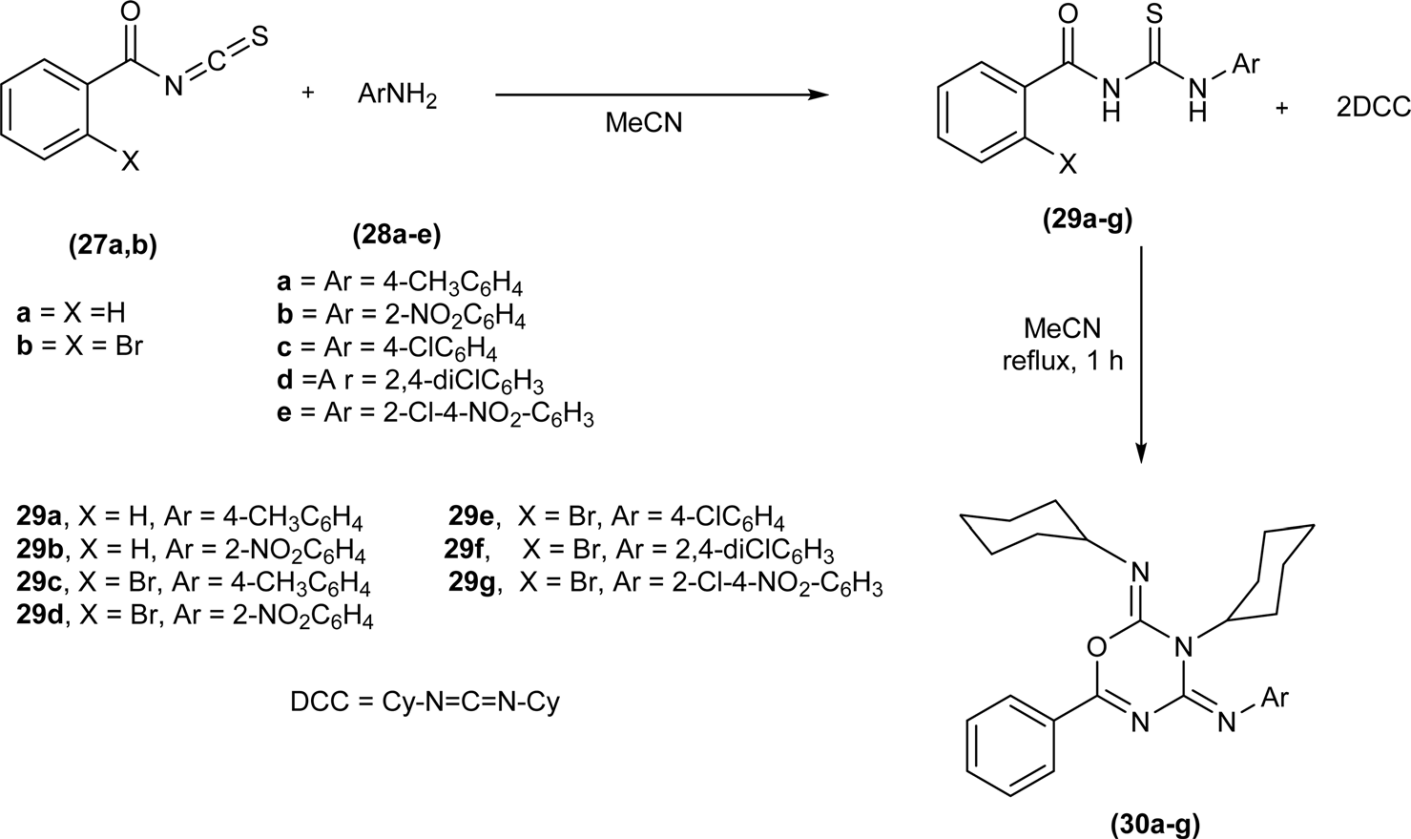
Synthesis of 2*H*-1,3,5-oxadiazine-2,4(3*H*)-diimines derivatives by dehydrosulfurization of acyl thiourea.

A new methodology was developed by Pokotylo *et al.* for the synthesis of 2*H*-1,3,5-oxadiazine-2,4(3*H*)-diimines derivatives (30a–g) by dehydrosulfurization of acyl thiourea (29a–g) with dicyclohexyl carbodiimide. Acyl thiourea derivatives (30a–g) were synthesized by reacting aryl amines (28a–e) with isothiocyanates (27a and 27b) in acetonitrile followed by reaction of isolated acyl thioureas (29a–g) with DCC in acetonitrile to afford the corresponding 2*H*-1,3,5-oxadiazine-2,4(3*H*)-diimines derivatives (30a–g) (Scheme 8).

The proposed mechanism involves the nucleophilic attack of the sulfur atom of acyl thiourea (29) on the sp hybridized carbon atom of DCC, followed by the transfer of hydrogen atoms from acyl thiourea to the DCC part accompanied by the breaking of weak C–S bond which leads to the formation of carbodiimide and *N*,*N*′-dicyclohexylthiourea. The resulting acyl carbodiimide undergoes [4 + 2] Diels–Alder cycloaddition reaction with another molecule of DCC afford 2*H*-1,3,5-oxadiazine-2,4(3*H*)-diimine derivatives (30a–g).^52^

**Scheme 9:**
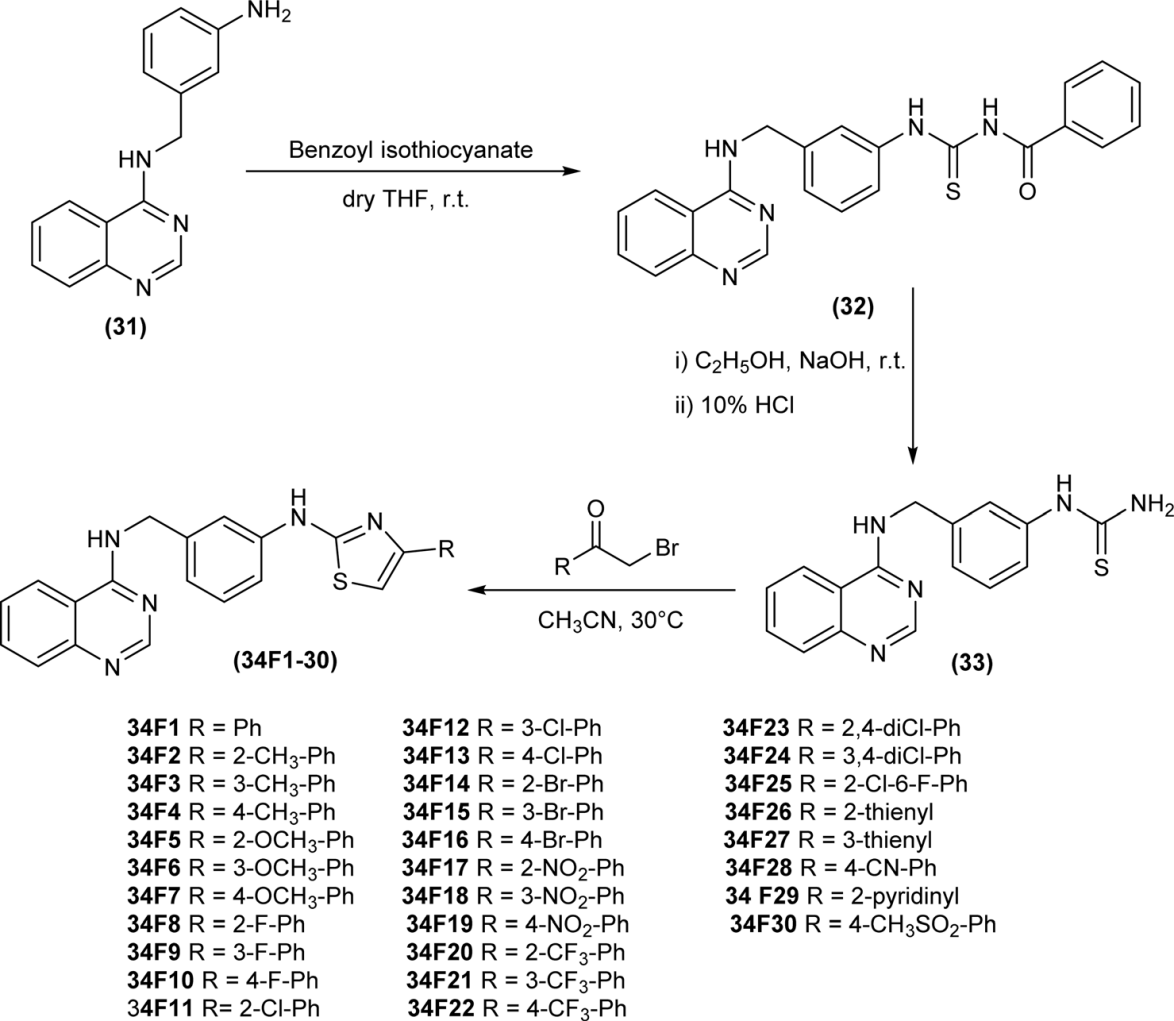
Synthesis of 2-aminothiazole derivatives.

Wan *et al.* synthesized a series of 2-aminothiazole derivatives (34F1-30), first of all reacting amine (31) with benzoyl isothiocyanate in dry THF furnished acyl thiourea derivative (32), followed by its hydrolysis using alkaline conditions to produce intermediate thiourea (33), which was further treated with different acetoaryl bromides in heated acetonitrile solution to afford desired 2-aminothiazole derivatives (34F1-30) (Scheme 9).^53^

**Scheme 10:**
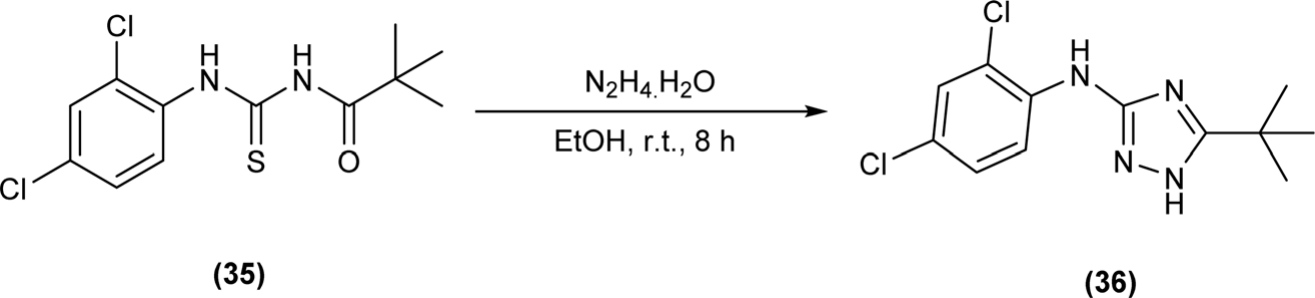
Synthesis of 5-(*tert*-butyl)-*N*-(2,4-dichlorophenyl)-1*H*-1,2,4-triazol-3-amine.

Cyclization of pivaloyl thiourea (35) to 5-(*tert*-butyl)-*N*-(2,4-dichlorophenyl)-1*H*-1,2,4-triazol-3-amine (36) was carried out by reacting pivaloyl thiourea (35) with hydrazine hydrate in ethanol at room temperature (Scheme 10).^54^

**Scheme 11:**
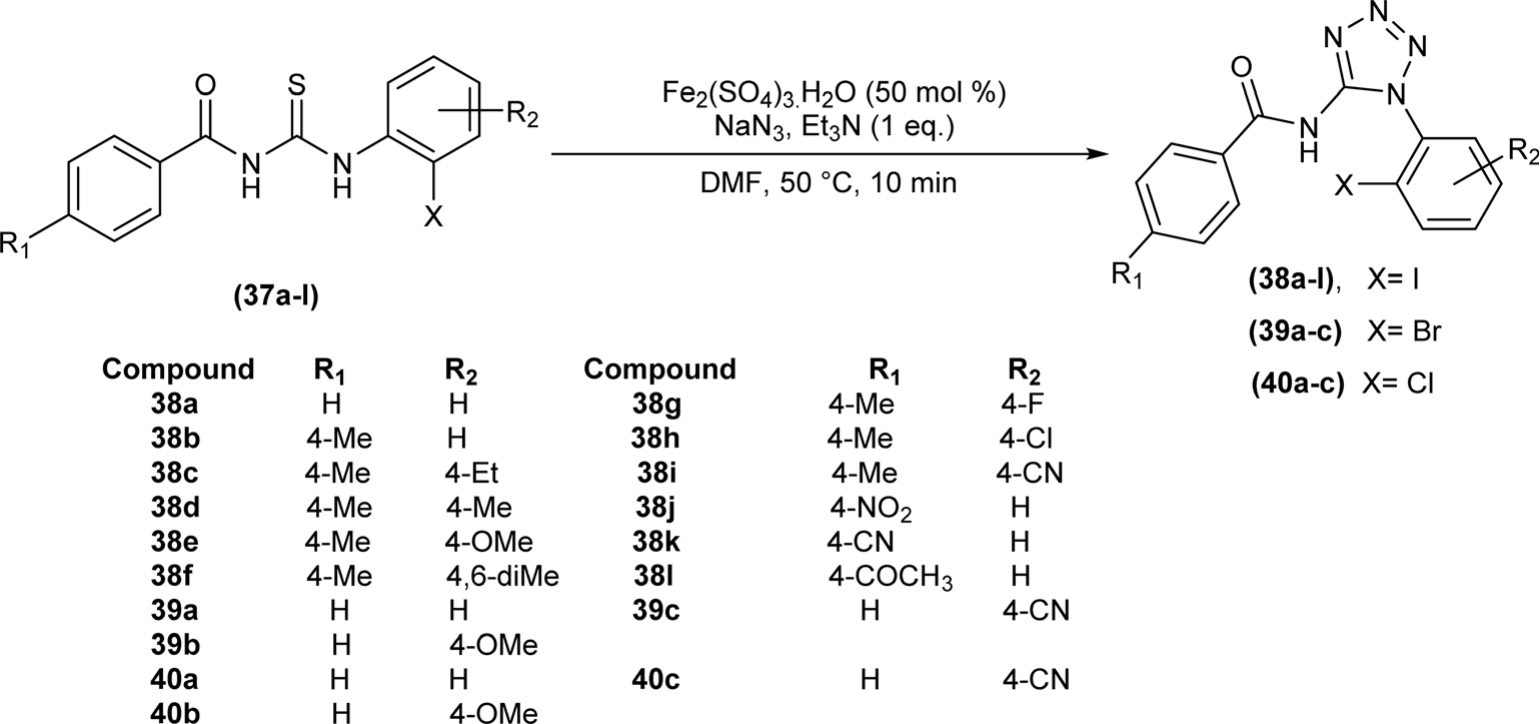
Synthesis of halo aroyltetrazoles from *N*-benzoyl-*N*′-halophenyl thiourea.

Halo aroyltetrazoles (38a–l, 39a–c, 40a–c) were synthesized by Gunturu *et al.* by addition of 50 mol% Fe_2_(SO_4_)_3_·H_2_O, NaN_3_, and Et_3_N to the solution of *N*-benzoyl-*N*′-halophenyl thiourea (37a–l) in DMF at room temperature for 10 min (Scheme 11).^55^

## 4. Molecular and crystal structure

Molecular structure and conformations of 1-(acyl/aroyl)-3-(mono-substituted) thioureas were studied extensively, and it is found that the enormous potential of conformational possibilities for acyl thioureas are one of the reasons behind their diverse biological activities.^56–58^ Extensive literature exploring the molecular structure and conformational properties of acyl thioureas, along with their potential applications, is readily available.^59^

**Scheme 12:**
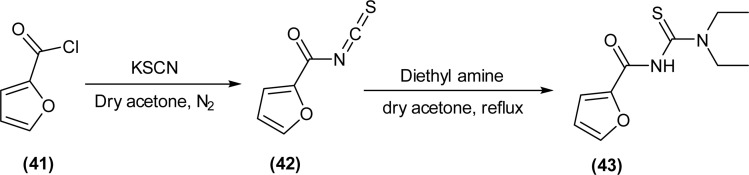
Synthesis of *N*-(diethylcarbamothioyl)furan-2-carboxamide.

**Fig. 3:**
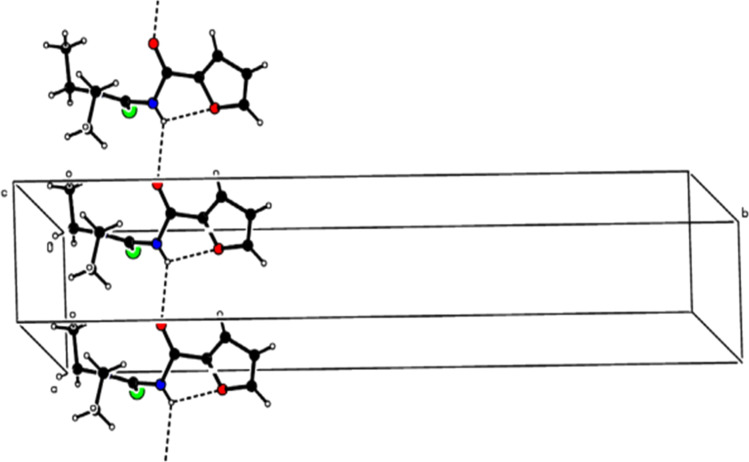
A partial packing diagram is viewed along the *c*-axis. N–H⋯O=C hydrogen bonds are shown as dashed lines. Sulfur is represented by green color, oxygen by red, and nitrogen by blue.

Novel acyl thiourea (43) having two diethyl groups and one heterocyclic furanyl group was synthesized (Scheme 12) by Saeed *et al.* From single crystal XRD analysis it was found that the centrosymmetric dimer of respective thiourea was due to the strong intermolecular hydrogen bonding between N–H and O=C and van der Waal's forces, this observation was also confirmed by Hirshfeld surface analysis. The large number of H⋯H, O⋯H and S⋯H intermolecular interactions were responsible for the crystal packing of molecules (Fig. 3). Also, these interactions were found as a key factor for the contribution towards the stability of the overall planar molecular structure based on the fact that the furan ring is almost planar to the carboxamide group.^60^

Sashankh *et al.* synthesized thirteen novel acyl thiourea derivatives *via* reaction of aliphatic/aromatic isothiocyanates and 4-(4-phenylpiperazin-1-yl)aniline in dry acetone. Data from single crystal XRD analysis of compounds (132a, 132b, and 132e) showed that all compounds crystallized in triclinic system adopting the *P*1̄ space group. It was found that intramolecular hydrogen bonding present between N–H and the carbonyl group of acyl thiourea molecule was responsible for molecular structure. The possibility of thiol tautomer was also proposed based on the short bond length of N(2)–C(1) (Fig. 59).^61^

*N*,*N*-Di-2,4-dimethoxybenzyl-*N*′-2-nitrobenzoylthiourea (88) was synthesized by Arslan and Binzet by reacting 4-nitrobenzoyl isothiocyanate with bis(2,4-dimethoxybenzyl)amine in dry acetone. XRD analysis posed that the title compound crystallized in a triclinic crystal system and adopted the *P*1̄ space group, carbonyl and thiocarbonyl tend to stay in opposite directions, and extensive delocalization was present at the acyl thiourea part of the molecule as indicated by the bond lengths, and crystal packing is due to intermolecular H-bonding among molecules (Fig. 29).^62^

*N*-Benzoylthiourea-pyrrolidine carboxylic acid derivatives (92aa–ae, 93aa–ae, 92ba–b and 93ba–bb) were synthesized by Poyraz *et al.* and characterized using IR, NMR, MS and Single Crystal X-ray diffraction, and DFT studies. Compounds (92ac and 93ab) crystallized in a monoclinic crystal system and adopted the *P*2_1_/*c* space group and *C*2/*c* space group, respectively with one molecule in the asymmetric unit. In compound (92ac) the intermolecular hydrogen bond formation between NH of indole and carbonyl C=O of maleimide led to the formation of chain structures on the *b*-axis of crystal and weak intermolecular aromatic C–H⋯O interaction between these chains was responsible for sheets formation. While weak intermolecular aliphatic C–H⋯O interaction is the reason behind the 3D crystal structure (Fig. 31).^63^

Alizada and Arslan synthesized a novel derivative of acyl thiourea 1-(4-chloro-benzoyl)-3-(2-trifluoromethyl-phenyl)thiourea (111) by reacting 4-chlorobenzoylchloride with KSCN followed by reaction with 2-(trifluoromethyl)aniline in dry acetone. The synthesized compound was characterized by spectroscopic techniques and single-crystal XRD. The XRD analysis found that the compound crystallized in a triclinic crystal system with a *P*1̄ space group. The amidic C–N bond length was shorter than the normal C–N single bond. Thus, it indicated strong delocalization of electron pair on nitrogen toward aromatic moiety in the molecule. Intramolecular hydrogen bond formation occurred between NH and carbonyl group oxygen and thus made the carbonyl group less reactive as compared to the thiocarbonyl group. Hirshfeld surface analysis showed that the crystal packing was dominated by intermolecular hydrogen bonds H⋯H (23.8%), H⋯S/S⋯H (14.5%), H⋯F/F⋯H (14.3%), C⋯H/H⋯C (14.2%), and Cl⋯F/F⋯Cl (9.0%) interactions (Fig. 45).^64^

Two acyl thiourea derivatives *N*-(allylcarbamothioyl)-2-chlorobenzamide (100b) and *N*-(allylcarbamothioyl)-2-methylbenzamide (125) were synthesized by reacting 2-chlorobenzoyl chloride and 2-methylbenzoyl chloride with KSCN and allylamine in dry acetone by Yeşilkaynak *et al.* and characterization of the synthesized compound was done by using spectroscopic and single crystal XRD techniques. Study of thermal behavior by TG/DTA of the synthesized compound depicted that both (100b) and (125) were thermally stable up to 136 and 132 °C, respectively. From XRD studies it was found that the compounds (100b) and (125) crystallized in a triclinic crystal system adopting the *P*1̄ space group. In both compounds bond lengths of S1–C2 and O1–C1 were in the range of double bond, and shorter bond lengths of C–N single bond than normal indicated delocalization in molecules. The presence of intramolecular hydrogen bond (N–H⋯O) was also confirmed from single crystal XRD analysis (Fig. 54).^65^

**Fig. 4:**
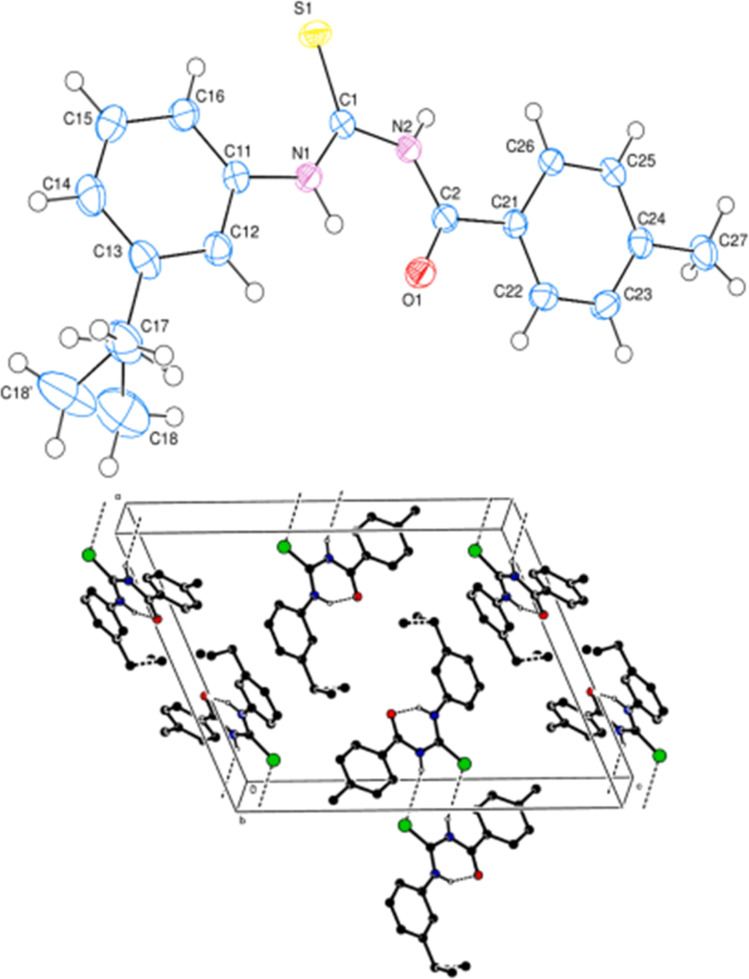
Molecular structure of compound ES-2 where thermal ellipsoid is drawn at the 50% probability level b: a partial packing diagram viewed along the *c*-axis. Hydrogen bonds are shown as dashed lines. Sulfur green, oxygen red, nitrogen blue.

Ahmed *et al.* synthesized alkyl-substituted acyl thiourea (141) from the reaction between 4-methylbenzoylchloride with KSCN followed by the addition of 3-ethylaniline in dry acetone. The compound was characterized by NMR and single-crystal XRD. From XRD analysis it was found that the compound crystallized in a monoclinic crystal system with *P*2_1_/*c* space group. Intermolecular N–H⋯S and intramolecular N–H⋯O hydrogen bond and H⋯H interaction were the reasons behind the crystal packing of the synthesized compound and these observations agreed with Hirshfeld surface analysis. Two planar aromatic rings A (C11–C12) and B (C21–C26) present at dihedral angle of A/B = 18.51(7)° to one another. Centrosymmetric dimer of molecules was formed due to N–H⋯S type intermolecular hydrogen bonding (Fig. 4).^66^

A series of four acyl thiourea (134a–d) were synthesized by Emen *et al.* and were characterized by spectroscopic techniques and single crystal XRD. Acyl thioureas (134a and 134d) crystallized in a monoclinic crystal system with a *P*2_1_/*c* space group and an orthorhombic crystal system with *Pbca* space group, respectively. XRD analysis further verified the presence of intramolecular hydrogen bonds (N–H⋯O) in acyl thiourea molecules. Bond length showed the conjugation in molecules extending from NH to carbonyl and thiocarbonyl groups (Fig. 61).^67^

**Fig. 5:**
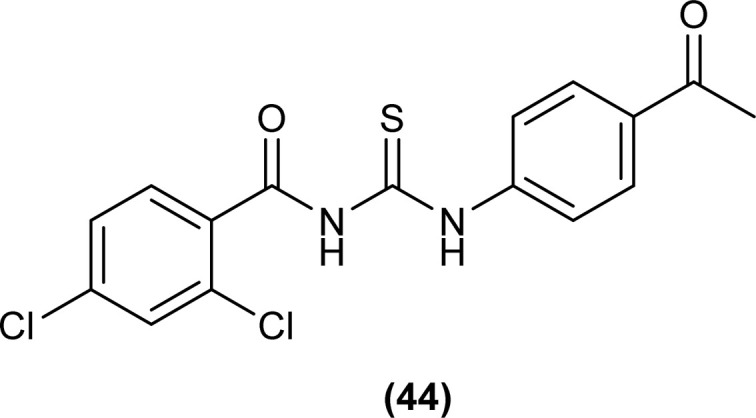
Structure of *N*-((4-acetylphenyl)carbamothioyl)-2,4-dichlorobenzamide.

Novel acyl thiourea derivative (44) was synthesized and characterized by Khalid *et al.*, single Crystal XRD analysis showed that the compound crystallized in a monoclinic crystal system with a *P*2_1_/*n* space group. Two planar aromatic rings of the compound were present at a dihedral angle of 33.32(6)° to one another. The three-dimensional structure of the compound was due to the presence of C–H⋯π and π⋯π interactions having inter-centroid distance 3.694 (1) Å. Hirshfeld surface (HS) investigation of the compound revealed that crystal packing of molecules was due to H⋯C/C⋯H (20.9%), H⋯H (20.5%), H⋯Cl/Cl⋯H (19.4%), H⋯O/O⋯H (13.8%) and H⋯S/S⋯H (8.9%) interactions, hydrogen bonding and van der Waals interaction played important role in crystal packing (Fig. 5).^68^

**Fig. 6:**
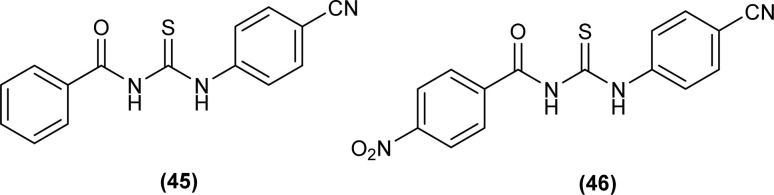
Structures of *N*-benzoyl-*N*′-(40-cyanophenyl)thiourea and *N*-(4-nitrobenzoyl)-*N*′-(40-cyanophenyl)thiourea.

Two novel acyl thiourea *N*-benzoyl-*N*′-(4-cyanophenyl)thiourea (45) and *N*-(4-nitrobenzoyl)-*N*′-(4-cyanophenyl)thiourea (46) were synthesized and their structure was determined by single crystal XRD analysis, it was found that the compound (45) crystallized in triclinic crystal system adopting *P*1̄ space group. Crystal packing of the molecules crystal was stabilized by intramolecular C=O⋯H–N hydrogen bond and intermolecular C=S⋯H–N and C=S⋯H–C hydrogen interactions (Fig. 6).^69^

**Fig. 7:**
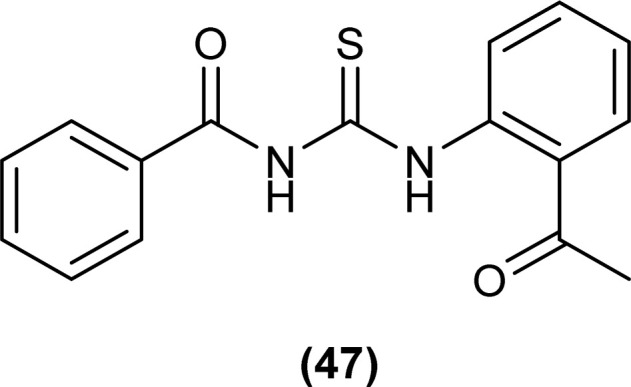
Structure of *N*-((2-acetylphenyl)carbamothioyl)benzamide.

Novel acyl thiourea *N*-((2-acetylphenyl)carbamothioyl)benzamide (47) crystallized in a triclinic crystal system and adopted *P*1̄ space group. The short bond length of the C–N bond indicated the presence of strong delocalization in the acyl thiourea moiety of the molecule. Crystal packing of the molecule was attributed to the intermolecular (C–H⋯O) and three intramolecular (N–H/O, C–H/S) H-bonds (Fig. 7).^70^

**Fig. 8:**
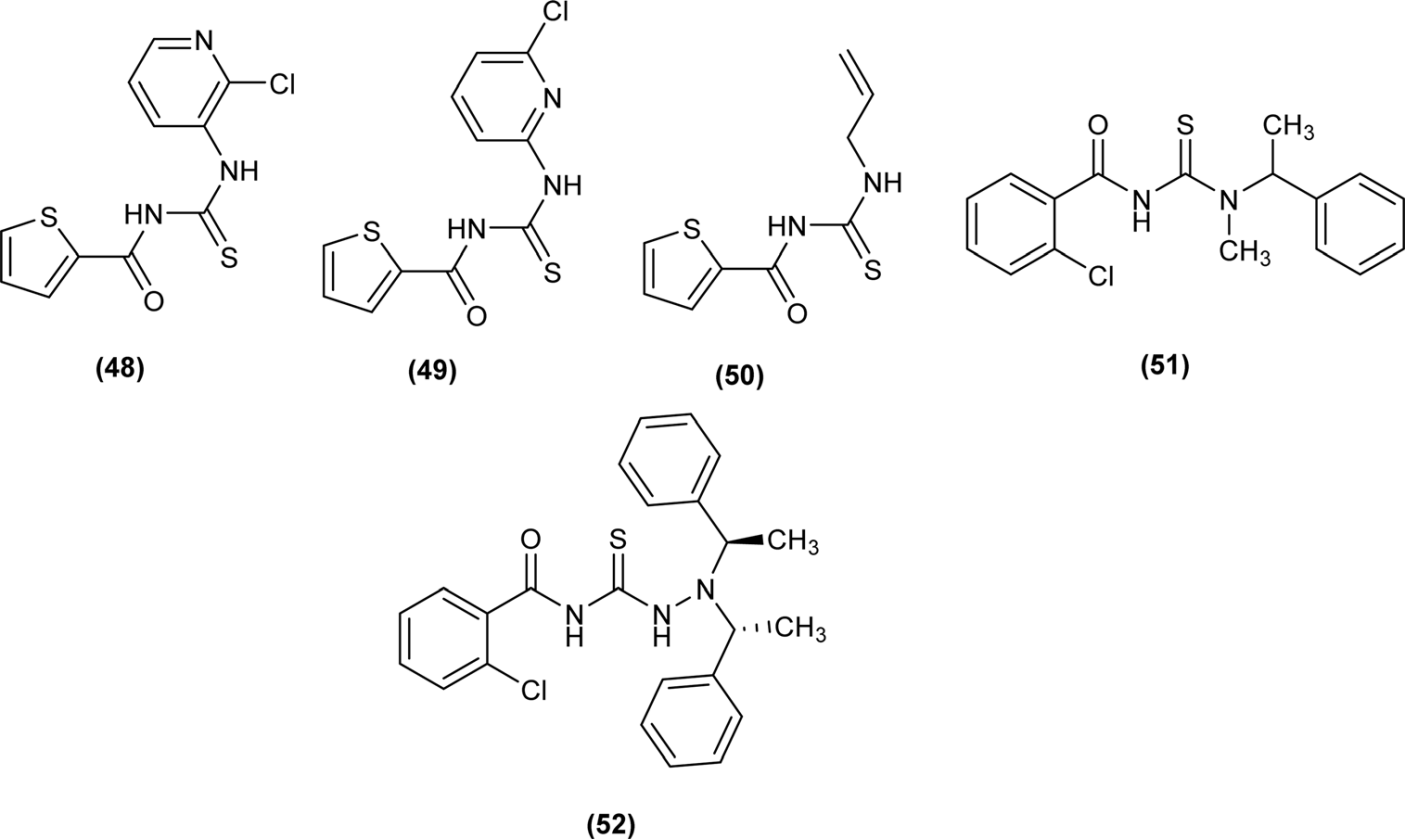
Thiophene linked acyl thioureas.

A series of five novel acyl thiourea derivatives (48–52) were synthesized and a single Crystal XRD analysis study showed that the compound (50) crystallized in a monoclinic crystal system with *P*2_1_/*n* space group. Only the intermolecular hydrogen bond (O⋯H) between H of NH and O of carbonyl was responsible for the compact crystal packing (Fig. 8).^71^

**Fig. 9:**
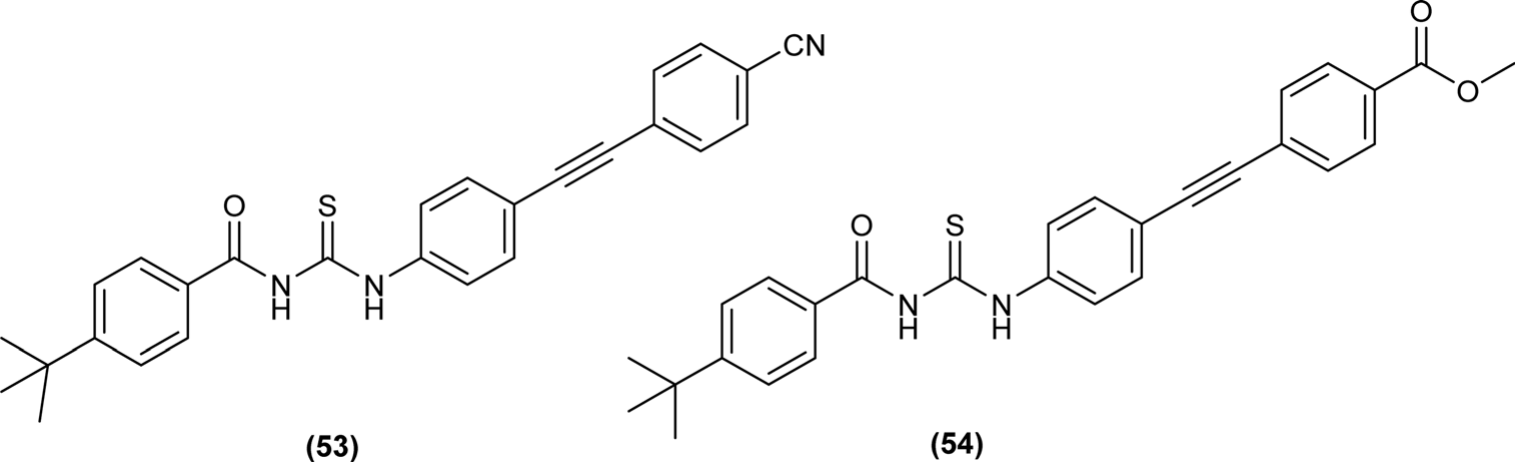
Structures of ethynylated-acyl thioureas.

Two novel ethynylated-acyl thiourea were synthesized (53 and 54) and single crystal XRD analysis of compound (54) exhibits S conformation in center of acyl thiourea moiety (–C(O)NHC(S)NH) and crystallized in triclinic crystal system with *P*1̄ space group. According to Hirshfeld surface analysis, crystal packing of the molecule was due to the presence of intermolecular N–H⋯O=C and N–H⋯S=C hydrogen bonding in the molecules (Fig. 9).^72^

Single crystal XRD analysis of the 2*H*-1,3,5-oxadiazine-2,4(3*H*)-diimine derivative (30d) showed that the compound crystallized in a monoclinic crystal system and adopted the *P*2_1_/c space group. It was found that the oxadiazine ring in the molecule was flat with an accuracy of 0.03 Å (Scheme 8).^52^

5-(*Tert*-butyl)-*N*-(2,4-dichlorophenyl)-1*H*-1,2,4-triazol-3-amine (36) crystallized in orthorhombic crystal system with *Pbca* space group. Crystal structure stability of the molecule was due to the presence of intermolecular N–H⋯N hydrogen bonds and π⋯π stacking interaction between B rings (Scheme 10).^54^

XRD analysis of novel compound (*Z*)-4-bromo-*N*-(4-butyl-3-(quinolin-3-yl)thiazol-2(3*H*)-ylidene)benzamide (13b) showed that the compound crystallized in triclinic crystal system adopting *P*1̄ space group. Intermolecular C–H⋯O and C–H⋯N hydrogen bonds, π–π stacking and C–H⋯π interactions were responsible for the packing of molecules in the crystal structure (Scheme 4).^44^

**Fig. 10:**
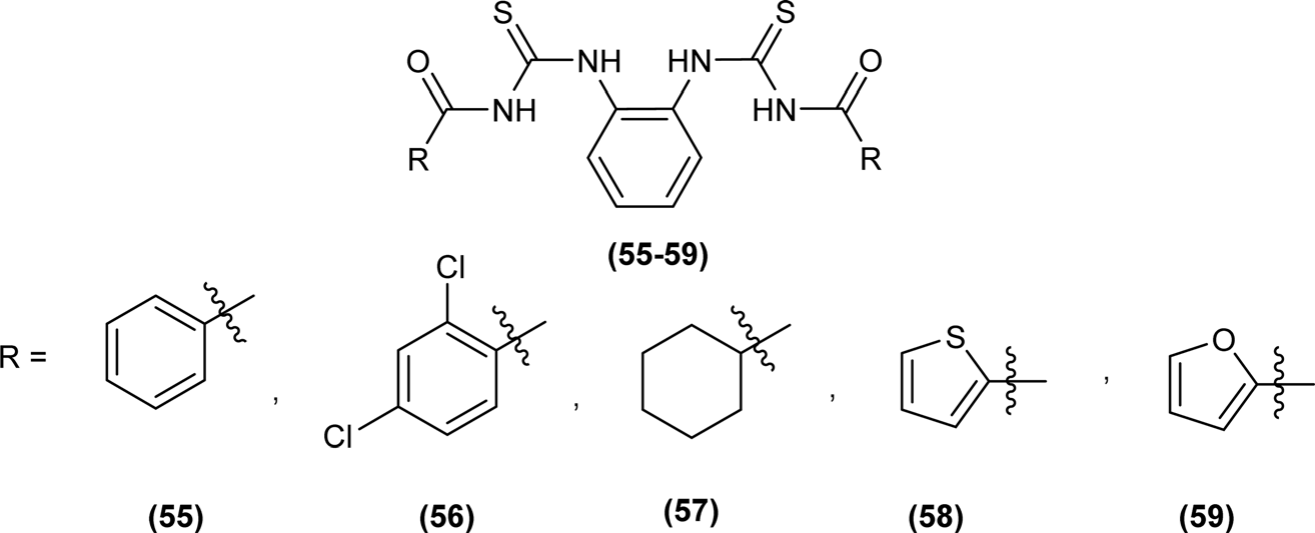
Structures of bis(acyl thiourea) derivatives.

XRD analysis of the bis(acyl thiourea) (57, 58 and 59) showed that the compound (57) and (58) crystallized in a monoclinic crystal system and adopted *C*2/*c*, while compound (59) crystallized in a triclinic crystal system with *P*1̄ space group (Fig. 10). The structure of compounds (57 and 58) was stabilized by intramolecular N–H⋯O interactions, crystal packing of the compound was due to strong N–H⋯S interactions leading to the generation of a centrosymmetric dimer *R*_2_ (8) ring, while that of compound 58 crystal packing stability was due to intermolecular hydrogen bonding of the type N–H⋯O and C–H⋯O. Similarly, several inter and intramolecular interactions were also observed. Intermolecular hydrogen bonding N–H⋯O, C–H⋯O, and C–H⋯S was the reason behind the crystal stability of compound 59.^73^

## 5. Metal complexes

The carbonyl, thiocarbonyl and nitrogen atoms of acyl thiourea provide this moiety efficient ligating ability, therefore, various metal complexes with potential applications are a field of interest for researchers.^74^ In this section, the synthesis and structural aspects of such metal complexes are presented.

*N*-((5-Bromopyridin-2-yl)carbamothioyl)-2-chlorobenzamide HL^1^ and *N*-((5-bromopyridin-2-yl)carbamothioyl)furan-2-carboxamide HL^2^ and their metal complexes (123 and 124) with Co, Ni, and Cu were synthesized by Yeşilkaynak *et al.* by combining individual ethanolic solutions of metal and ligand in the presence of triethyl amine. Single crystal XRD analysis of the ligands showed that HL^1^ and HL^2^ ligands crystallized in monoclinic and orthorhombic systems with space group *P*2_1_/*c* and *P*2_1_/*n* respectively. Both were found to be thermally stable up to 119 °C and 134 °C respectively. The tetrahedral geometry of Co(ii) and Ni(ii) complexes and square planar geometry of Cu(ii) complexes was confirmed by magnetic susceptibility studies (Fig. 53).^75^

**Fig. 11:**
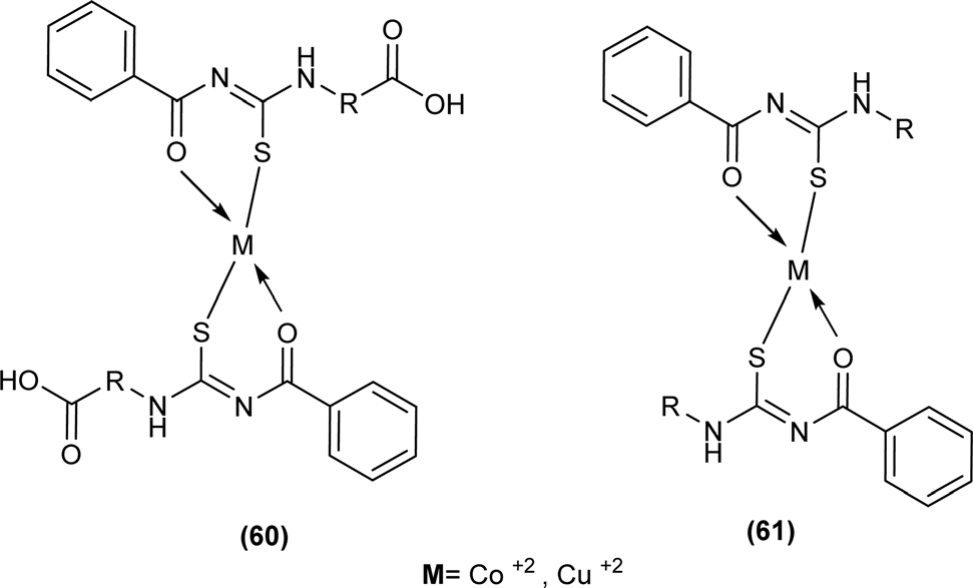
General structure of Cu(ii) and Co(ii) complexes of benzoyl thioureas.

Shah *et al.* synthesized a series of benzoyl thiourea derivatives and their metal complexes (60 and 61) with Cu(ii) and Co(ii) metals by addition of a methanolic solution of metal chloride to methanolic solution of acyl thiourea with adjustment of pH of thiourea solution by addition of sodium hydroxide. The synthesized compounds were assessed for their free radical scavenging and antibacterial activity (Fig. 11).^76^

Zhao *et al.* synthesized MOF {[Pr(H_2_L)_2_(NO_3_)(H_2_O)_2_]·2H_2_O·MeOH}_*n*_ by reacting acyl thiourea ligand (3-(naphthalene-2-carbonyl)-thioureido)acetic acid (H_2_L) with Pr(NO_3_)_3_·6H_2_O in methanol followed by addition of water. Synthesized compound crystallized in the monoclinic system adopting *C*2/*c* space group. The metal–organic framework consisted one Pr^3+^ ion, two H_2_L^−^ and two coordinated H_2_O units, two free water units, nitrate and one methanol molecule. Carboxylate groups acted as bidentate chelating ligands and bidentate bridging ligands, thus bridging the metal ions. Intramolecular hydrogen bonding was observed between –NH and O of carbonyl in the ligand. In addition, extensive intermolecular H-bonding and π–π stacking between aromatic parts was also observed. This extended hydrogen bond network was also responsible for the proton transport.^77^

**Fig. 12:**
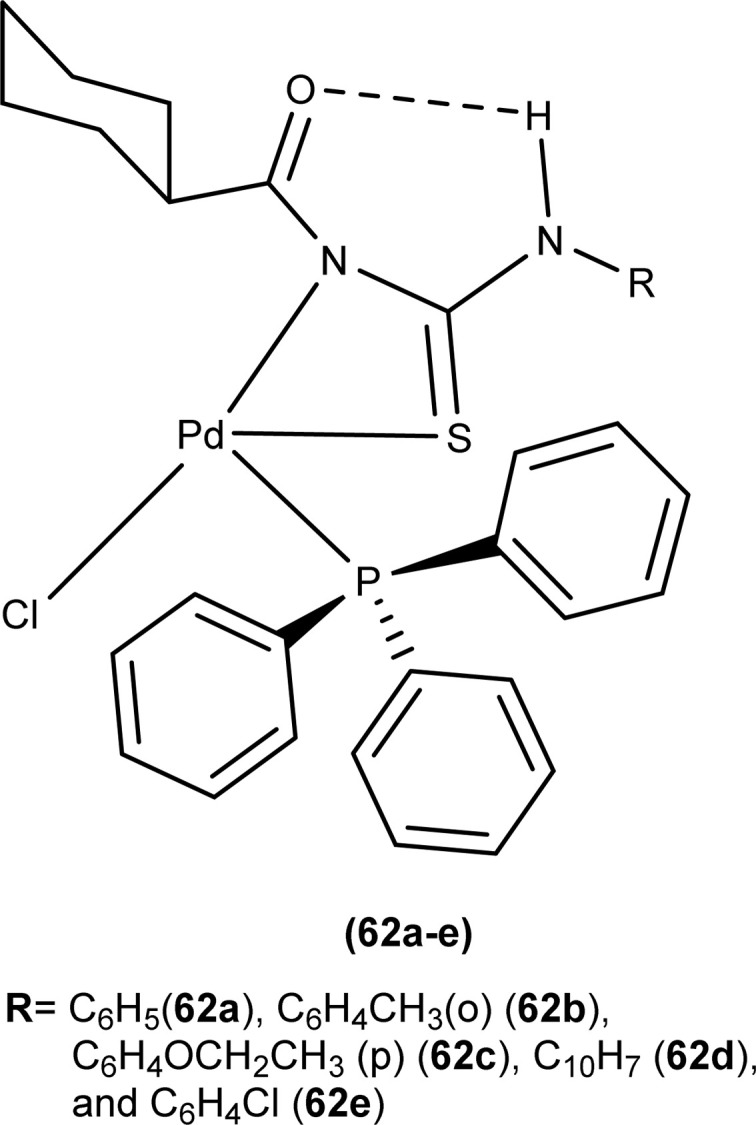
Palladium complexes of acyl thioureas.

Dorairaj *et al.* synthesized metal complexes of acyl thiourea (62a–e) with [PdCl_2_(PPh_3_)_2_] in acetonitrile and DCM as a solvent in the presence of a few drops of triethyl amine (Fig. 12). From single crystal XRD analysis, it was found that these metal complexes crystallized in the triclinic system and adopted the *P*1̄ space group. Intramolecular hydrogen bonding between carbonyl and NH of thioamide was observed. The coordination sphere adopted a slightly distorted square planar geometry around the metal ion and the central metal ion was surrounded by bidentate acyl thiourea (through S and N), one Cl, and one PPh_3_ ligands.^78^

**Fig. 13:**
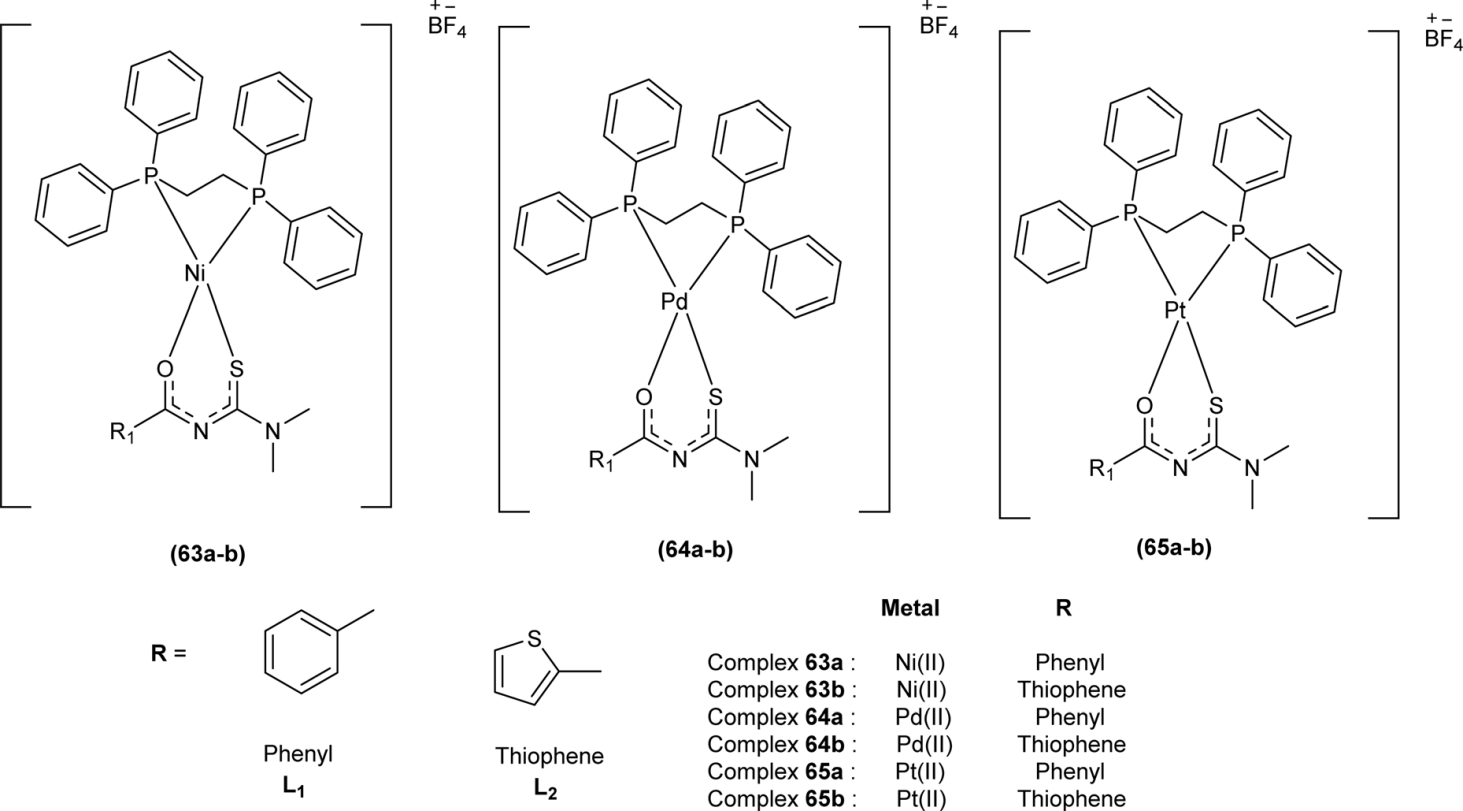
Acyl thiourea-based metal complexes of Ni, Pt and Pd.

De Oliveira *et al.* synthesized a series of acyl thiourea-based metal complexes with Ni, Pt and Pd by dissolving acyl thiourea ligands (L1 and L2) in methanol followed by the addition of respective precursor ([NiCl_2_(dppe)], [PdCl_2_(dppe)] or [PtCl_2_(dppe)]) and NaBF_4_ salt (Fig. 13). Single crystal XRD analysis of complexes showed that the complexes adopted distorted square planar geometry in which the respective metal ions Ni^II^ (63a-b), Pd^II^ (64a-b) and Pt^II^ (65a-b) were surrounded by one dppe molecule through two phosphorus atoms and one bidentate acyl thiourea ligand through S and O atoms in the monoanionic form. Nickel complexes crystallized in a non-centrosymmetric system adopting the *P*2_1_ space group while other complexes crystallized in a centrosymmetric system having a *P*1̄ space group. Delocalization in complexes compared to ligands was proposed based on the increase in bond lengths of thiocarbonyl and carbonyl and the decrease in the bond length of C–N bond. Another characteristic feature of complex (63a) was the different intramolecular interactions. In one complex intramolecular π stacking sandwich interaction between two phenyl groups was observed while the other lacked this type of interaction.^79^

**Fig. 14:**
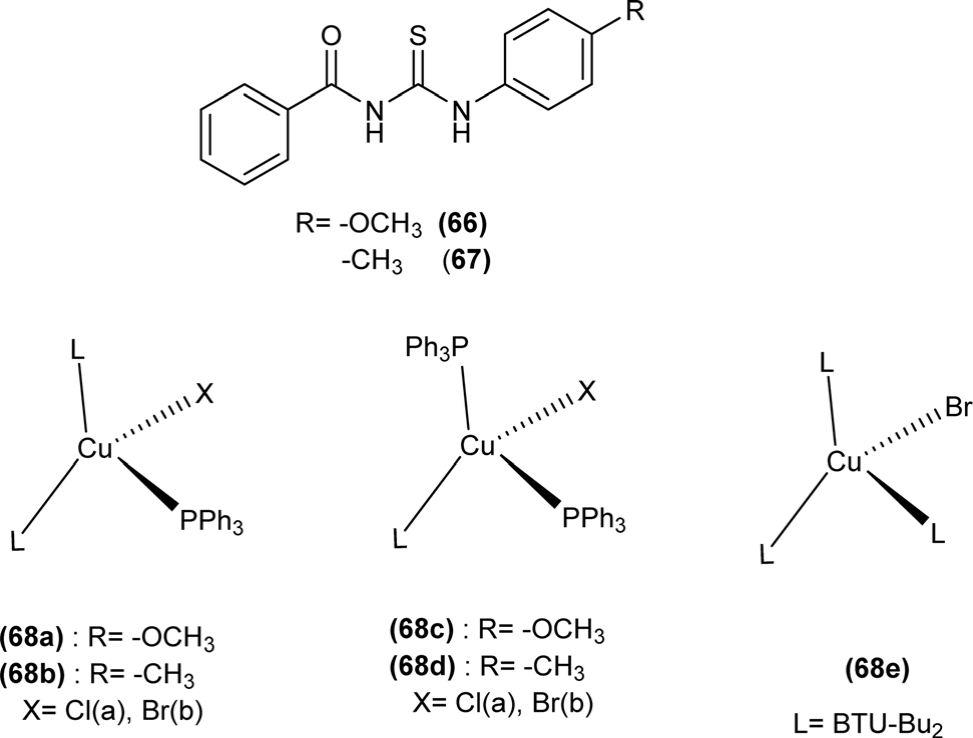
Copper(i) complexes of thioureas.

Tudor *et al.* synthesized a series of Cu(i) complexes with acyl thiourea and phosphine ligands by adding *N*-benzoyl thiourea ligands (66 or 67) to the solution of precursors [CuCl(PPh_3_)_4_] or [CuBr(PPh_3_)_3_] in toluene (Fig. 14). Single crystal XRD analysis of the complexes showed that the compounds crystallized in a trigonal crystal system adopting the *P*1̄ space group based on the observation that Cu(i) ion appeared to be surrounded by two PPh_3_ groups, one acyl thiourea neutral ligand and one chloride anion. The distorted tetrahedral geometry was due to the slight variations in bond angles between ligands, the largest contributor being the steric interactions between two bulkier PPh_3_ groups. Efficient molecular packing was attributed to CH–π and H-bond interactions between N1–H1 and N2–H2 as H donors and C11 and O1 as acceptors respectively.^80^

**Fig. 15:**
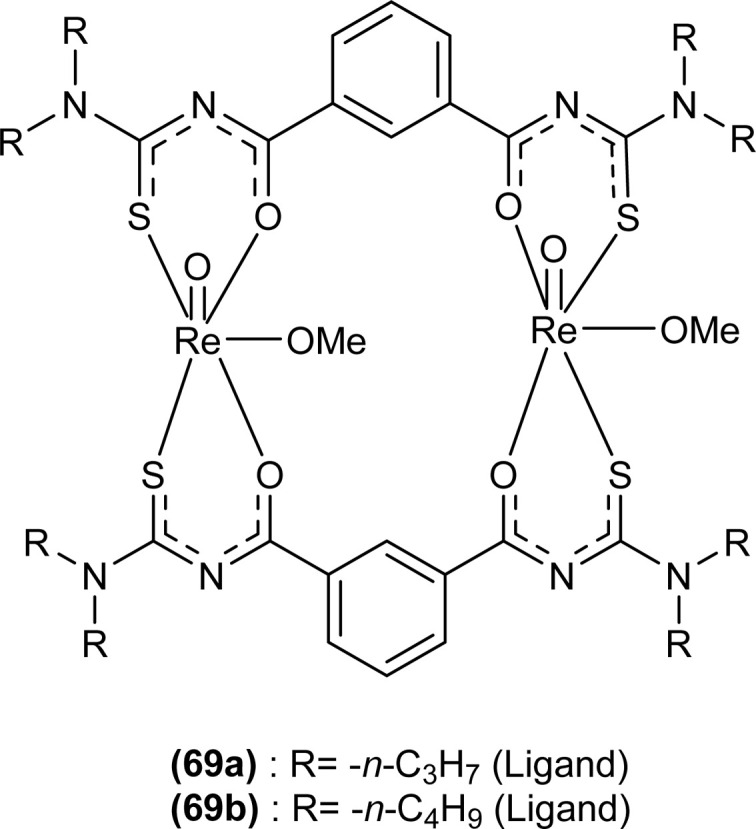
Benzoyl thiourea ligands and their di-nuclear Re(v) complexes.

Oxo rhenium(v) complexes of benzoyl thiourea ligands were synthesized by Keskin *et al. via* a reaction between acyl thiourea derivatives (69a and 69b) and tetrabutylammonium tetrachlorooxorhenate(v) in methanol (Fig. 15). The tetra-nuclear complexes were synthesized by the recrystallization of di-nuclear complexes using DCM and acetonitrile solution. Single crystal XRD analysis showed that the complexes crystallized in a triclinic crystal system and adopted the *P*1̄ space group (di-nuclear complex 70b), and monoclinic crystal system with *P*2_1_/*n* space group (tetra-nuclear complex 1). The structural isomer of the complex (70b) was obtained as an *anti*-isomer compared to di-nuclear oxorhenium(v) complexes previously synthesized. All di-nuclear oxorhenium(v) complexes exhibited octahedral geometry. Oxo and metoxo ligands lied in *trans*-axial positions, and C=S and C=O in the *cis* position defined the basal plane of the crystal system. The crystal structure of the tetranuclear rhenium(v) complex had four tetradentate benzoyl thiourea ligands, having two dimeric blocks connected by the oxo group and the complex also had two solvent molecules attached to it (MeOH) adopting distorted octahedral geometry. Inter and intramolecular H-bonding and non-covalent interactions like π–π stacking played crucial roles in the 3D network structure of complexes.^81^

**Fig. 16:**
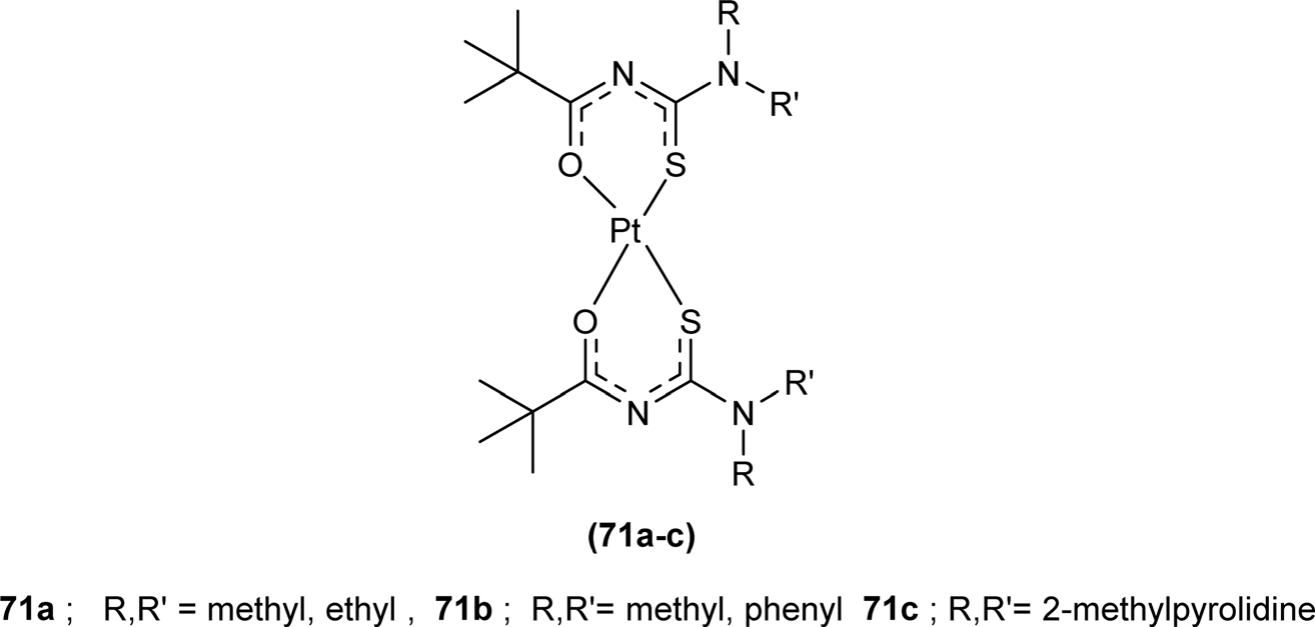
Platinum(ii) metal complexes of asymmetrically disubstituted pivaloyl thiourea.

Nkabyo *et al.* synthesized Pt(ii) complexes of the asymmetrically disubstituted pivaloyl thioureas (71a–c). From single crystal analysis, it was found that the two chelating di-substituted pivaloyl thioureas coordinated *via* sulfur and oxygen atom with central metal atom Pt(ii) (Fig. 16). Two complexes *cis*-EE-Pt-71a and *cis*-EE-Pt-71b crystallized in a monoclinic crystal system adopting *P*2_1_/*n* and *C*2/*c* space groups, respectively and having *cis*-EE structural conformation. While *cis*-Pt-71c crystallized in an orthorhombic crystal system with a *Pbca* space group and the complex adopted *cis*-ZZ structural conformation. The geometry of all three metal complexes was distorted square planar as indicated by S–Pt–O bond angles, with slight deviations probably due to the presence of bulkier substituent on nitrogen atom which causes steric effects.^82^

**Fig. 17:**
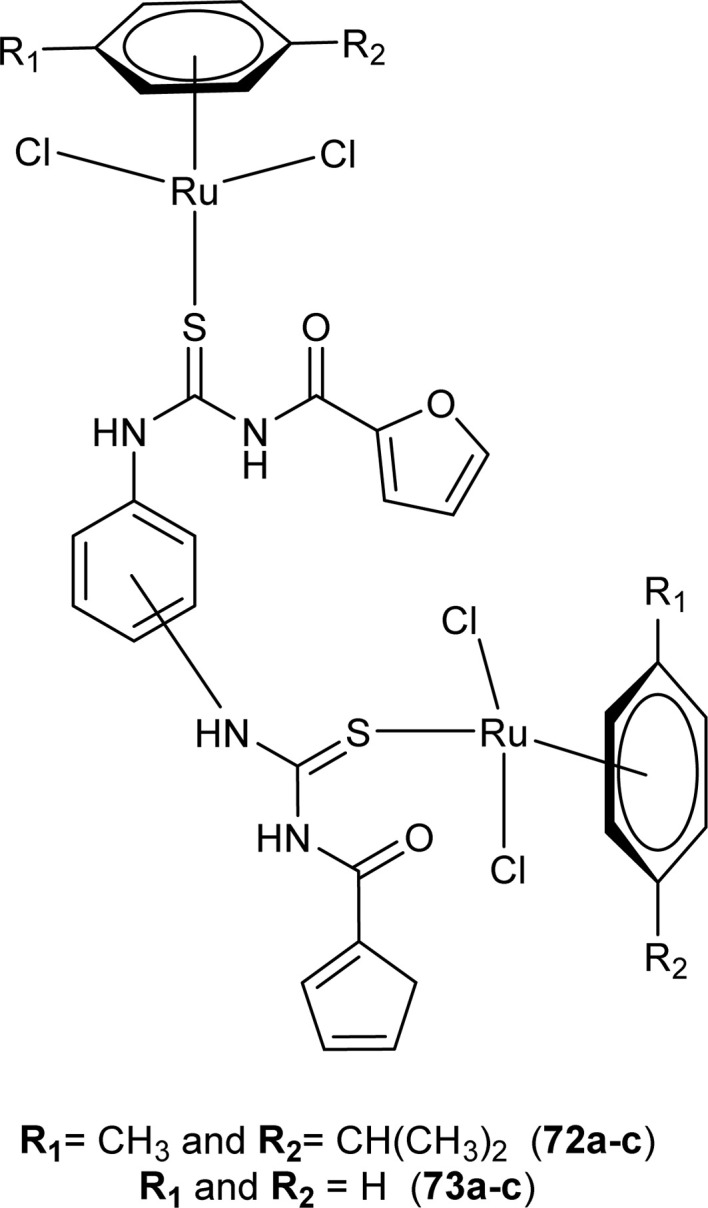
Ru(ii) complexes of bipodal furoyl thiourea ligands.

Ru(ii) complexes of bipodal furoyl thiourea ligands having *p*-cymene (72a–c) and benzene (73a–c) as arene moiety were synthesized and characterized by Swaminathan *et al.* (Fig. 17). Single crystal XRD analysis showed that the ligands L1 and L2 and complex 72c, crystallized in a triclinic crystal system adopting the *P*1̄ space group. Monodentate coordination of the ligands was due to two pseudo C_2_N_2_OH six-membered rings formation and hydrogen bonding [N(2)H⋯O(1)] is responsible for this. It was also found that there was an intramolecular hydrogen bond N–H⋯S interaction in the L1 ligand. Complex 72c showed pseudo-tetrahedral piano stool geometry consisting of Ru ions, chlorido ions and bipodal acyl thiourea ligand. DFT study for ligands L1 and L3 and complex 72c verified the structure obtained from single crystal XRD analysis.^83^

**Fig. 18:**
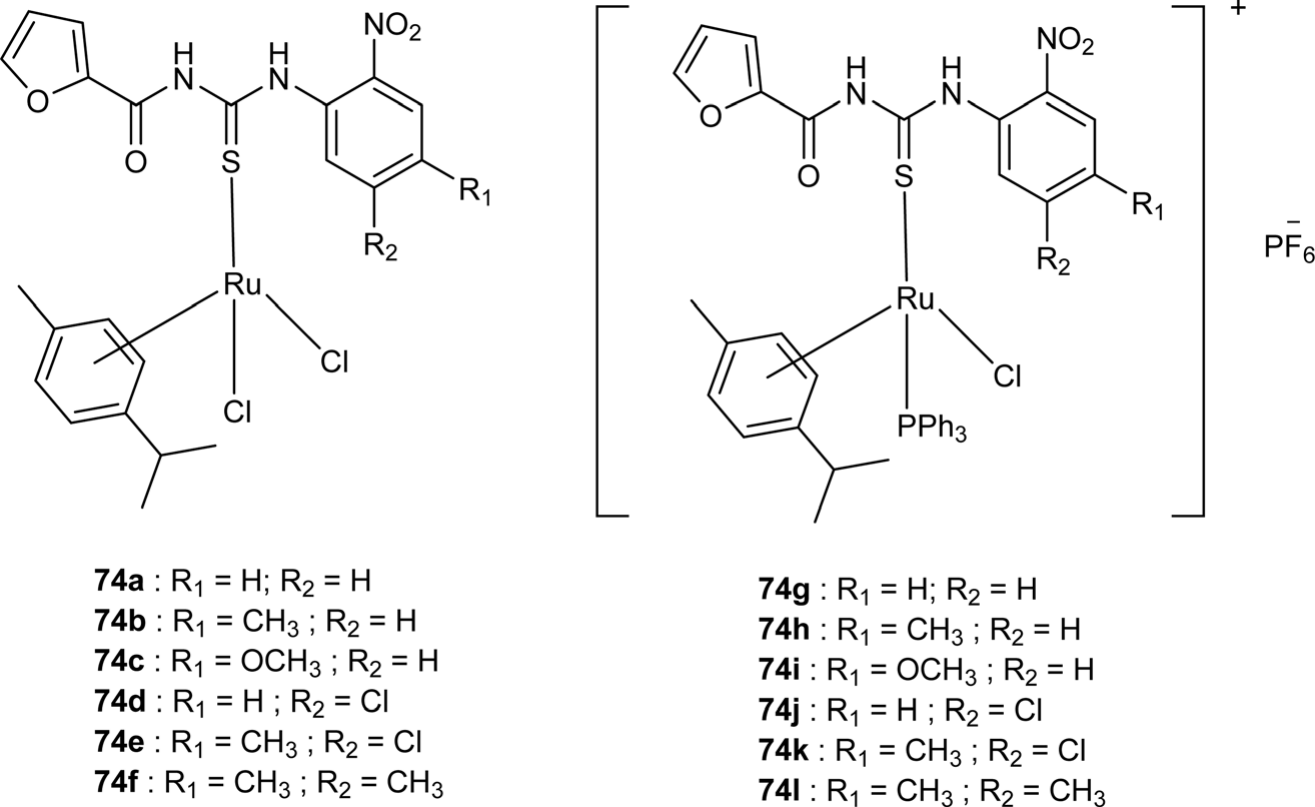
Ru(ii)-*p*-cymene complexes of furoyl thiourea ligands.

Close related Ru(ii)-*p*-cymene complexes also containing benzoyl and furoyl thiourea ligands were synthesized and characterized by Dorairaj *et al.* and Obradović *et al.* Single crystal XRD analysis showed that in complex 74a and 74c, furoyl thiourea ligands underwent bidentate coordination with Ru(ii) metal ion in complexes through S and N atoms, but in complex 74d, monodentate coordination through S atom was observed (Fig. 18). Acyl thiourea ligands L2 and L3 crystallized in a monoclinic crystal system adopting the *P*2_1_/*c* space group. Complex 74a and 74c consisted Ru(ii) ion, aroyl thiourea ligand, *p*-cymene and chlorido ligands. Intramolecular hydrogen bonding between NH, carbonyl oxygen, NO_2_ and chlorido groups were observed in crystals of complex 74a, 74c, 74d and 74f.^84,85^

**Fig. 19:**
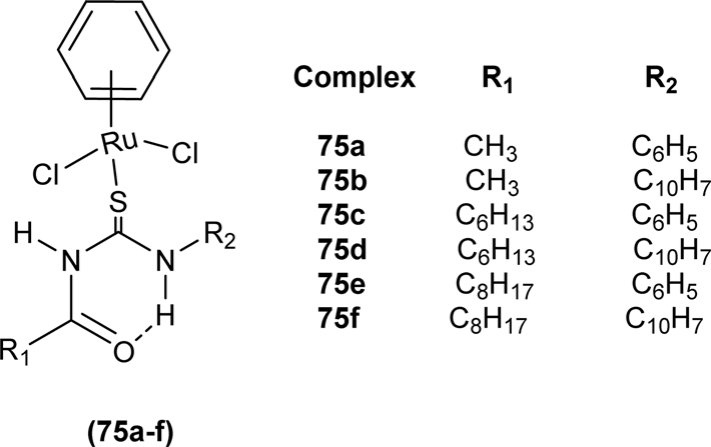
Ru(ii) benzene complexes containing acyl thiourea ligands.

Ru(ii) benzene complexes (75a–f) of acyl thiourea ligands were synthesized and characterized by Swaminathan *et al.* (Fig. 19). Conformational equilibria are present in the solution phase, while only one stable conformation was observed in the crystal, due to gaining free energy of crystallization and compact packing. XRD analysis of benzene complexes of Ru(ii) (75b and 75e) showed that acyl thiourea ligands underwent bidentate coordination with metal ions and it was further confirmed by short intramolecular hydrogen bond distance of thioamide proton and carbonyl oxygen. H-bond length in 75e (2.590 Å) was shorter as compared to 75a (2.621 Å) and 75b (2.622 Å) which showed negligible changes, it indicated the influence of the alkyl chain because it had very prominent effect on aromatic N-terminal conjugation. These all changes occurred in ligands conformation to adjust 4 members around a central metal atom in metal complexes.^86^

**Fig. 20:**
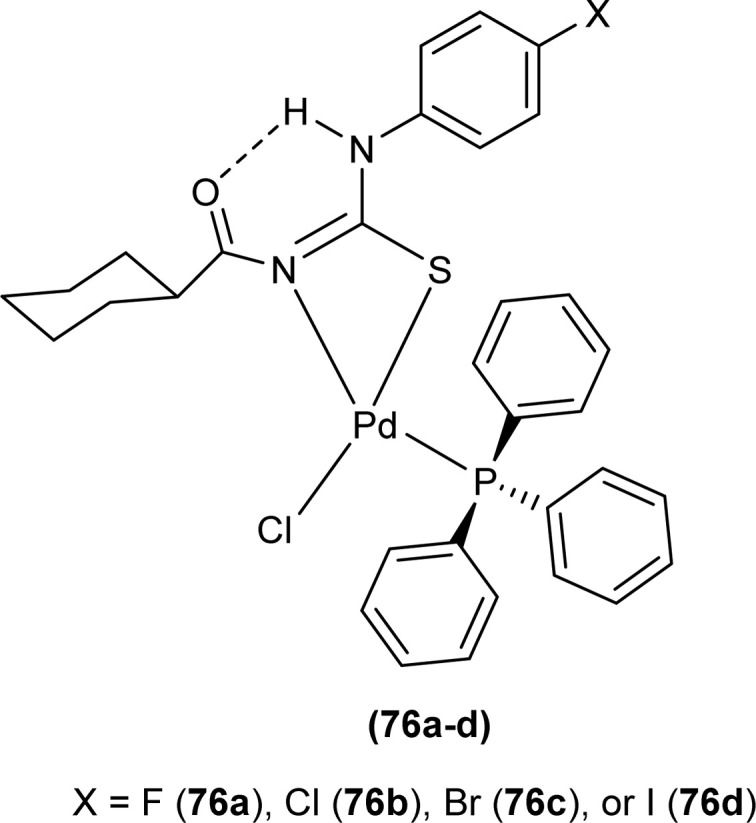
Pd(ii) complexes of acyl thioureas.

Dorairaj *et al.* synthesized Pd(ii) complexes of acyl thiourea (76a–d) and characterized them by FT-IR, NMR and single Crystal XRD. Ligand L1 crystallized in monoclinic while L3 and L4 crystallized in triclinic crystal systems with *P*2_1_/*c* and *P*1̄ space groups, respectively. Complexes (76c and 76d) crystallized in triclinic systems adopting the *P*1̄ space group, and Pd(ii) complexes existed in distorted square planar geometry. From XRD analysis it was confirmed that acyl thiourea acted as a bidentate ligand and coordinated with metal through S and N atoms, the geometry of the complex had central metal atom Pd(ii), acyl thiourea, chlorido and triphenyl phosphine ligand (Fig. 20).^87^

**Fig. 21:**
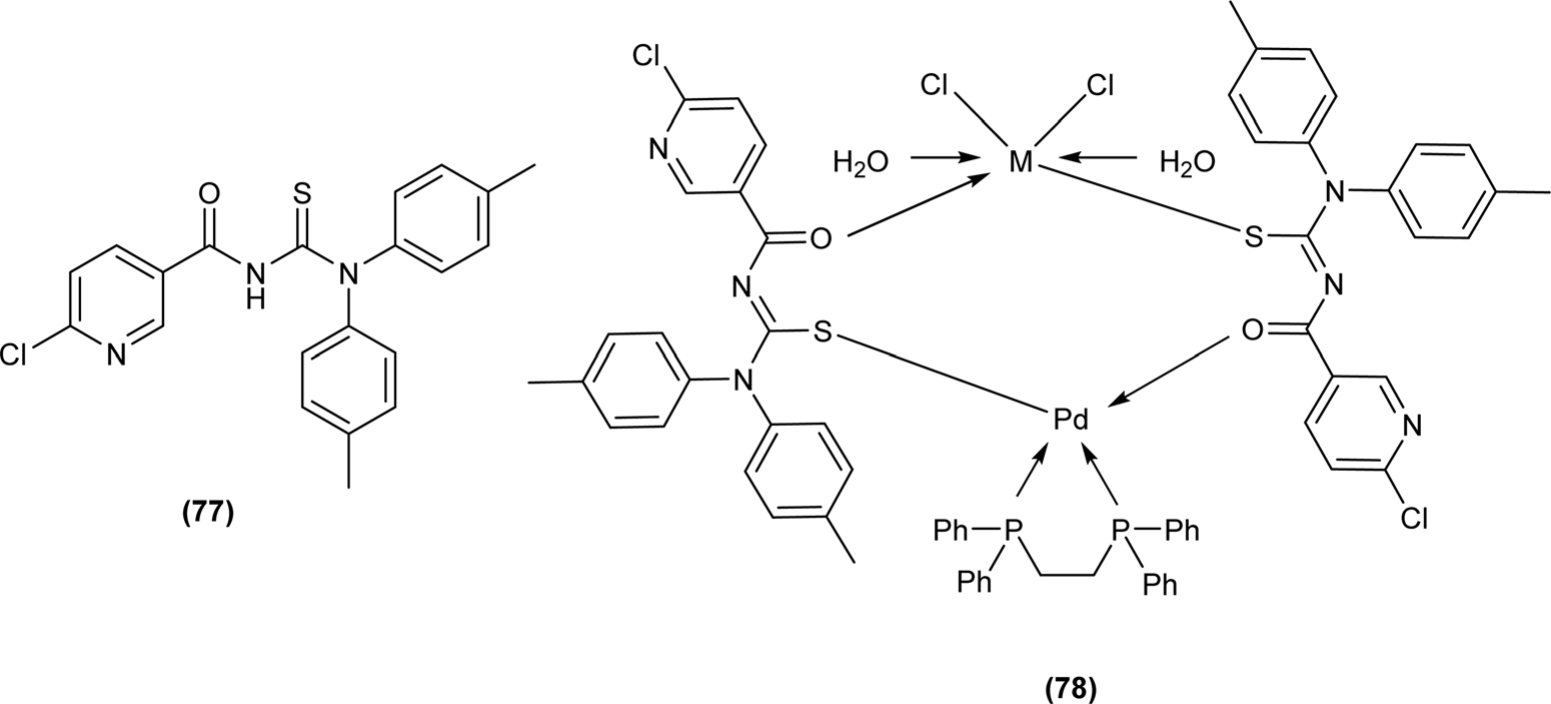
Acyl thiourea ligand and its hetero binuclear complex with palladium.

Acyl thiourea ligand (77) and its complexes with palladium metal were synthesized by Muhammed *et al.* The ligand formed monodentate and bidentate complexes with palladium metal through S and O coordinating atoms of the thiocarbonyl and carbonyl groups, respectively. Then further treating the complexes with transition metals M^2+^[with M = Zn(ii), Cd(ii), or Co(ii)] afforded hetero binuclear complexes. Ligand and complexes were evaluated for their cytotoxic and antibacterial activity. Among the synthesized compounds hetero binuclear complex (78) were found more effective against CEMSS cancer cells (Fig. 21).^88^

**Fig. 22:**
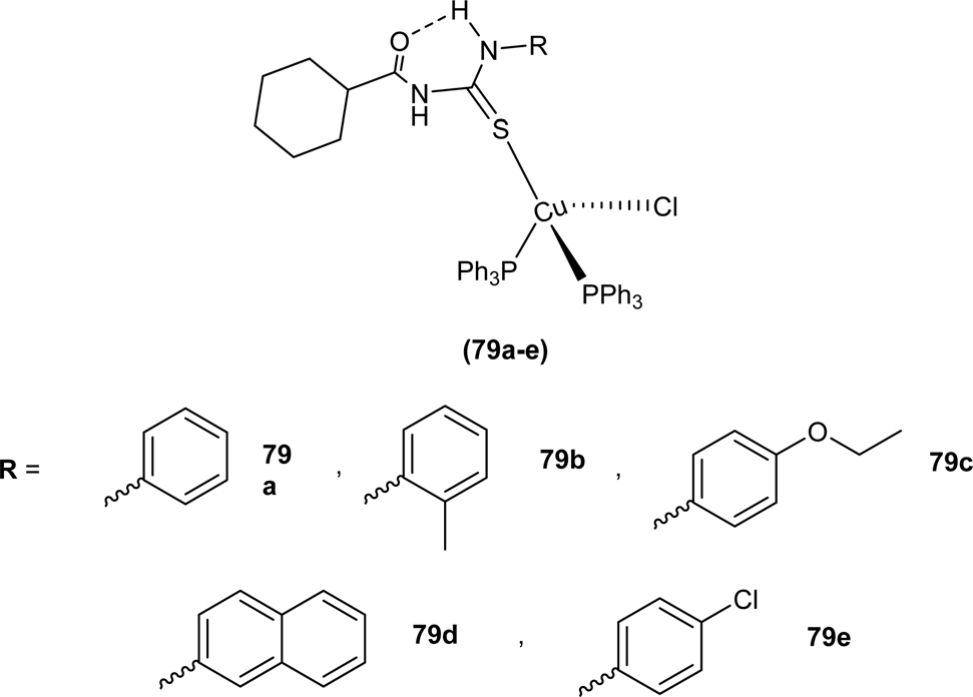
Cu(i) metal complexes of thioureas.

Novel acyl thiourea complexes (79a–e) of Cu(i) were synthesized and characterized by spectroscopic techniques and single crystal XRD by Dorairaj *et al.* All the synthesized complexes crystallized in a triclinic crystal system with a *P*1̄ space group. From XRD analysis it was found that the neutral monodentate coordination of acyl thiourea to metal ion occurred through the S atom and it was further verified from the elongation of the C–S bond compared with simple non-coordinated ligands (Fig. 22). Complexes in a crystal system adopted distorted tetrahedral geometry. Intramolecular hydrogen bonding observed in complexes was of the type N–H⋯O and N–H⋯Cl.^89^

**Fig. 23:**
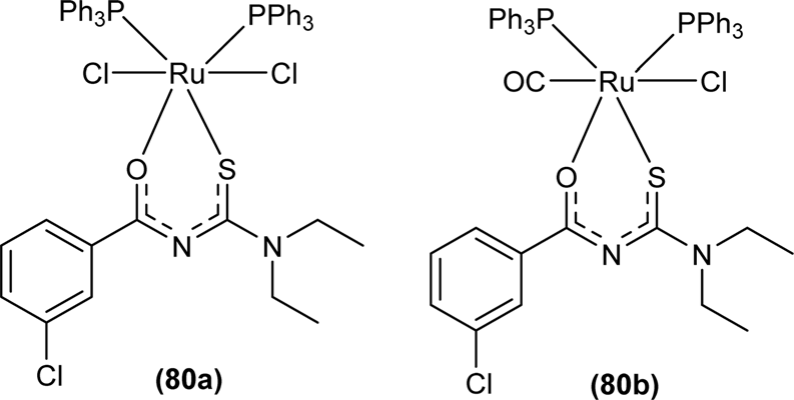
Ru(ii) and Ru(iii) complexes of acyl thiourea ligand.

Uysal *et al.* observed from XRD analysis that the Ru(ii) and Ru(iii) complexes of acyl thiourea [RuCl_2_(PPh_3_)_2_L^1^] (80a), [RuCl(CO)(PPh_3_)_2_L^1^] (80b) crystallized in orthorhombic and monoclinic crystal system with *Pna*2_1_ and *Cc* space group, respectively. It was also found that the complex (80a) adopted distorted octahedral geometry having one acyl thiourea ligand coordinated to the metal ion through S and O atoms, two chloride ions at the equatorial position, and two PPh_3_ at the axial position in solid state structure. The same is the case for complex (80b) in which the only difference was that the chloride and CO ligands were present at the equatorial position (Fig. 23).^90^

**Fig. 24:**
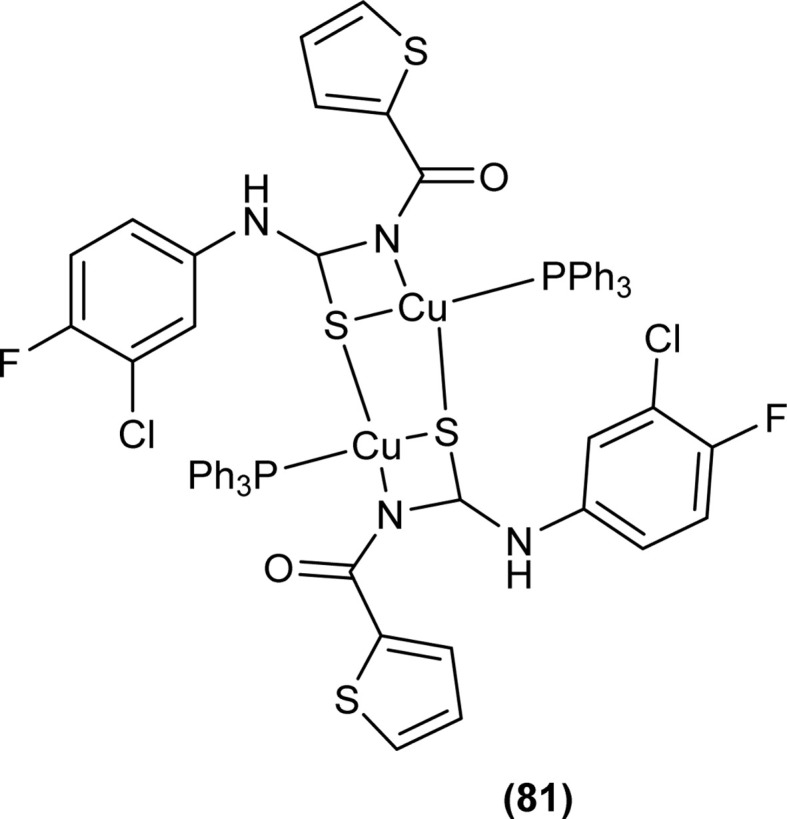
Cu(i) complex of *N*-(2-thiophenecarbonyl)-*N*′-(3-Cl,4-F-phenyl)thiourea.

Cu(i) complex of the *N*-(2-thiophenecarbonyl)-*N*′-(3-Cl-4-F-phenyl)thiourea was synthesized and characterized by FT-IR, FT-Raman, NMR and single crystal XRD analysis. XRD analysis showed that binuclear compound (81) was formed in which sulfur atom of acyl thiourea bridged the two complexes through coordination with Cu(i) of two complex molecules and adopted slightly distorted tetrahedral geometry, thus led to the formation of highly strained four-membered ring in which Cu⋯Cu bond distance is 2.997 Å. The ligand acted as κ2-N,μ-S bidentate and coordinated through S and N atoms to the central metal atom Cu(i) (Fig. 24).^91^

**Fig. 25:**
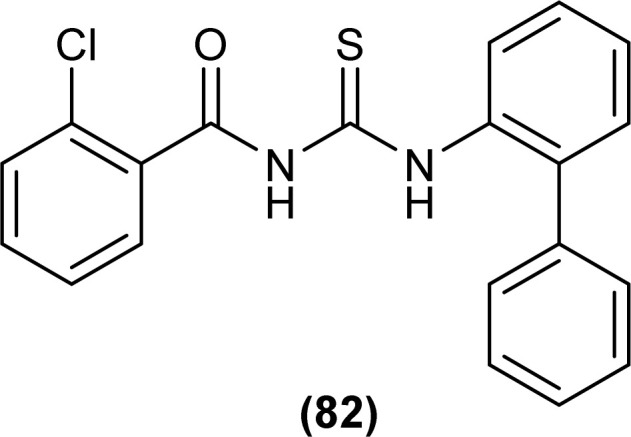
Structure of *N*-(1,10-biphenyl)-2-chlorobenzoylthiourea.

*N*-(1,10-biphenyl)-2-chlorobenzoylthiourea (82) and their metal complexes with Co(ii), Ni(ii), and Cu(ii) metal were synthesized and characterized by various spectroscopic techniques and single crystal XRD analysis. Compound (82) crystallized in a monoclinic crystal system and adopted the *P*1̄2_1_/*c*1 space group. Thermal behavior investigation of the synthesized Co(ii), Ni(ii), and Cu(ii) complexes showed that these compounds were thermally stable and remained unaffected by exposure to various temperatures like 165, 185, and 122 °C (Fig. 25).^92^

**Fig. 26:**
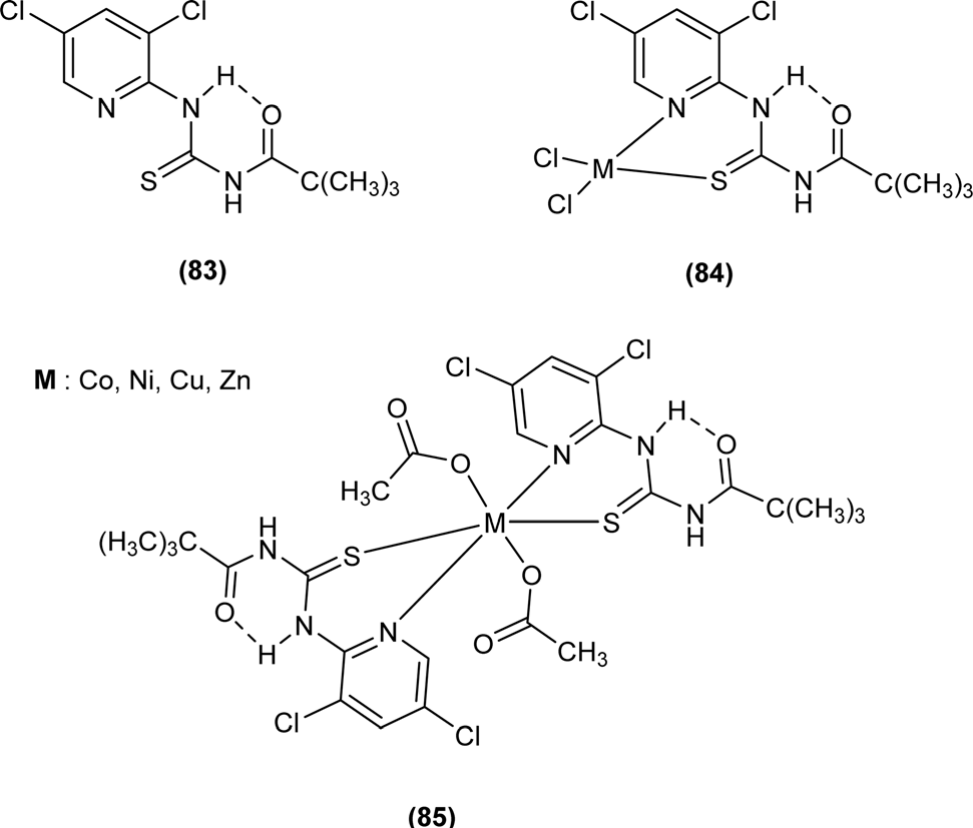
*N*-((3,5-Dichloropyridin-2-yl)carbamothioyl)pivalamide ligand and its complexes with Co(ii), Ni(ii), Cu(ii), Zn(ii).

A new ligand *N*-((3,5-dichloropyridin-2-yl)carbamothioyl)pivalamide (83) and their metal complexes with Co(ii), Ni(ii), Cu(ii), Zn(ii) were synthesized and characterized by various spectroscopic techniques. The proposed molecular structure of the synthesized complexes was reported as, (84) [metal : ligand] [1 : 1] tetrahedral and (85) [metal : ligand] [1 : 2] octahedral, where M^2+^ = Co, Ni, Cu and Zn (Fig. 26).^93^

**Fig. 27:**
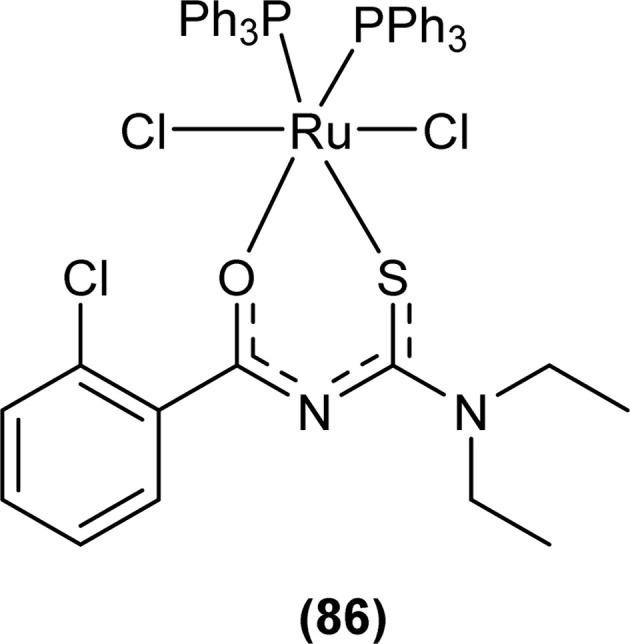
Structure of Ru(iii) complex containing 2-chloro-*N*-(diethylcarbamothioyl)benzamide ligand.

Single crystal XRD analysis of ruthenium(iii) acyl thiourea complex (86) showed that the compound [RuCl_2_(PPh_3_)_2_BTU] crystallized in a monoclinic crystal system with a *Cc* space group. In the molecular structure of the complex, the two chloride ions and acyl thiourea ligand coordinated in the anionic bidentate mode through sulfur and oxygen atoms with Ru(iii) and two chloride ions in the equatorial position, while triphenylphosphine ligands coordinated to Ru metal in axial position (Fig. 27). Crystal packing stability of the molecule was due to intra and intermolecular hydrogen bonding and weak non-covalent interactions (C–H⋯π interaction) between hydrocarbons and aromatic rings.^94^

Both, *cis* and *trans* isomers of the dichloro bis[*N*,*N*-dibutyl-*N*′-(4-chloro-benzoyl)thioureato]palladium(ii) complexes with sulfur monodentate (κS) and bidentate κ^2^S,O coordination modes were obtained by reaction between PdCl_2_ and *N*,*N*-dibutyl-*N*′-(4-chlorobenzoyl)thiourea (BTU). Both Pd(ii) complexes had been characterized by elemental analysis, FT-IR, ^1^H NMR, ^13^C NMR, and UV-vis techniques, together with X-ray single-crystal diffraction. The palladium complexes were also applied as an efficient catalyst for the Suzuki–Miyaura cross-coupling reaction of aryl halides with aryl boronic acids.^95^

## 6. Applications

### 6.1 Biological aspects

In recent decades, there has been a considerable growth in interest surrounding thioureas, an important class of organic compounds. Scientists are intrigued by the biological properties exhibited by 1-(acyl/aroyl)-3-(substituted)thiourea derivatives, as evidenced by numerous scholarly articles dedicated to their investigation. This area of research has emerged as particularly compelling, with researchers consistently exploring modifications to the structures of thiourea and its analogs in pursuit of novel biological activities. In the ensuing section, we will delve into the diverse biological activities associated with thioureas and their analogs.

#### 6.1.1 Antibacterial activity

**Fig. 28:**
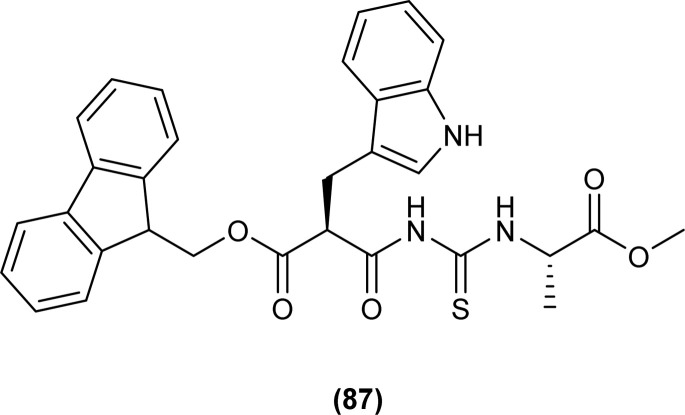
Indole linked acyl thiourea.

A series of acyl thioureas synthesized by Shankraiah *et al.* showed antibacterial activity against *Escherichia coli* and *Staphylococcus aureus*, among the synthesized derivatives, compound (87) was found more potent compared to the standard Streptomycin Sulphate. Keto-thiourea moiety present in the acyl thioureas was pointed responsible for the antibacterial activity of the synthesized compounds (Fig. 28).^39^

**Fig. 29:**
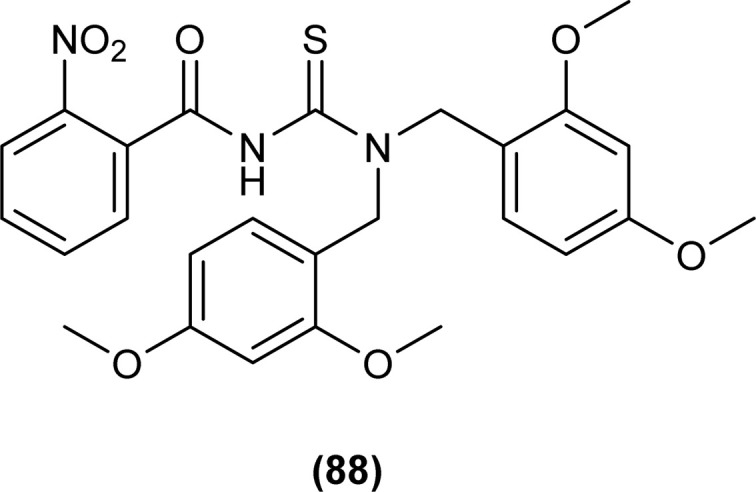
Structure of *N*,*N*-disubstituted-*Ń*-acylthiourea.

Novel acyl thiourea was synthesized and evaluated for its antibacterial and antifungal activity by Arslan and Binzet. The synthesized compound (88) exhibited more potency as an antifungal agent against *Candida parapsilosis*, and *Candida metapsilosis* strains compared to antibacterial activity observed against *Staphylococcus aureus* and *Streptococcus pneumonia* (Fig. 29).^62^

**Fig. 30:**
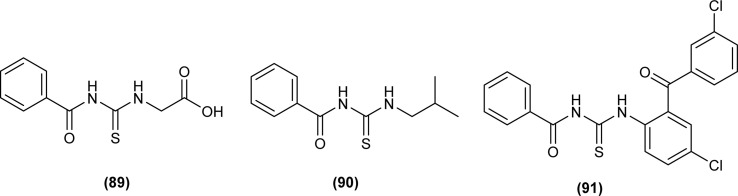
Structure of acyl thioureas evaluated for antibacterial potential.

Amongst the series of acyl thiourea and their metal complexes synthesized by Shah *et al.*, the compounds (89, 90 and 91) were found to be more potent against *K. pneumoniae* and *P. aeruginosa*, respectively (Fig. 30). In the case of their metal complexes, the copper complexes exhibited less potency, whereas cobalt complexes showed better potency compared to parent ligands. The different activity of metal complexes compared to parent ligands was attributed to different physicochemical properties, which led to different bindings with the bacterial cell membranes.^76^

**Fig. 31:**
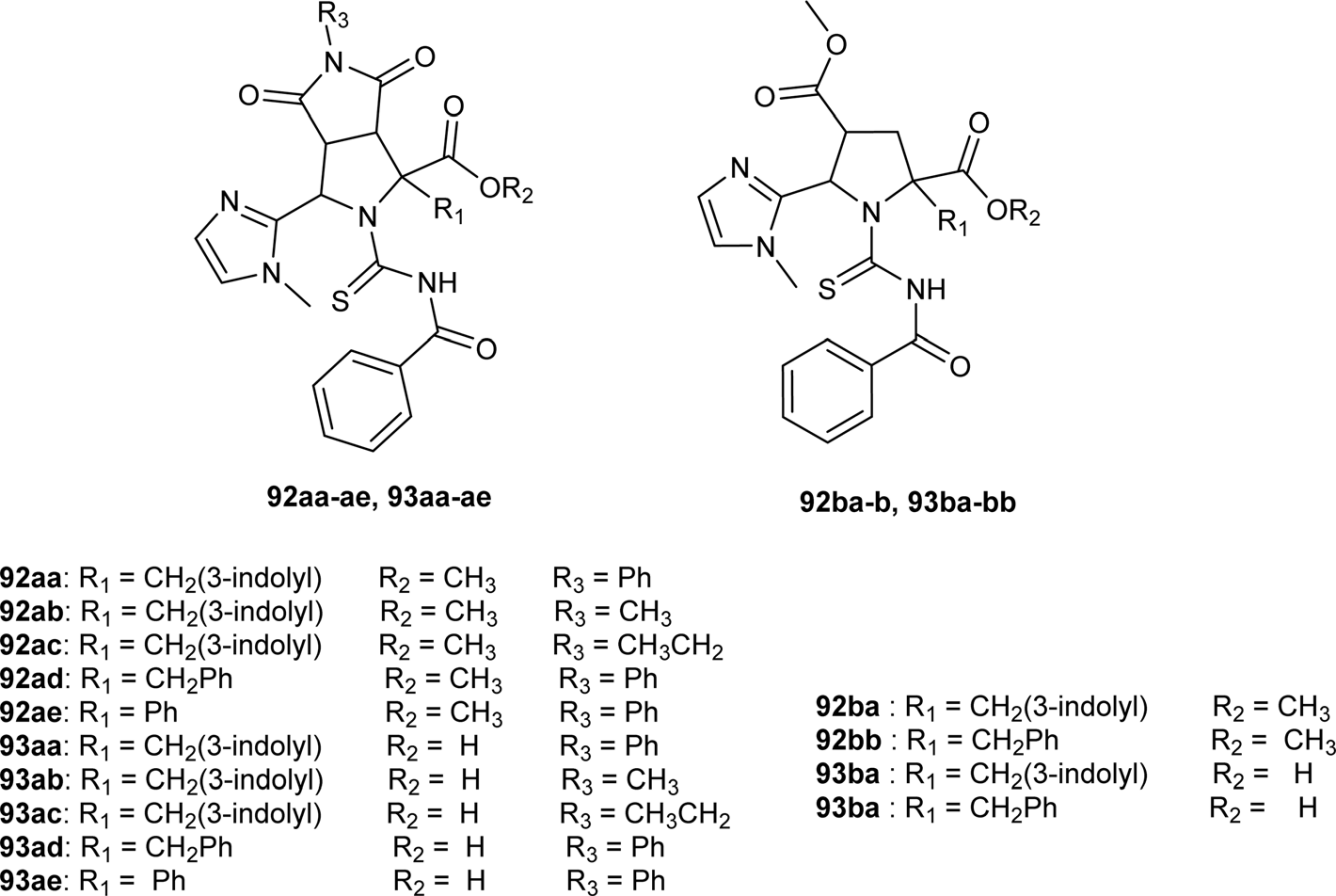
Pyrrolidine derivatives and *N*-benzoyl thiourea pyrrolidine carboxylic acid derivatives.

From an antibacterial activity study of acyl thiourea derivatives synthesized by Poyraz *et al.*, it was found that pyrrolidine derivatives (92aa–ae, 92ba–bb) and *N*-benzoyl thiourea pyrrolidine carboxylic acid derivatives (93aa–ae, 93ba–bb) having MIC values in the range of 62.50–250 μg mL^−1^, were less potent against Gram-positive (*Staphylococcus aureus*, *Bacillus subtilis*) and Gram-negative (*Aeromonas hydrophila*, *Escherichia coli*). All these compounds showed similar activity as the reference against *Acinetobacter baumannii* except compound 92ac, whereas only compounds 93aa and 93ba showed higher potency with MIC value of 62.50 μg mL^−1^ compared to the reference (Fig. 31).^63^

**Fig. 32:**
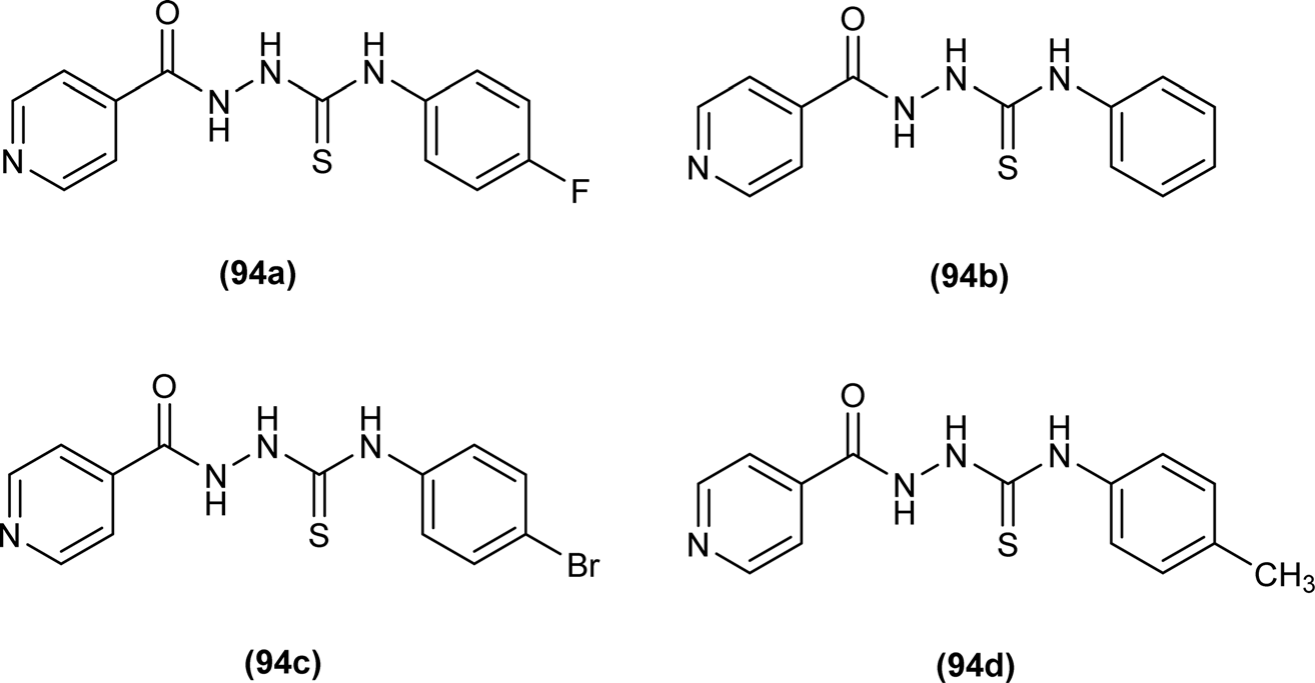
Isoniazid-based acyl thiourea derivatives.

Isoniazid-based acyl thiourea (94a–d) were synthesized and characterized by Ramaswamy *et al.* and investigated for their antibacterial activity against Gram-positive (*B. subtilis*, *S. aureus*) and Gram-negative bacteria (*E. coli*, *K. pneumoniae*). Among acyl thiourea derivatives, compounds 94a and 94c having fluoro and bromo groups, respectively were found more potent compared to reference. Compound 94a showed good activity only against Gram-negative bacteria (*K. pneumoniae*). Molecular docking studies showed that the target center for the molecule was the *E. Coli* DNA Gyrase A protein. It was also found that Compound 94a interacted with (Ser and Arg) amino acids of protein with binding energy −9.7 kcal mol^−1^ (Fig. 32).^96^

**Fig. 33:**
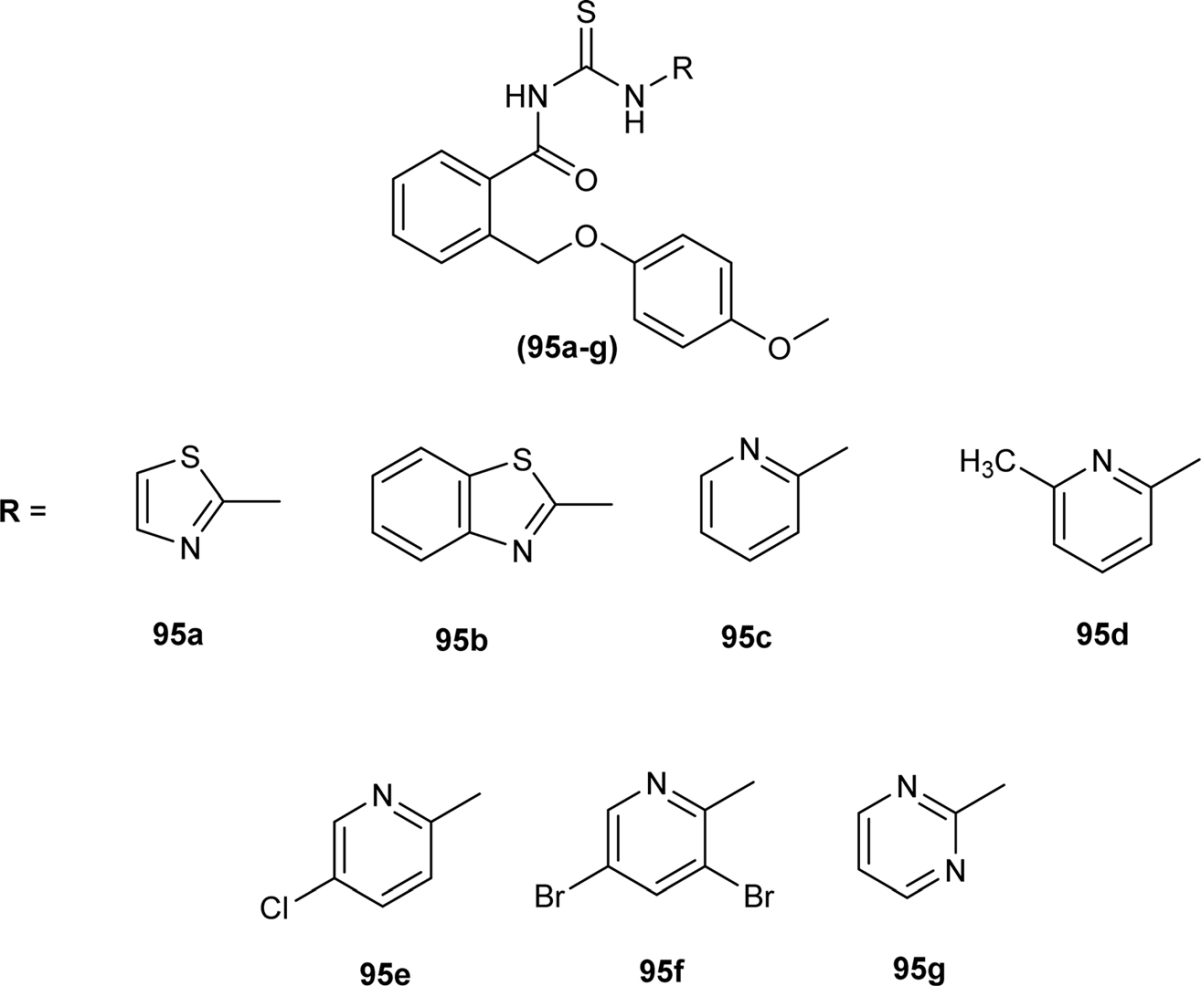
1-(benzyloxy)-4-methoxybenzene based acyl thioureas.

Novel acyl thioureas (95a–g) were synthesized and evaluated for antimicrobial activity against *Staphylococcus aureus*, *Enterococcus faecalis*, *Escherichia coli* and *Pseudomonas aeruginosa* by Roman *et al.* MIC values were found to be very high and between 1250 and 5000 μg mL^−1^ for the synthesized compounds as compared to standard antibiotic Ciprofloxacin (MIC values of 0.012–0.62 μg mL^−1^). All the compounds showed negligible activity against the above bacterial strains. However, these *N*-substituted acyl thioureas exhibited remarkable antibiofilm activity (MBIC values between >5000 and 625 μg mL^−1^) against the above strains of bacteria and amongst the series compounds 95b and 95d were found to be the most potent against *E. coli* with MBIC value of 625 μg mL^−1^ (Fig. 33).^97^

**Fig. 34:**
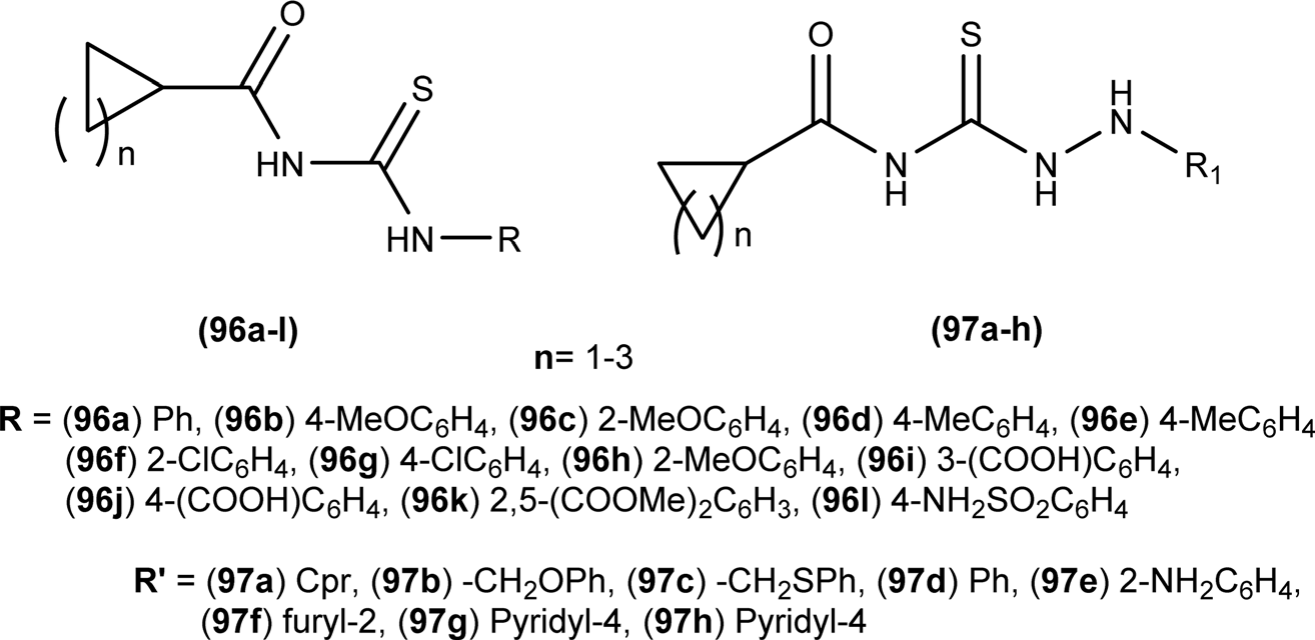
1-Cycloalkanecarbonyl-substituted acyl thioureas and thiosemicarbazides.

Anti-bacterial activity of acyl thioureas and thiosemicarbazides was investigated by Kholodniak *et al. N*-substituted acyl thioureas (96a, 96b, 96c, 96e, 96j, and 96l) were found to exhibit poor inhibitory activity towards DHFR but showed moderate antibacterial activity against *E. coli* (MIC 100 μg mL^−1^, MBC 200 μg mL^−1^), *Ps. aeruginosa* and *St. aureus* (MIC 50 μg mL^−1^, MBC 100 μg mL^−1^). Thiosemicarbazides (97a–h) which exhibited high activity against DHFR were also found to be potent antibacterial agents against *E. coli* (MIC 3.125–50 μg mL^−1^, MBC 6.25–100 μg mL^−1^) and *St. aureus* (MIC 6.25–100 μg mL^−1^, MBC 12.5–100 μg mL^−1^) (Fig. 34). Thiosemicarbazide derivatives also showed moderate activity against *Ps. aeruginosa* (MIC 50 μg mL^−1^, MBC 100 μg mL^−1^).^98^

**Fig. 35:**
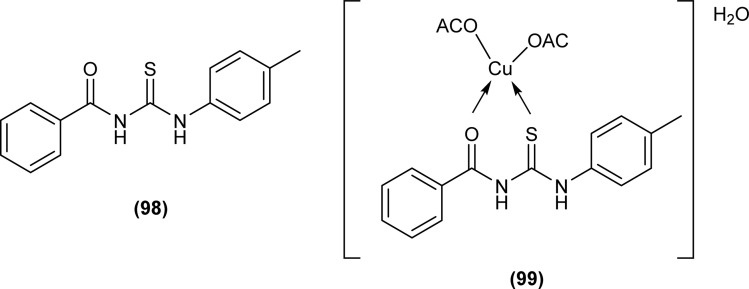
*N*-(*p*-Methylphenyl)-*N*′-benzoyl thiourea and its complex with Cu(ii) metal.

Wahdan *et al.* synthesized *N*-(*p*-methylphenyl)-*N*′-benzoyl thiourea (98) and its complex (99) with Cu(ii) metal (Fig. 35). The antibacterial activity of the synthesized compound and its metal complex was investigated against *S. aureus*, *Streptococcus* as Gram-positive bacteria, *E. coli* and *P. klebsiella* as Gram-negative bacteria. It was concluded that the metal complex exhibited higher activity than the acyl thiourea ligand which was attributed to the high penetration ability of the complex across the membrane.^99^

**Fig. 36:**
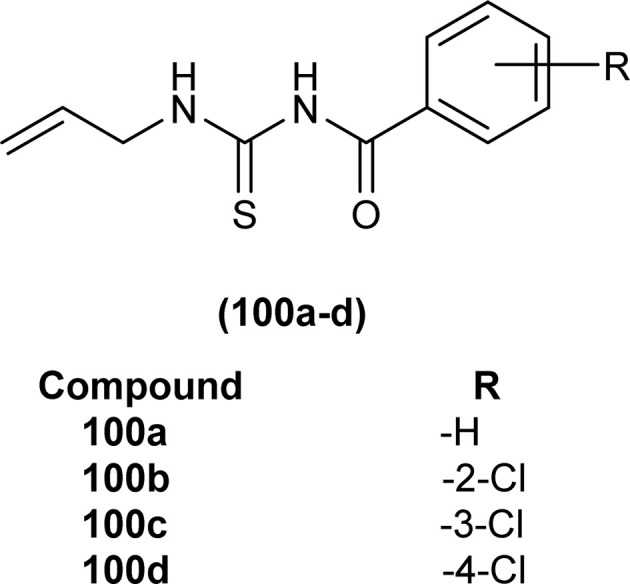
1-Allyl-3-benzoylthiourea derivatives.

Four novel analogs of 1-allyl-3-benzoylthiourea (100a–d) were synthesized and evaluated for their antibacterial activity against *S. aureus*, *S. typhi*, *E. coli*, and *P. aeruginosa* (Fig. 36). All the synthesized acyl thioureas showed very poor antibacterial activity having MIC value greater than 1000 μg mL^−1^. Only (100a) and (100d) showed comparatively better activity against *S. aureus* with a MIC value of 1000 μg mL^−1^.^100^

**Fig. 37:**
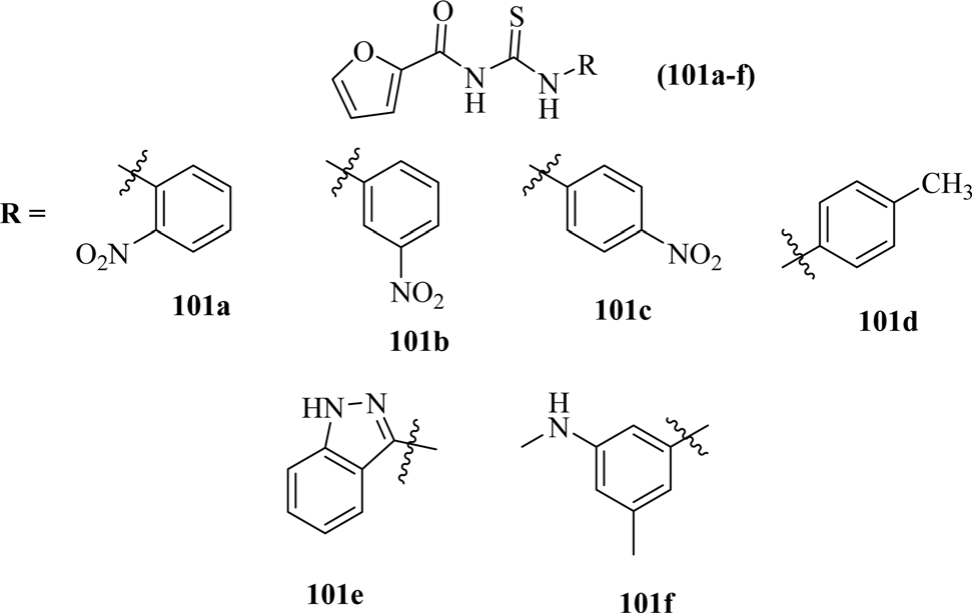
Structures of carbamothioyl-furan-2-carboxamides.

Carbamothioylfuran-2-carboxamide derivatives (101a–f) were investigated against bacterial strains (*S. aureus*, *E. coli*, and *B. cereus*). Compound (101f) showed antibacterial activity against all the tested strains with MIC values in the range of 230–295 μg mL^−1^, whereas derivatives (101a, 101b and 101c) exhibited activity against two bacterial strains *E. coli* and *B. cereus* with MIC values in the range of 240–280 μg mL^−1^. Derivatives (101d) and (101e) showed activity against *S. aureus* and *E. coli*, respectively (Fig. 37).^101^

**Fig. 38:**
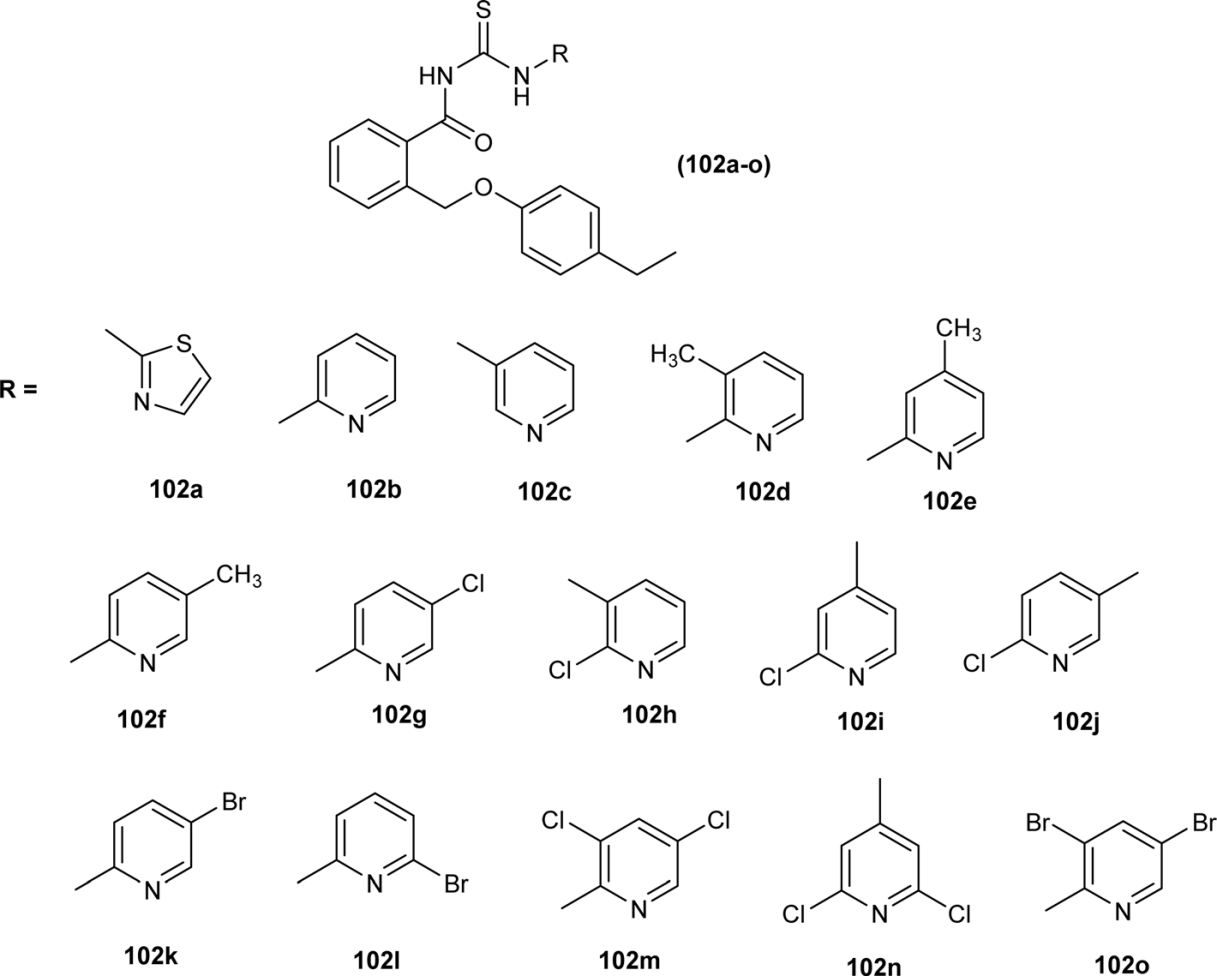
Structures of aroyl thioureas 102a–o.

Antimicrobial activity of a novel series of acyl thiourea derivatives (102a–o) was investigated against Gram-negative bacteria (*E. coli* and *P. aeruginosa*) and Gram-positive bacteria (*S. aureus* and *E. faecalis*), and the results suggested that all compounds did not exhibit prominent antibacterial activity against bacterial strains with MIC values in the range of 2500 to 625 μg mL^−1^ (Fig. 38). Among acyl thiourea derivatives compounds (102a, 102g, 102h, and 102o) exhibited better antimicrobial activity with a MIC value of 625 μg mL^−1^ against the bacterial strains *S. aureus*, and *P. aeruginosa*. All these compounds also exhibited low anti-biofilm activity except compound (102g) with an MBIC value of 312 μg mL^−1^ against *E. coli*.^102^

**Fig. 39:**
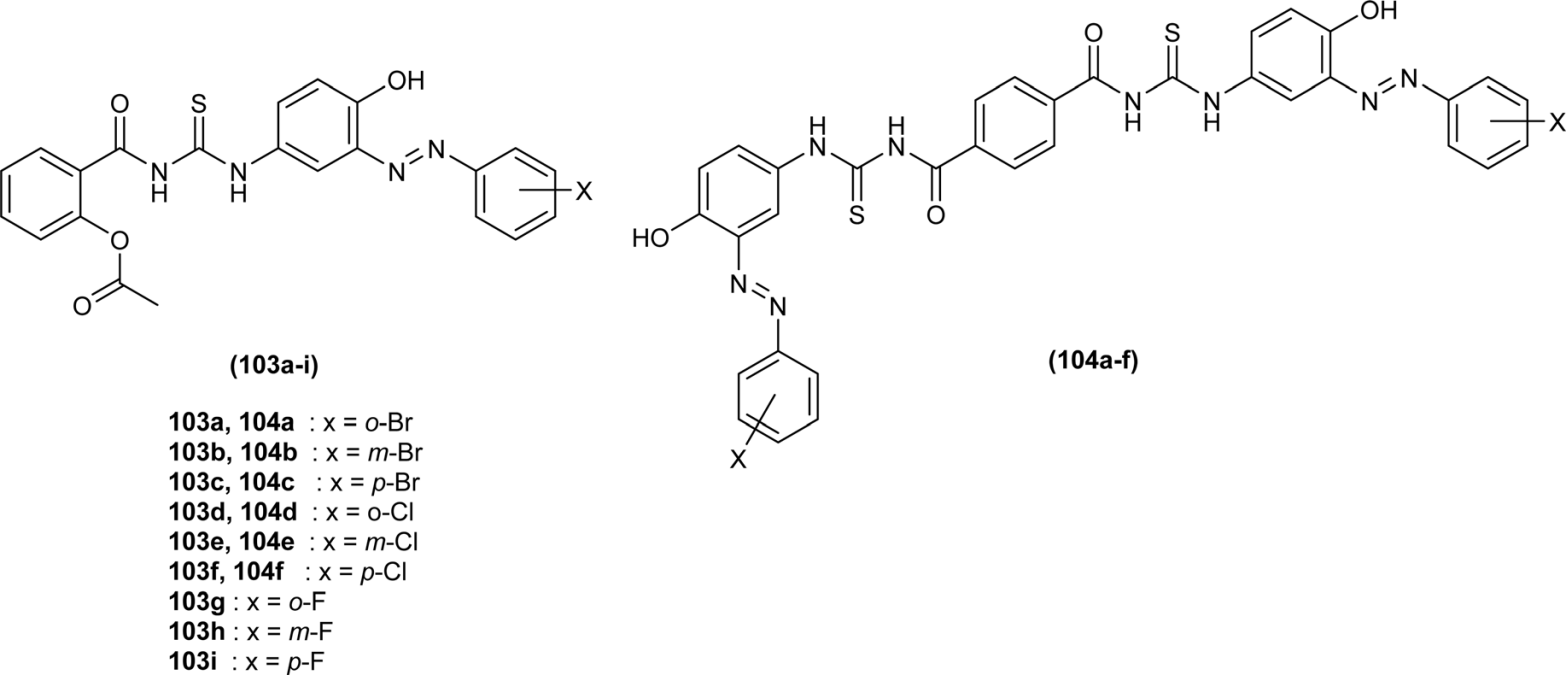
Halogenated azo-based mono and bis-acyl thiourea derivatives.

Mono and bis acyl thiourea derivatives (103a–i and 104a–f) having azo-derived paracetamol moiety were investigated against two bacterial strains *E. coli* and *S. aureus*. Mono acylated thiourea (103a–i) showed the best inhibition activity against *S. aureus*, and among them, compound 103a exhibited comparable activity to standard drug Ampicillin, the highest activity of compound 103a was due to the presence of Br atom at *ortho* position as it strongly interacted with active sites of bacterial cells. Bis acyl thiourea (104a–f) did not exhibit any antimicrobial activity against both strains *E. coli* and *S. aureus* because of steric factors caused by bulky aromatic rings preventing the desired interactions (Fig. 39).^103,104^

**Fig. 40:**
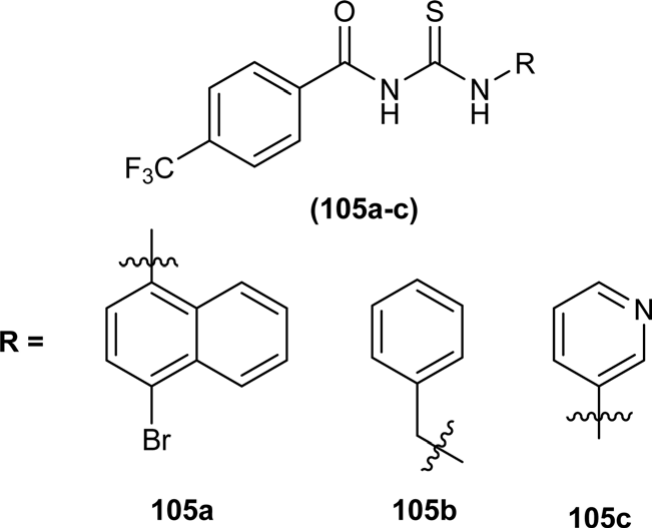
4-(Trifluoromethyl)phenyl based thioureas.

Three novel fluorinated acyl thioureas (105a–c) were synthesized and investigated for their antibacterial activity against Gram-negative (*P. aeruginosa* and *E. coli*) and Gram-positive (*B. cereus* and *S. aureus*) bacteria (Fig. 40). In the case of Gram-negative bacteria only, 105a and 105c were found more potent against *S. aureus*. All compounds showed poor activity against *B. cereus* with minimal cell inhibition of about 1–3%. None of the derivatives showed any activity against *E. coli*, only compound 105a inhibited *P. aeruginosa* up to about 20%.^105^

2-Aminothiazole derivatives (34F1-30) were evaluated for their antibacterial activity against four strains of bacteria *Xanthomonas oryzae* pv. *oryzae* (*Xoo*), *Pseudomonas syringae* pv. *actinidiae* (*Psa*), *Xanthomonas axonopodis* pv. *citri* (*Xac*), and *Xanthomonas oryzae* pv. *citri* (*Xoc*). All the series exhibited good to excellent antibacterial activity with inhibition rate (%) ranges from 24.8 ± 5.4–73.8 ± 4.4, 22.6 ± 1.6–73.1 ± 1.3, 19.4 ± 5.3–69.6 ± 2.7 and 33.0 ± 4.5–100 ± 0.0 at 100 μg mL^−1^ for *Xoo*, *Psa*, *Xac* and *Xoc*, respectively. Compound (34F29) exhibited outstanding antibacterial activity against *Xanthomonas oryzae* pv. *oryzicola* (*Xoc*) with EC_50_ value as low as 2.0 μg mL^−1^ (Scheme 9).^53^

Muhammed *et al.* synthesized acyl thiourea ligand (77) and its complexes with palladium to investigate its antibacterial potential against *E. coli*, *S. sciuri*, and *S. aureus*. Among compounds the complex [Pd(L)_2_(dppp)] showed the highest activity against *S. aureus* and *S. sciuri* with inhibition zones of 14.27 mm and 17.29 mm, respectively. This activity was due to the interaction of transition metal with the thiol (–SH) group of enzymes, leading to deactivation of enzymes (Fig. 21).^88^

**Fig. 41:**
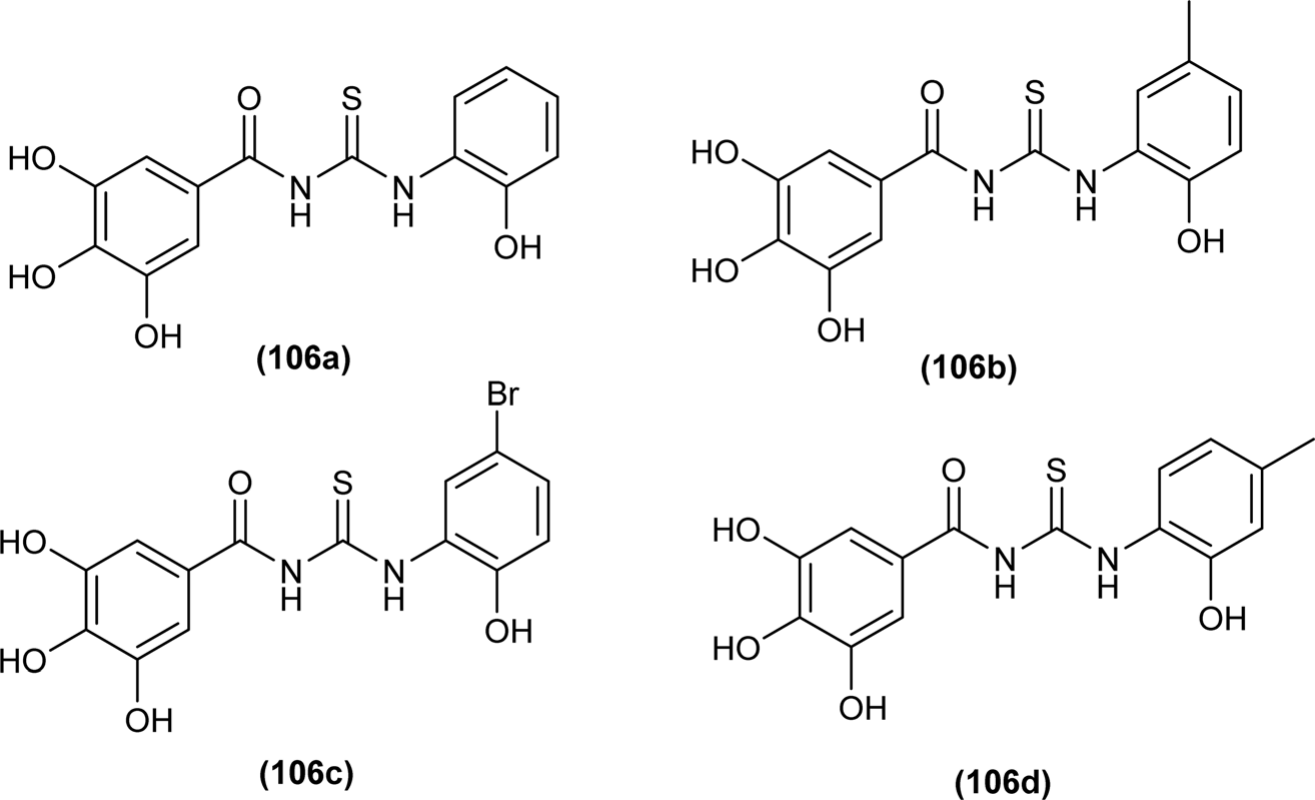
Gallic acid derivatives containing acyl thiourea phenol.

A series of phenol-containing acyl thiourea derivatives of Gallic acid were synthesized by Wu *et al.* and evaluated for their antibacterial activity against different Vibrio strains. Among the series compounds (106a and 106b) exhibited prominent inhibitory potential against *Vibrio harveyi*, with MIC 0.0156 mg mL^−1^. whilst compounds (106c and 106d) were found effective against *Vibrio cholera* and *Vibrio parahaemolyticus* having MIC values of 0.0313 mg mL^−1^ and 0.0156 mg mL^−1^, respectively. Some of the synthesized derivatives showed weak inhibitory potential against *Vibrio vulnificus* with a MIC value of 0.125 mg mL^−1^ (Fig. 41).^106^

#### 6.1.2 Antifungal activity

**Fig. 42:**
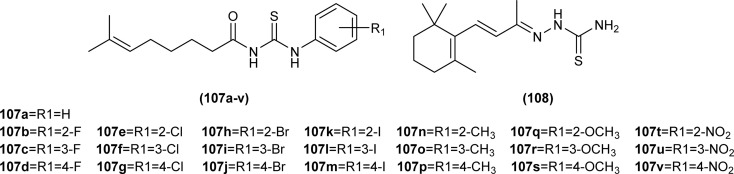
Structures of citral acyl thiourea derivatives.

Citral thiourea derivatives were synthesized and evaluated for their antifungal activity against *Colletotrichum gloeosporioides* by Zeng *et al.* Compounds (107a, 107c, 107f, 107e, and 108) showed improved activity against *C. gloeosporioides* with 0.160, 1.66, 1.37, 4.76, and 4.60 mg L^−1^ EC_50_ values, respectively. Compound (108) was far more potent than other active compounds of series against *C. gloeosporioides* and its inhibition rate was higher than 50% at a concentration of 0.24 mg L^−1^ which increased up to 95% at 7.81 mg L^−1^. The presence of an amino group in the compound (108) might be responsible for its enhanced activity. The position and nature of substituents greatly affected the antifungal activities of compounds with compounds having electron-withdrawing substituents like fluoro at *meta* position having better activities compared to other electron donating groups. Compound (108) also showed better *in vivo* antifungal activity against *C. gloeosporioides* than Carbendazim with protective efficacy values 78, 41.5, and 29.3% when used at a concentration of 100, 50, and 25 mg L^−1^, respectively (Fig. 42).^107^

Carbamothioylfuran-2-carboxamide derivatives (101a–f) exhibited prominent antifungal activity against *F. brachygibbosum*, *A. niger* and *A. flavus* with MIC values in the range of 120.7–190 μg mL^−1^ (Fig. 37).^101^

**Fig. 43:**
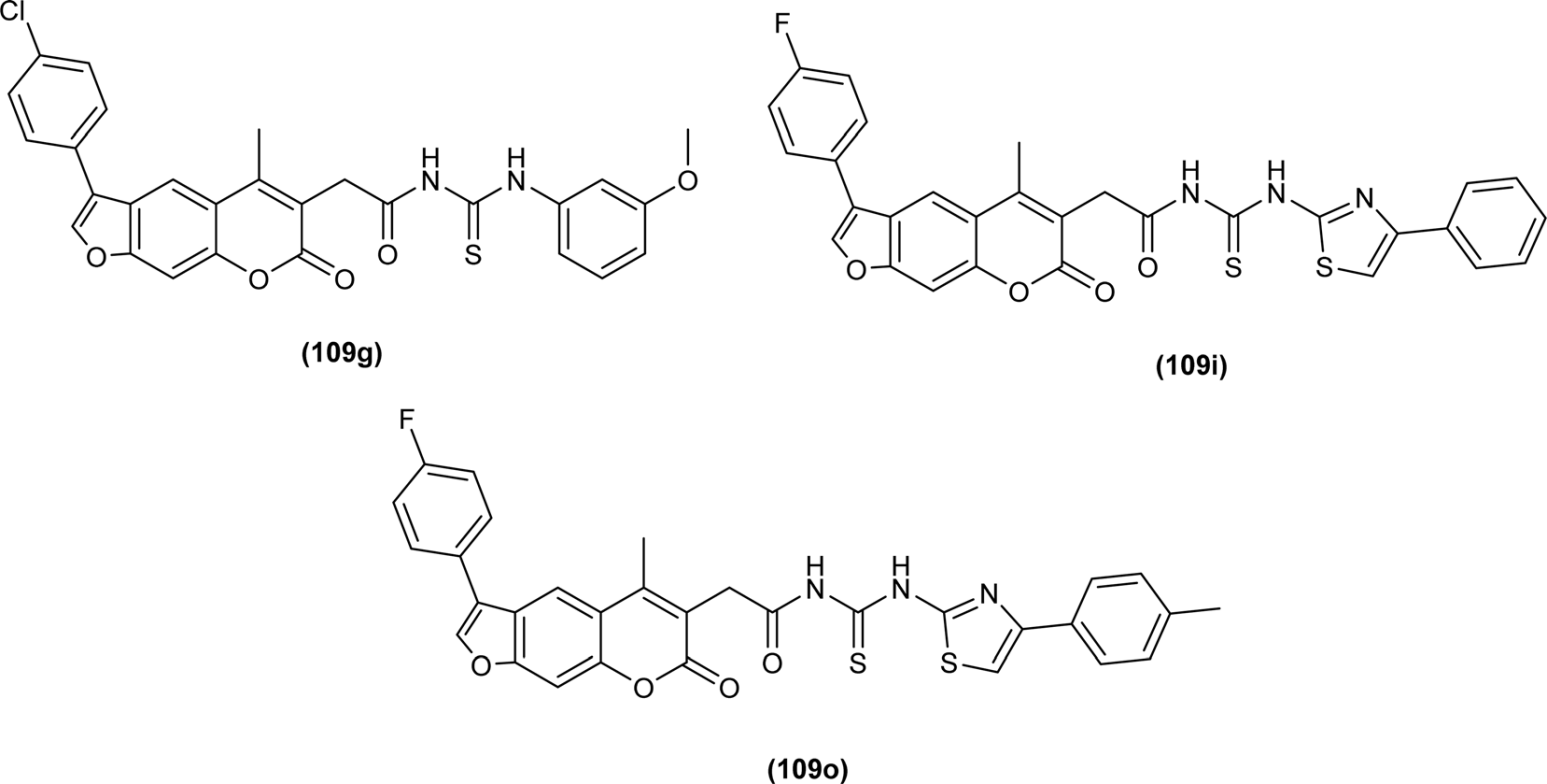
Psoralen-based acyl thiourea conjugates.

A series of Psoralen (linear furanocoumarins) based acyl thiourea conjugates (109a–u) were synthesized and evaluated for antifungal activity against *A. solani*, *B. cinerea*, *G. zeae* and *P. piricola* (Fig. 43). Compounds (109h, 109i, 109l, 109o, 109p and 109t) showed 60% inhibition activity against *B. cinerea*, while compounds (109i and 109o) with EC_50_ values of 9.09 μg mL^−1^ and 10.09 μg mL^−1^ exhibited more than 90% inhibition. Further compound (109g) exhibited prominent inhibition activity against *A. solani*, *G. zeae* and *P. piricola* with 82%, 71% and 78% inhibition, and EC_50_ values of 15.26, 27.26 and 19.16 μg mL^−1^, respectively (Fig. 43).^108^

Ligand *N*-((3,5-dichloropyridin-2-yl)carbamothioyl)pivalamide (83) and their metal complexes exhibited antifungal activity against fungi strains *Candida* and *Aspergillus*, diameter of inhibition zone ranged from 19–40 mm at 25 mg mL^−1^ for *Candida* while for *Aspergillus* it was in between 12 and 35 mm. The complex of ligand L^2^ with Zn(ii), (L)^2^ ZnAC (84) was found most potent inhibitor of *Candida* and *Aspergillus* with a diameter of inhibition zone being 40 and 35 mm, respectively (Fig. 26).^93^

2-Aminothiazole derivatives (34F1-30) were investigated against ten pathogenic fungi, results showed that some of the compounds exhibited better antifungal activity toward some types of fungi than the fungicide Carbendazim. Amongst the series compound (34F8) was found most potent inhibitor of *Phytophthora parasitica* var. *nicotianae* have comparable activity with that of standard fungicides (Scheme 9).^53^

#### 6.1.3 Antituberculosis

Acyl thiourea derivatives synthesized by Poyrazet *et al.* were investigated against the *M. Tuberculosis* H37Rv strain. MIC values of the compounds (92aa–ae, 92ba–bb) were in the range of 15.62–31.25 μg mL^−1^. Compound (92bb) having imidazole group along with benzyl group was most effective against *M. Tuberculosis* H37Rv strain with MIC value of 15.62 μg mL^−1^ (Fig. 31).^63^

Novel series of acyl thiourea derivatives (48–52) were synthesized and investigated for their antituberculosis activity against H37Rv, ATCC35822 (INH resistant), ATCC35838 (RIF resistant), ATCC35820 (STM resistant) and ATCC35837 (EMB resistant) standard bacteria strains. All the compounds except (48) and (52) showed intermediate anti-tubercular activity having MIC values less than 64 μg mL^−1^. Results suggested that the compound having a methyl group exhibited the best antimicrobial activity. Docking study revealed the van der Waal's interactions between S and Cl atoms of the ligand with amino acids LEU-269, ARG-225, PRO-227, LEU-269, ILE-228 and ALA-226 active, residues of the protein (Fig. 8).^71^

#### 6.1.4 Antiviral activity

**Fig. 44:**
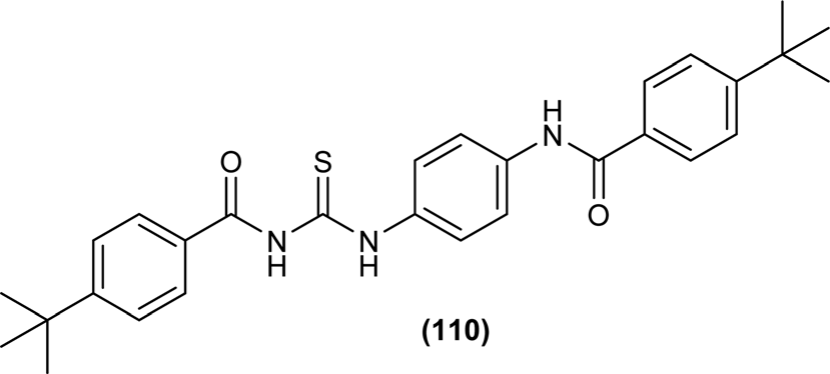
Structure of 4-(*tert*-butyl)-*N*-((4-(4-(*tert*-butyl)benzamido)phenyl)carbamothioyl) benzamide.

In the search of a new potent drug for enterovirus infections, Liu *et al.* found that 4-(*tert*-butyl)-*N*-((4-(4-(*tert*-butyl)benzamido)phenyl)carbamothioyl)benzamide (110) display prominent inhibitory activity against EV-A71, EV-D68, CV-A21, CV-A16 and CV-B1 and inhibiting proliferation of enterovirus based on intermolecular interaction like H-bonding, hydrophobic interaction and π–π stacking (Fig. 44). Mentioned acyl thiourea was also found effective in rats and reduced the mortality rate from 100% to 20% as well as provided physical relief from severe symptoms. It was concluded both *in vitro* and *in vivo* that the compound (110) is less toxic and more potent antiviral than standard drug, which revealed its potential in pandemic situations for enterovirus infections.^109^ Further, a series of acyl thiourea was synthesized as non-nucleoside polymerase inhibitors of influenza virus by Liu *et al.* Interestingly the same compound (110) was found as a more potent inhibitor among the synthesized compounds with EC_50_ value 0.8 nM against H1N1 proliferation and also showed activity against other influenza variants such as H1N1 variant (H1N1, H274Y) and *Influenza B virus*. The compounds inhibited the proliferation of targeted viruses by inhibiting the activity of RNA-dependent RNA polymerase.^110^

**Fig. 45:**
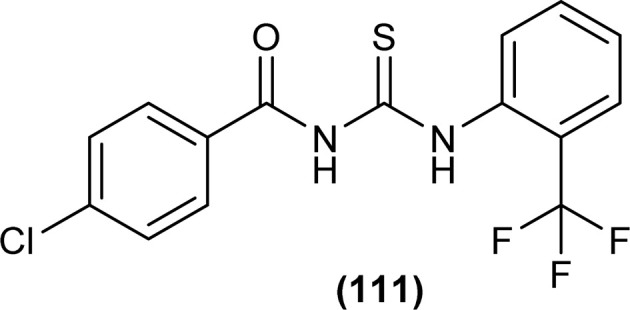
1-(4-Chloro-benzoyl)-3-(2-trifluoromethyl-phenyl)thiourea.

1-(4-Chloro-benzoyl)-3-(2-trifluoromethyl-phenyl)thiourea (111) synthesized by Alizada and Arslan and investigated against 6LU7 protein by molecular docking studies (Fig. 45). It was found that the compound showed H-bond interactions with the active site of (Glu166, Leu141, Cys145 and His164) of the 6LU7 protein, and the binding energy value was −6.61 kcal mol^−1^, it was concluded that the compound showed COVID-19 inhibition activity and can be used as a therapeutic candidate for corona diseases.^64^

**Fig. 46:**
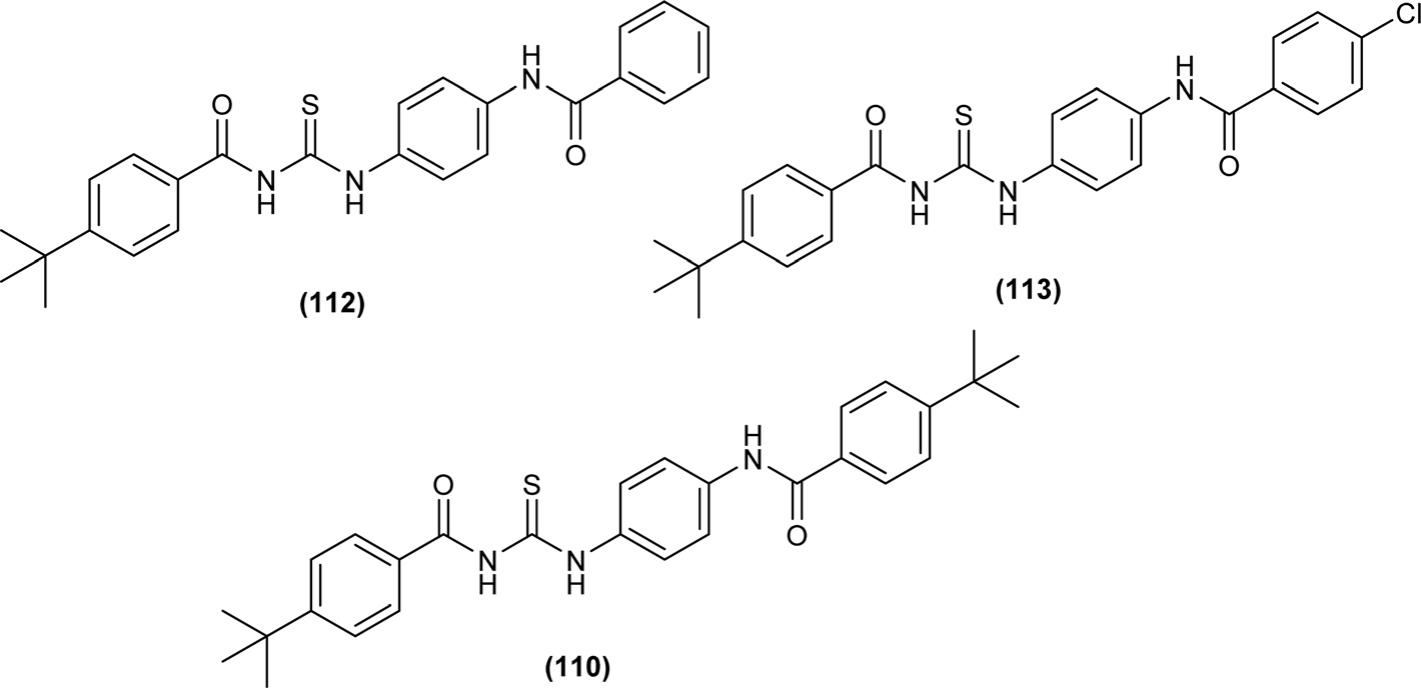
*N*-Phenylbenzamide based acyl thioureas.

Liu *et al.* synthesized a series of acyl thiourea that showed antiviral activity against influenza A and B subtypes. The antibacterial activity of the synthesized acyl thiourea was also confirmed by SAR studies and it was found that these compounds showed good *in vitro* activity. Acyl thiourea derivatives were evaluated for their antiviral activity against *Influenza B viruses* and oseltamivir-resistant subtypes (H1N1, H274Y) in MDCK cells. All the compounds showed good antiviral activity with EC_50_ values in the range of 0.05–4.0 or 6.2–0.04 μM against *Influenza B viruses* Yamagata and Victoria, respectively. Compound (110) was the most potent antiviral agent in the series. Compounds were investigated against oseltamivir-resistant strains (H1N1, H274Y) for their antiviral activity. It was found that the compounds (112), (113) and (110) showed good to excellent activities against H1N1 and H274Y strains with EC_50_ between 0.02 and 0.6 μM. From the SAR study of *Influenza B viruses* and H1N1, compound (110) was found potent against oseltamivir-resistant strain. From metabolic studies, compound (110) was found more stable in mice, dogs, human liver microsomes and human plasma (Fig. 46). The mode of action of these acyl thioureas was explained based on molecule targeting the RNA-dependent RNA polymerase to inhibit the proliferation of influenza viruses. The *t*-butyl group on the aromatic ring and thiourea functionality in the synthesized compounds were the reasons behind the antiviral activity of these compounds.^110^

**Fig. 47:**
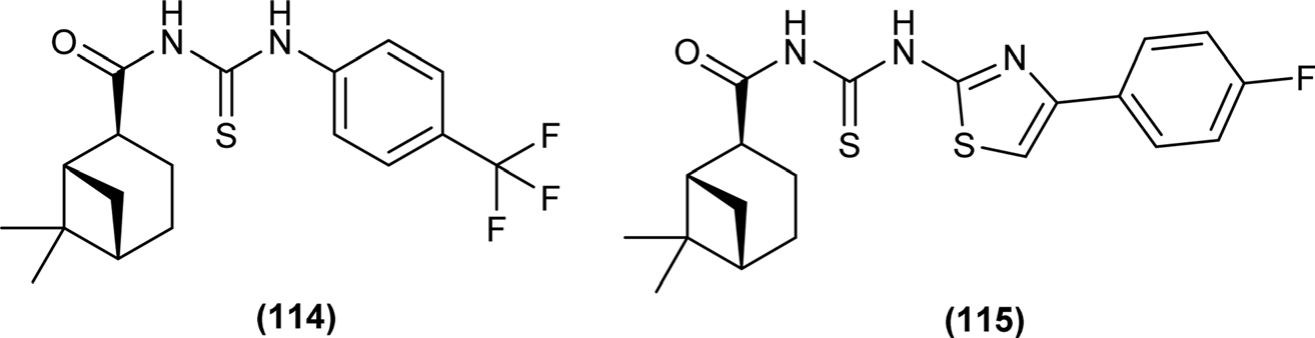
Myrtanyl-based acyl thioureas.

Myrtanyl acyl thiourea derivatives were synthesized and evaluated for their antiviral activity against the influenza virus A/Puerto Rico/8/34 (H1N1) strain. Among the series compounds (114) and (115) showed weak antiviral activity against virus A (H1N1) at a concentration of 100 μmol L^−1^, with maximum inhibitions of 34.8% and 16.1%, respectively. Above mentioned compounds showed antiviral activity, because they contained fluorine atoms and thiazole structures (Fig. 47).^111^

#### 6.1.5 Antiparasitic activity

Antiparasitic activity of the complexes synthesized by De Oliveira *et al.* against amastigote and trypomastigote forms of *T. cruzi* were evaluated and the results show that the compounds are better antiparasitic with better IC_50_ values compared to benznidazole and were more selective toward trypomastigote. Variation of metal ions strongly influences the antiparasitic activity of complexes and thus complexes of platinum (65a) and (65b) show better activity against trypomastigote and less active against amastigote compared to the palladium and nickel complexes (Fig. 13).^79^

#### 6.1.6 Analgesic activity

**Fig. 48:**
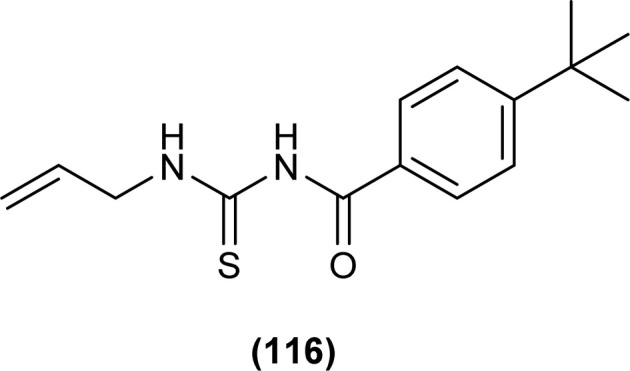
1-Allyl-3-(4-tertiary-butylbenzoyl)thiourea.

Novel 1-allyl-3-(4-tertiary-butylbenzoyl)thiourea (116) was synthesized and investigated for analgesic activity by Razak *et al.* The synthesized compound showed better interaction with pain receptors and was found more potent analgesic agent than Diclofenac sodium. The mean percentage inhibition of pain was 53.29% at a dosage of 25 mg kg^−1^ BW (Fig. 48).^112^

#### 6.1.7 Antidiabetic and antioxidant activity

**Fig. 49:**
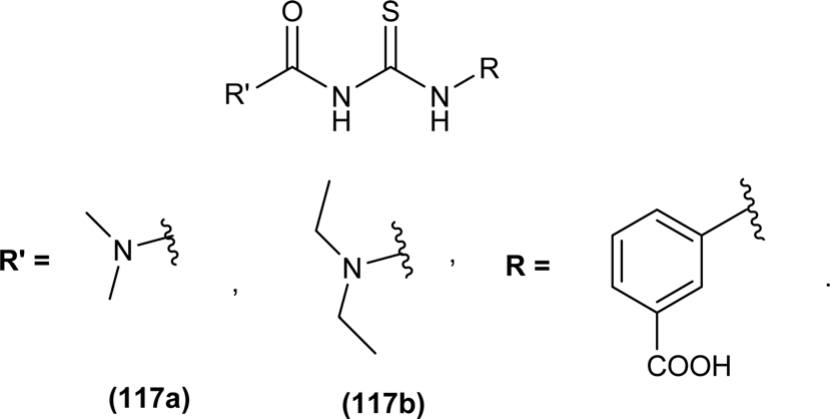
Dimethyl and diethylcarbamoyl-*N*,*N*′-disubstituted based acyl thioureas.

Khan *et al.* synthesized four novel acyl thiourea derivatives and their molecular docking analysis were carried out against α-amylase and α-glucosidase, compound 3-(3-(dimethylcarbamoyl)thioureido)benzoic acid (117a) showed both hydrophobic interactions and formed hydrogen bonds with amino acids HIS A:103, ASP A:326, ARG A:407, ARG A:411 of pancreatic α-amylase. Compound (117a) (IC_50_ = 19.26 ± 0.23 μM) and 3-(3-(diethylcarbamoyl)thioureido)benzoic acid (117b) (IC_50_ = 21.89 ± 0.06 μM) were found active against α-glucosidase and α-amylase with comparable activity as standard drug acarbose. The compound (117a) showed both antidiabetic and anti-oxidant activity but negligible hemolytic activity. High activity of the compound (117a) and low binding affinity of (117b) toward various amino acids of the respective enzymes were also revealed by molecular docking studies. Compound (117a) existed in monoclinic crystal form. Prominent interactions among synthesized molecules and active sites of enzymes were π–π stacking, π-alkyl, and van der Waal's interactions (Fig. 49).^113^

It was found that the compound *N*,*N*-di-2,4-dimethoxybenzyl-*N*′-2-nitrobenzoylthiourea (88) showed good radical scavenging and antioxidant activity which is due to electron or hydrogen atoms donating ability of the title compounds toward free radicals (Fig. 29).^62^

Acyl thiourea ligands synthesized by Shah *et al.* showed a good DPPH radical scavenging effect. Co(ii) complexes showed more antioxidant activity than thiourea complexes of Cu(ii) metal. Among cobalt complexes compound TH05-Co(ii) had good antioxidant potential with an IC_50_ value of 11.4 ± 0.2 μg mL^−1^ comparable to standard ascorbic acid (IC_50_ 10.8 ± 0.5 μg mL^−1^). Transfer of electrons from thiocarbonyl group C=S and a hydrogen atom from NH efficiently stabilized two radicals of DPPH. Low activity of some thioureas was due to steric hindrance with DPPH which makes NH unavailable for DPPH radical attack (Fig. 11).^76^

Antioxidant activity of the Pd complexes was investigated against DPPH by Dorairaj *et al.* with ascorbic acid as reference. Different concentration of the complexes was used which revealed that the ability to scavenge the DPPH radical increased as the concentration of metal complexes was increased and occasionally showed better activity than standard ascorbic acid. Complex (62d) had a scavenging ability of up to 86.19%. Antioxidant activity order of different complexes was 62d > 62a > 62c > 62b > 62e (Fig. 12).^78^

Antioxidant activity of ligands (71a–c) and corresponding *cis*-Pt complexes (71a–c) was investigated by Nkabyo *et al. via* ORAC, DPPH and FRAP assays. Generally, the acyl thiourea ligands showed better antioxidant activity to scavenge oxygen and free radicals generated by Fe^3+^ than their corresponding metal Pt(ii) complexes except *cis*-Pt 71c complex which showed high ORAC and FRAP values than the other two complexes when used at high concentration. The antioxidant activity of the acyl thiourea ligand was strictly dose-dependent. Amongst chelating ligands (71c) showed higher activity than (71a-b) in FRAP protocols due to the electronic effects of substituent on acyl thioureas (Fig. 16).^82^

The total antioxidant activity of the acyl thioureas (95a–g) synthesized by Roman *et al.* was evaluated using DPPH assay. All compounds (95a–g) exhibited antioxidant activity, TAC for compounds 95f (∼25%) and 95d (∼43%) was highest while all remaining compounds showed total antioxidant activity between 10 and 15% (Fig. 33).^97^

Anti-oxidant activity of the compound (98) and its Cu(ii) complex (99) suggested that the ligand (MBT) exhibited higher activity than the Cu(ii) complex and had the highest percentage inhibition of 36.41% at a concentration of 22.5 μg mL^−1^ (Fig. 35).^99^

**Fig. 50:**
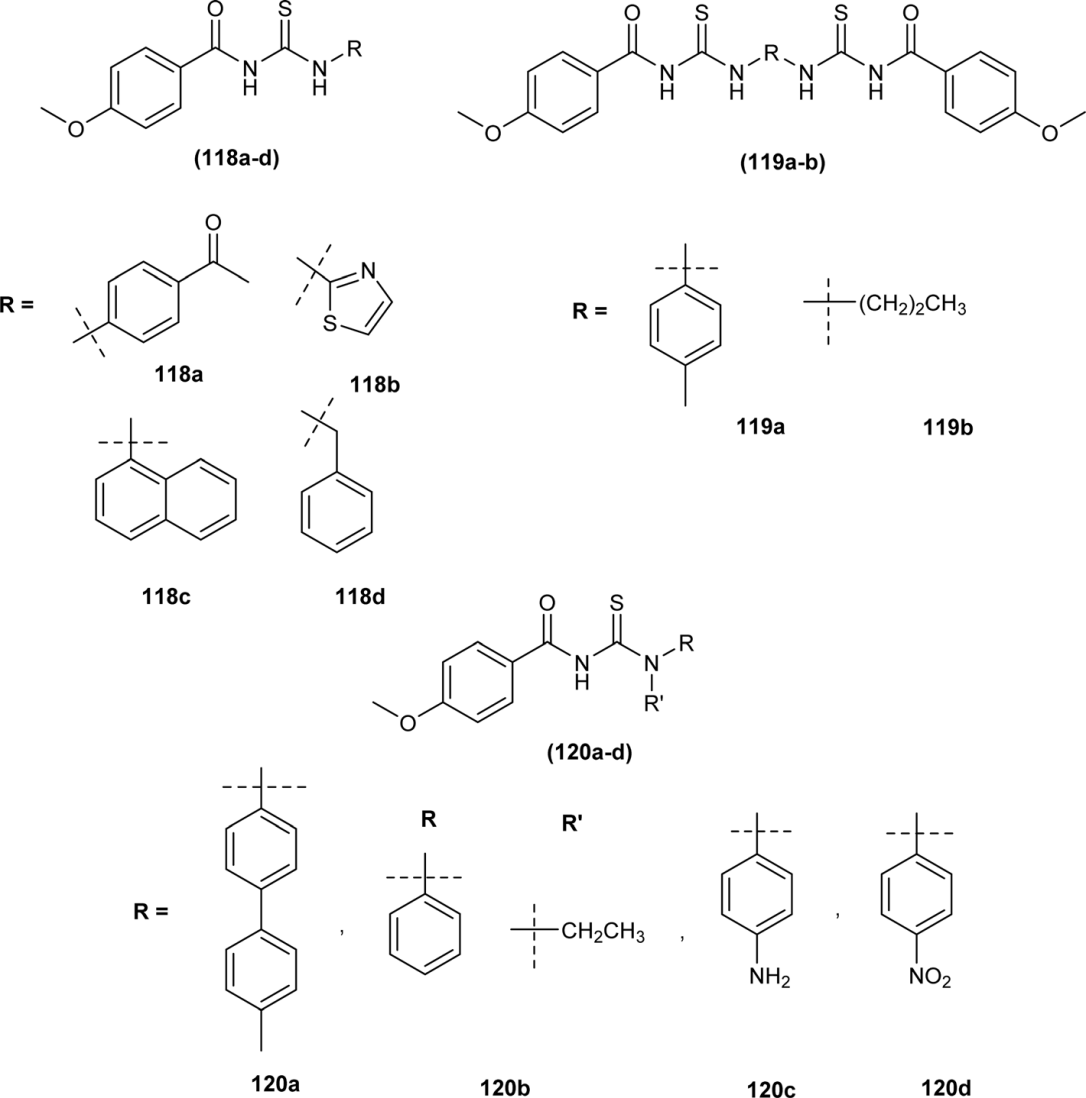
Acyl thiourea derivatives of 4-methoxybenzoyl chloride.

Novel acyl thiourea derivatives of 4-methoxybenzoyl chloride were synthesized and evaluated for antioxidant activity by Oleiwi *et al.* Free radical scavenging method using DPPH was used for evaluation of activity. In series compound (120b) containing tertiary amine group (IC_50_ 5.8 μg mL^−1^) showed good antioxidant activity followed by compound (119b) (IC_50_ 42.3 μg mL^−1^) and (118c) (IC_50_ 45 μg mL^−1^) due to presence of acyl thiourea moiety and naphthyl group, respectively. From docking studies of compounds (118a–d, 119a-b, and 120a–d) it was found that acyl thiourea having bulky groups and more than one thiourea units exhibited better inhibition activity against urease protein (4ubp). Compound (118a) showed the best docking interaction with the lowest binding energy followed by compounds (119a) and (119b). Compounds (120a) and (119a) showed hydrogen bonding interaction with Gly368 and Arg369 and hydrophobic affinity toward Leu365 and Arg369, these four interactions were responsible for the best docking scores (Fig. 50).^114^

**Fig. 51:**
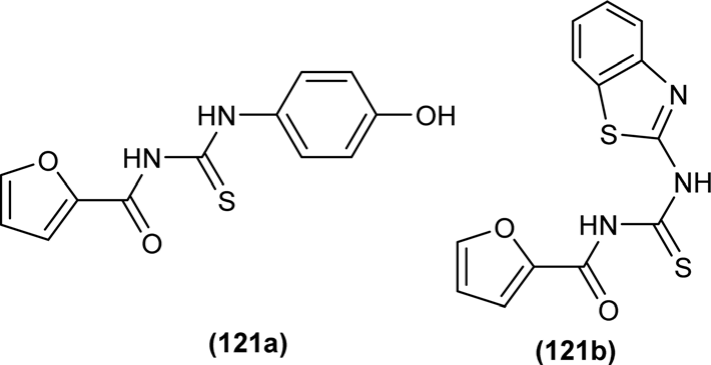
1-(2-Furoyl)thiourea derivatives.

A series of thirteen novels 1-(2-furoyl)thiourea derivatives were synthesized, characterized by various spectroscopic techniques and investigated for anti-oxidant activity by Al-Jeilawi *et al.* The activity of thiourea derivatives turned out to be dose-dependent. Compound (121a) followed by compound (121b) showed best antioxidant activity at the lowest concentration of 25 ppm having IC_50_ values of 29.5 μg mL^−1^ and 32.08 μg mL^−1^, respectively. The antioxidant activity of the compound (121a) was due to the presence of the hydroxyl group while that of compound (121b) was attributed to benzothiazole (Fig. 51).^115^

Anti-oxidant activity of a novel series of acyl thiourea derivatives (102a–o) was determined using the DPPH assay method. Amongst the series compound (102i) followed by compound (102a) showed the highest antioxidant capacity of 87% and 44%, respectively, while all remaining compounds showed antioxidant capacity between 0 and 29% (Fig. 38).^102^

Triazole (36) synthesized from pivaloyl thiourea showed good antioxidant activity and the minimum concentration required was 0.45 μg mL^−1^ (Scheme 10).^54^

**Fig. 52:**
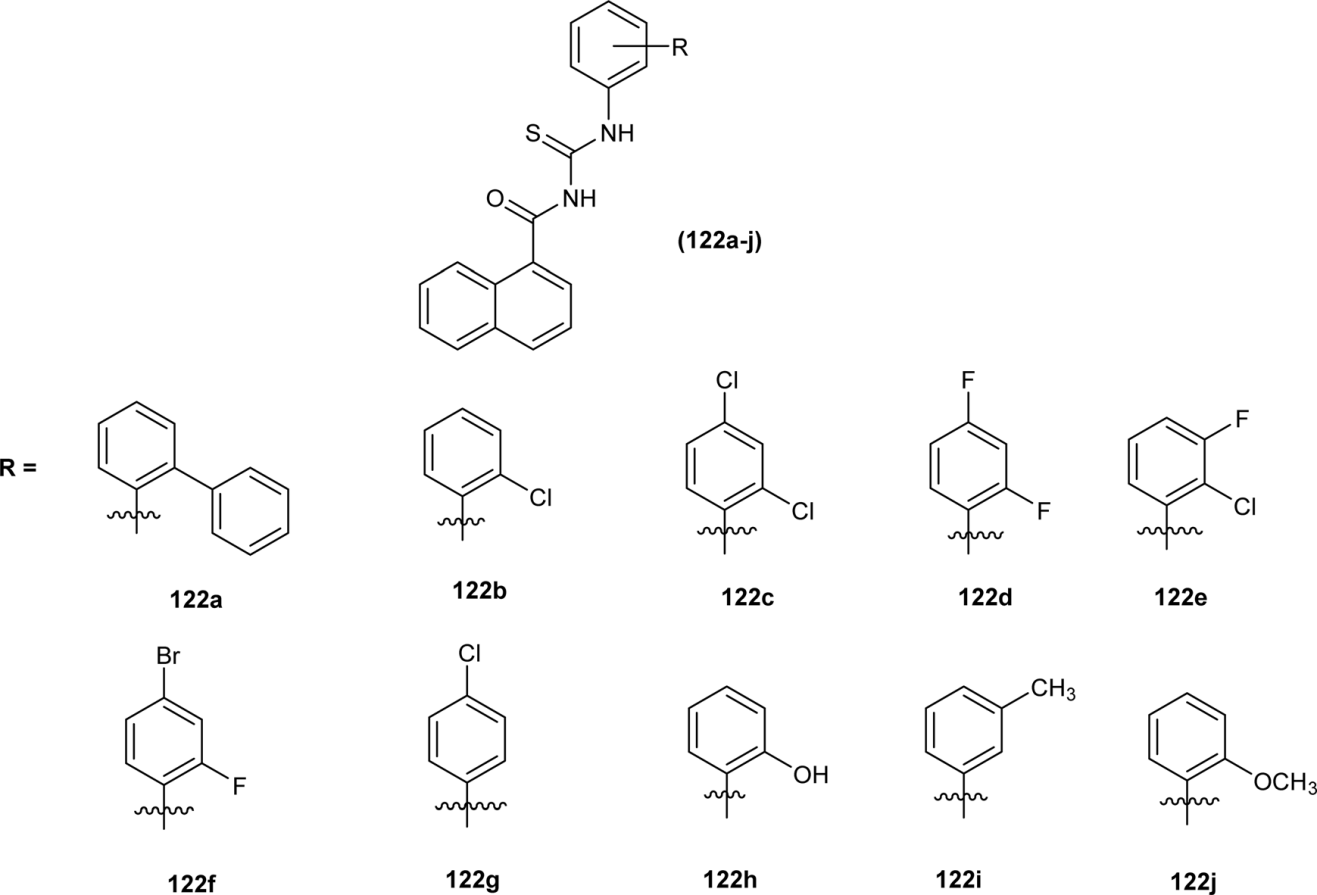
Naphthalene-based acyl thiourea conjugates.

Amongst the series of naphthalene-based acyl thiourea conjugates (122a–j), compounds (122i and 122h) were found most effective free radical scavengers. The highest free radical scavenging capacity of compounds (122i) and (122h) were due to the presence of electron-donating –CH_3_ and –OH group at -*meta* and -*ortho* positions of phenyl moiety (Fig. 52).^116^

#### 6.1.8 Anticancer activity

**Fig. 53:**
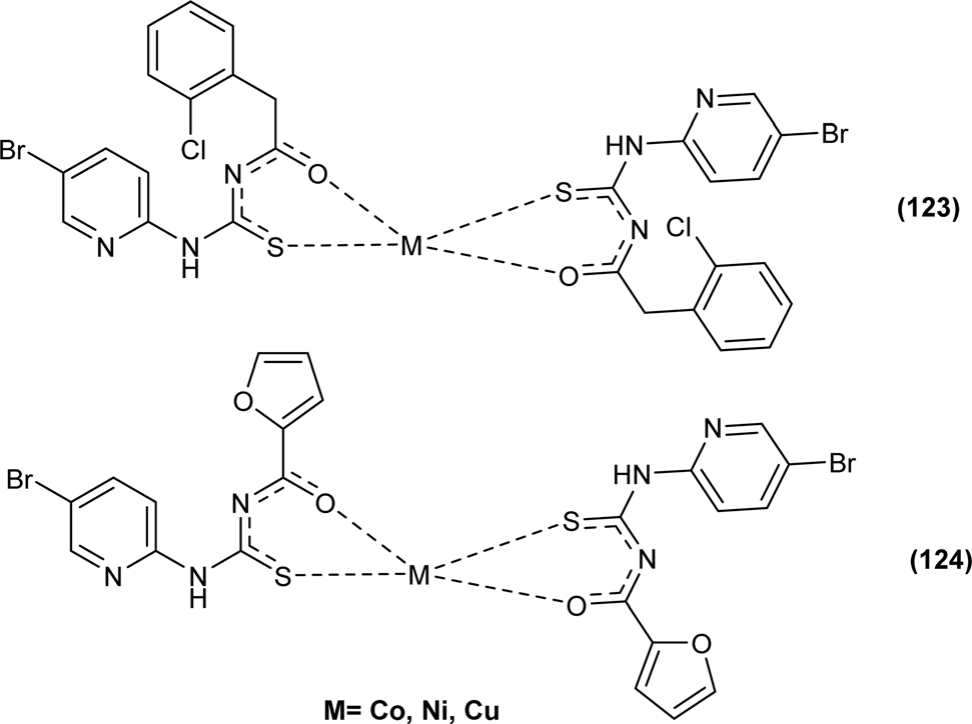
Co, Ni, and Cu complexes containing acyl thiourea ligands.

Anticancer activity of the synthesized complexes (123) and (124) by Yeşilkaynak *et al.* was investigated against MCF-7 breast cancer cells. The IC_50_ values for the synthesized complexes were in the range from 2.07 μM to 21.25 μM against MCF-7 cells. The (123) complex of Ni was the most potent of all synthesized complexes. Complex (123) was more effective than complex (124) which was attributed to the presence of Cl substituent on the phenyl ring (Fig. 53). Good inhibitory activity against BRAF (V600E) protein kinase, another target for the study of anticancer activity, was also proposed *via* molecular docking studies.^75^

**Fig. 54:**
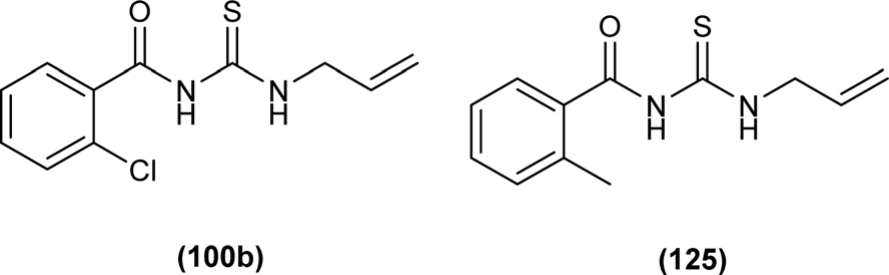
Allyl-based acyl thioureas.

*N*-(Allylcarbamothioyl)-2-chlorobenzamide (100b) and *N*-(allylcarbamothioyl)-2-methylbenzamide (125) synthesized by Yeşilkaynak *et al.* were investigated against MCF-7 human breast cancer cells. The highest inhibition activity was observed when used at the concentration level of 100 μmol L^−1^. Inhibition activity of (100b) and (125) was high when used at low concentrations of 6 and 25 μmol L^−1^, respectively for 24 h. When the exposure time of breast cancer cells was increased up to 48 h then the concentration of acyl thiourea for anticancer activity reduced to IC_50_ values 2.59 and 7.09 μmol L^−1^, respectively. It was concluded that the anticancer activity of these two compounds was found to be time and dose-dependent. Overall (100b) exhibited the best anticancer activity with an IC_50_ value of 2.59 μmol L^−1^ at 48 h while (125) showed good cytotoxic activity at 24 h with an IC_50_ value of 3.99 μmol L^−1^. From molecular docking studies, it was found that the synthesized compounds also showed binding interaction and inhibitory activity against BRAF (V600E) protein kinase (Fig. 54).^65^

Metal complexes of Ni, Pt, and Pd with acyl thiourea as ligand were synthesized by De Oliveira *et al.* and were evaluated for their cytotoxic activity against tumor cell lines (MDA-MB-231 and MCF-7) and non-tumor cell line (MCF-10A), the complexes showed lower IC_50_ values against MDA-MB-231 and MCF-7 than MCF-10A. Changing the *R*_1_ substituent in the acyl thiourea part of complexes did not show considerable effect on the MCF-7 and MCF-10A lines while these variations efficiently influenced the TNBC cell line (MDA-MB-231). Metal variation in complexes showed a greater effect on cytotoxic activity, especially on the MDA-MB-231 line. Ni(ii) complexes (63a-b) showed lower IC_50_ values than Pt(ii) (64a-b) and Pd(ii) complexes (65a-b). In breast cancer cell line MCF-7, Pt(ii) and Pd(ii) complexes showed similar IC_50_ values to Ni(ii) complexes. Variation in phosphine ligands also influenced the activity of metal complexes, PPh_3_ ligands showed higher activity than dppe ligand-containing complexes (Fig. 13).^79^

**Fig. 55:**
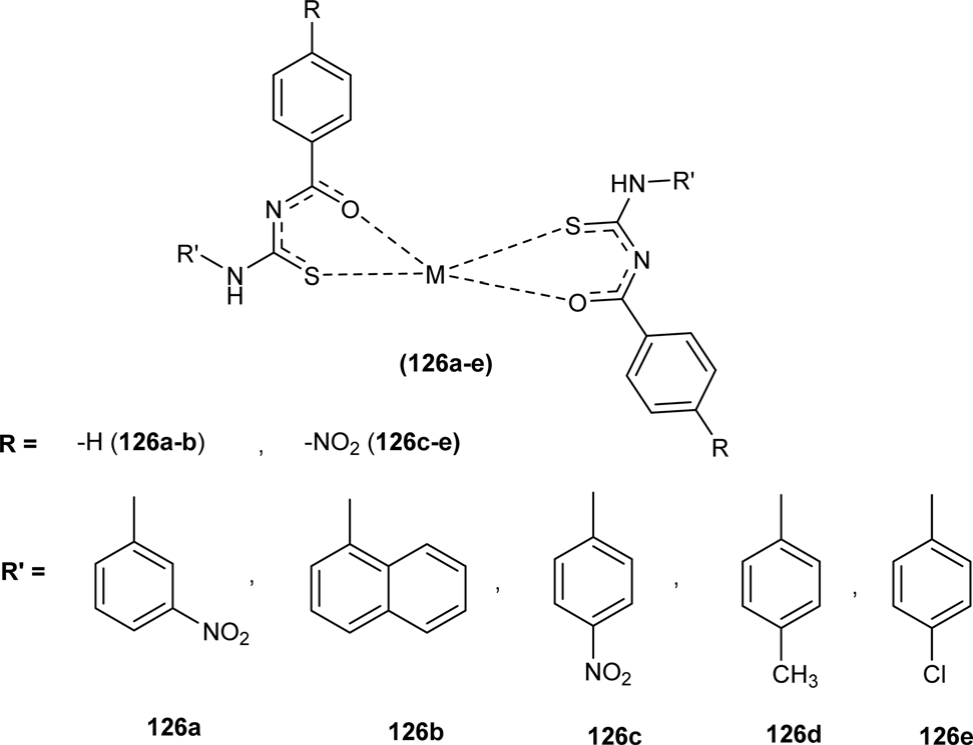
Cu(ii) complexes containing acyl thiourea ligands.

Al-Salim and Al-Asadi synthesized acyl thiourea derivatives and their Cu(ii) complexes (126a–e), molecular docking studies and the activity of synthesized compounds as anticancer agents against breast cancer was reported (Fig. 55). Amongst the acyl thiourea ligands, complexes (126a and 126b) were found more effective with IC_50_ values of 92.09 μg mL^−1^ and 66.07 μg mL^−1^ respectively than other ligands. The Cu(ii) complexes of these ligands showed better inhibiting activity compared to parent ligands (IC_50_ = 4.03 and 4.66 μg mL^−1^) due to the high penetration ability of complexes through the cancer cell membrane.^117^

Bipodal acyl thiourea ligands and their Ru(ii) complexes (72a–c and 73a–c) were evaluated against lung (A549), breast (MDA-MB-231 and MCF-7), and cervical (HeLa and MCF-10a) cancer cell lines. All derivatives showed negligible activity except L1 and L3 which exhibited moderate anticancer activity with IC_50_ = 83–90 μM against few cancer cells. Ru(ii) complexes of the synthesized ligands showed the highest anticancer activity against triple-negative breast MDA-MB-231 cancer cells and also showed more selectivity toward cancer cell lines. Anticancer activity of the synthesized complexes was due to the formation of adducts with biomolecules, the substituent position on aryl moiety in thiourea ligands caused variable effect on activity. *Ortho*-substituted showed low interaction as compared to *para*-substituted with active sites of biomolecules. Similarly, the enhanced activity of benzene complexes of Ru as compared to *p*-cymene, was due to the presence of methyl and isopropyl groups on *p*-cymene which hindered the adduct formation (Fig. 17).^83^

Ru(ii) complexes having furoyl thiourea ligands were investigated against breast cancer cells MCF-7, MDA-MB-231, and T47-D by Dorairaj *et al.* Both the acyl thiourea ligands and their complexes (74a–l) showed dose-dependent anticancer activity against breast cancer cell lines. Furoyl thiourea ligands showed IC_50_ values ranging between 27.65 and 40.82 μM against MCF-7, MDA-MB-231, and T47-D cancer cells. Comparatively, complexes showed greater activity than ligands, thus highlighting the importance of Ru ion and arene moiety. Complexes (74a–f) had two chloride ligands whereas complexes (74g–l) had one chloride ion and one PPh_3_, later one exhibited better activity against MCF-7, MDA-MB-231, and T47-D with IC_50_ values 0.62–3.98, 0.15–2.81, and 0.17–2.88 μM, respectively. It was concluded that the presence of lipophilic moiety PPh_3_ and electron-donating groups like methyl and methoxy increased the activity of complexes. Further *in vivo* study of complexes in mice show that these complexes at high dosages up to 8 mg kg^−1^ did not damage any organs (Fig. 18).^84^

Acyl thiourea complexes of Ru(ii) (75a–f) were synthesized and evaluated against Cisplatin-resistant lung carcinoma (cisA549R), human lung carcinoma (A549) and normal human umbilical vein endothelial cell (HUVEC), these complexes showed comparable activity Cisplatin and overcame the resistance offered by cisA549R cell line toward reference. Complexes (75d and 75e) showed better anticancer activity with IC_50_ values of 8.74 and 5.37 μM against (A549) and 10.76 and 21.36 μM against (cis RA549) cell lines, respectively. SAR study suggested that increasing the chain length or aromatic conjugation increased the cytotoxicity of complexes which was associated with the enhanced lipophilicity of complexes and thus increased the uptake of complexes irrespective of type of cells *i.e.* normal or cancer cells (Fig. 19).^86^

**Fig. 56:**
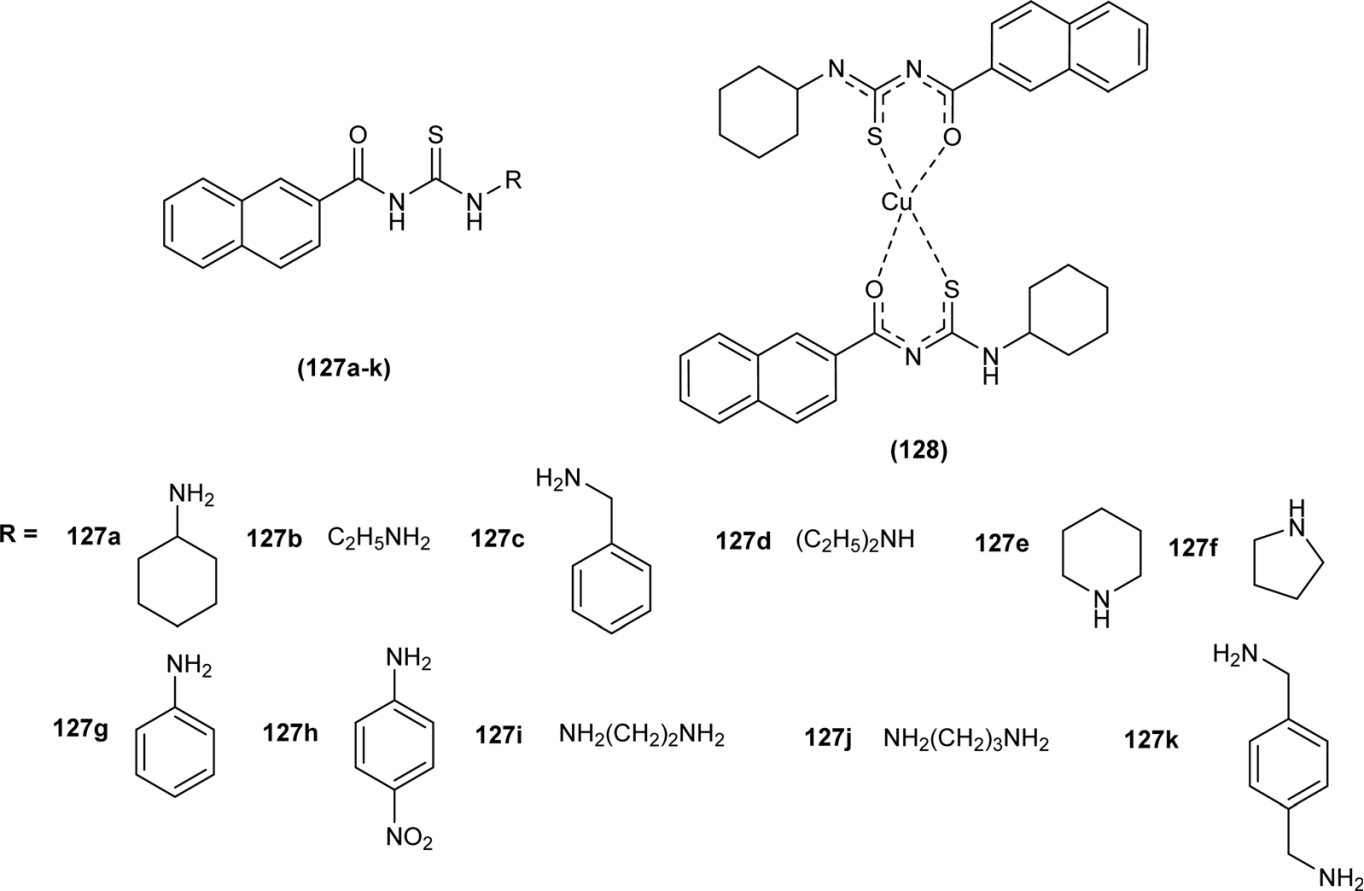
Naphthoyl thiourea derivatives and its Cu(ii) complex 128.

Novel naphthoyl thiourea derivatives were synthesized and evaluated for anticancer activity against human cancer cell lines HCT-116, MCF-7 and A549 in addition to normal cancer cell line MCF-10A. Derivatives (127a–h) showed good antiproliferative activity while compounds (127i–k) showed prominent activity against the above three human cancer cell lines with IC_50_ < 2.9 μM. Cu(ii) complex (128) of the naphthoyl thiourea derivative had enhanced antiproliferative activity than acyl thiourea ligands. Compounds with high anticancer activities (127i–k) had less cytotoxicity toward normal human cells with high IC_50_ values ranging between 76.46 and 91.38 μM. Compounds (127a–h) exhibited anticancer activity due to the presence of polar thiocarbonyl group which causes interaction with hydrophilic active sites of biomolecules, and the cytotoxicity of these compounds against normal cells was because of increased lipophilicity due to the presence of electron-donating alkyl groups. Bis-substituted acyl thiourea (127i–k) showed better anticancer activity than monosubstituted thiourea (127a–h), this enhanced activity was due to extra naphthoyl moiety in compounds (127i–k) (Fig. 56).^48^

Pd(ii) complexes of acyl thiourea (76a–d) synthesized by Dorairaj *et al.* were evaluated against HeLa, HCT116, HepG2 (cancer), and HEK293 (normal) cell lines. Complexes were found more potent than acyl thiourea ligands and exhibited considerable activity against HeLa but showed excellent activity against HCT116 and HepG2. Compound C2 was found more potent with an IC_50_ value of 6.5 μM (HeLa) than standard Cisplatin. These complexes were selective toward cancer cells and did not affect normal cancer cell lines. The highest activity of compound (76b) was due to the presence of a stable C–Cl bond, which interacts with biomolecules and the presence of σ-hole interactions in complexes with biomolecules (Fig. 20).^87^

Anticancer activity of the synthesized Cu(i) complexes (79a–e) of acyl thiourea was evaluated against breast cancer cells MCF7, T47D, and MDA MB 231 by Dorairaj *et al.* Complexes showed dose-dependent activity with the best results observed as, IC_50_ 0.75–0.98 ± 0.01 μM against MCF7 cancer cells, 0.75–0.94 ± 0.02 μM against T47D cancer cells and 0.68–0.95 ± 0.02 μM against MDA MB 231 cancer cells, when exposed to different concentration of complexes for 72 h. These results were better compared to the standard drug Cisplatin. Amongst the complexes (79d) showed the highest activity due to strong interaction with biomolecules, similarly complexes (79b–e) showed high activity as compared to (79a) because of its lipophilic nature which allowed its penetration across the membrane of cells, and the second reason was the presence of substituent on aromatic moiety attached to thioamide N (Fig. 22).^89^ Binuclear Cu(i) complex (81) of *N*-(2-thiophenecarbonyl)-*N*′-(3-Cl,4-F-phenyl)thiourea also exhibit anticancer activity against human lung cancer cell line (A549) with IC_50_ value 10.9 ± 1.42 μM (Fig. 24).^91^

Co(ii), Ni(ii), and Cu(ii) metal complexes of *N*-(1,10-biphenyl)-2-chlorobenzoylthiourea (82) were investigated against MCF-7 breast cancer cells and also evaluated for antioxidant activity. All the complexes were found effective against MCF-7 cells having IC_50_ values in the range of 24.3–49.2 μM. Amongst the complexes, the Cu(ii) complex possessed strong antitumor and antioxidant activity, but these complexes were not very effective compared to 5-fluorouracil, IC_50_ = 4.7 μM (Fig. 25).^92^

*N*-(2,4-Dichloro)benzoyl-*N*′-phenylthiourea (7) exhibited prominent anticancer activity against MCF-7 and T47D cancer cell lines with IC_50_ values in the range 0.31 ± 0.05 and 0.94 ± 0.02 mM, respectively as compared to hydroxyurea and showed selective activity towards cancer cells than normal Vero cell line (Scheme 2).^42^

**Fig. 57:**
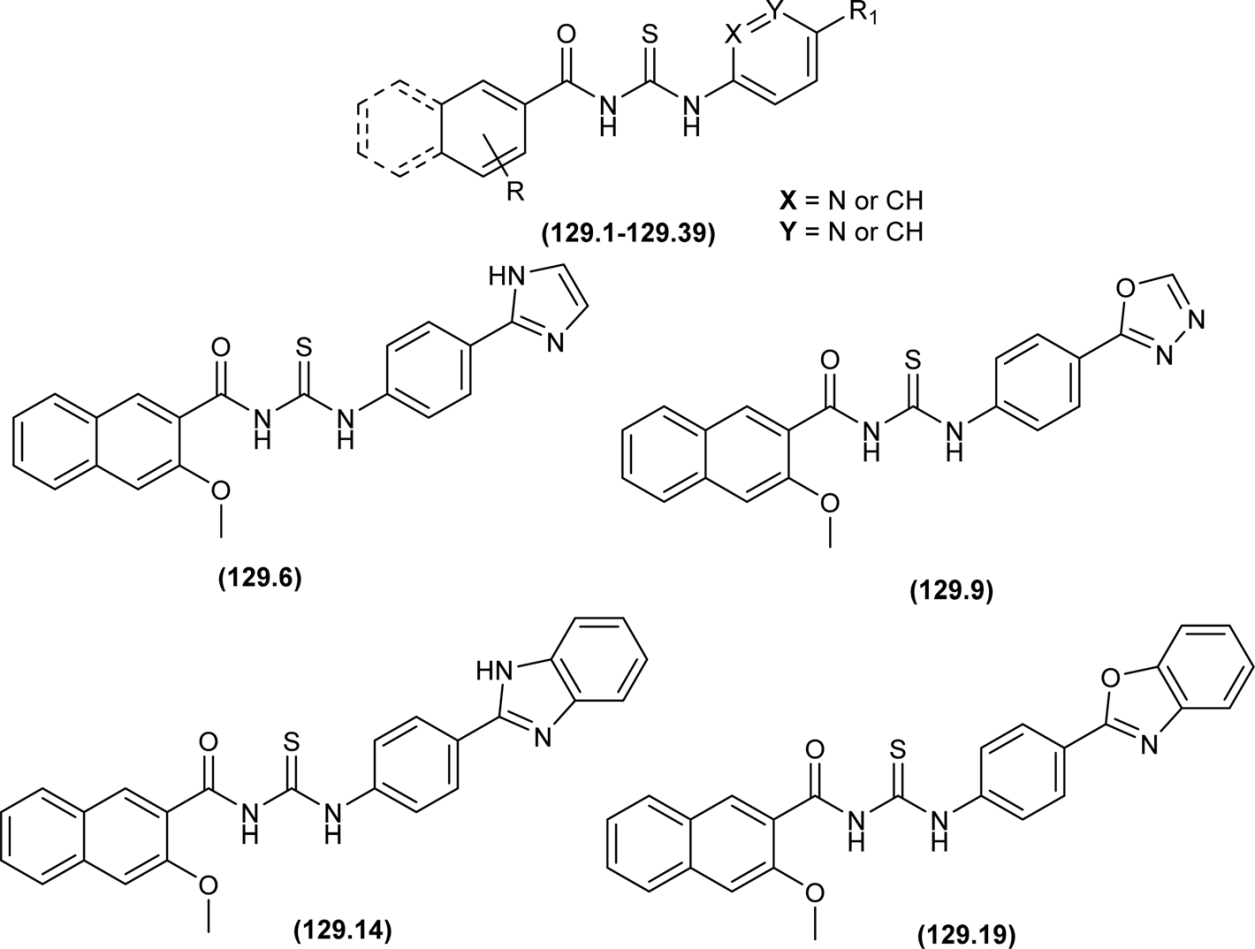
*N*-(Phenylcarbamothioyl)-2-napthamides.

A novel series of *N*-(phenylcarbamothioyl)-2-napthamides (129.1–129.39) were synthesized as Claudin-1 inhibitors by Mashinson *et al.* All synthesized compounds were investigated against colorectal cancer cells SW620. Most of the heterocyclic analogs of acyl thiourea were found inactive. However, compounds (129.6, 129.9, 129.14 and 129.19) showed eminent anticancer activity against SW620 colorectal cancer cells with % inhibition values at conc. of 25 μM being 89.9, 86.5, 90.8 and 76.4, respectively (Fig. 57).^118^

Novel furoic acid-based acyl thiourea derivatives (101a–f) were evaluated against HepG2, Huh-7, and MCF-7 cancer cell lines. Compound (101d) showed the best anticancer activity with fewer cell viabilities (33.29, 45.09, and 41.81%, respectively). Compounds (101a–c) also showed prominent activity against HepG2 having cell viability of 35.01%, 37.31%, and 39.22%, respectively, while compounds (101e and 101f) were found least active against all tested cancer cell lines having high cell viability (63.75–82.81%) (Fig. 37).^101^

**Fig. 58:**
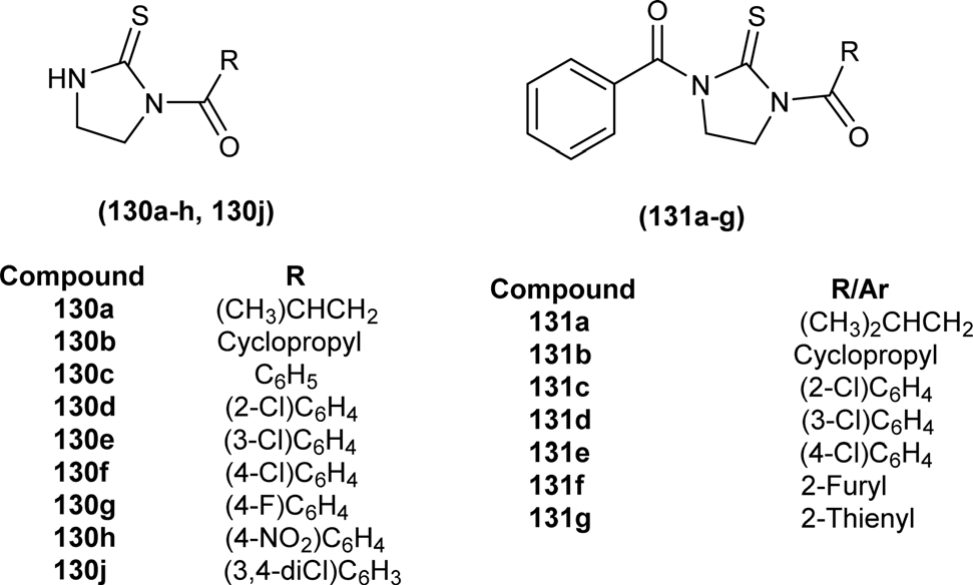
Mono and di-acylated imidazolidine-2-thione derivatives.

Novel mono-acylated (130a–h, 130j) and di-acylated thiourea derivatives (131a–g) were synthesized and investigated against SKOV-3 and MCF-7 cancer cell lines, but none of the derivatives exhibited cytotoxicity against SKOV-3 and MCF-7 cancer cell lines (Fig. 58). *In silico* study indicated good pharmacokinetic properties and drug-like characteristics for these derivatives.^119^

**Fig. 59:**
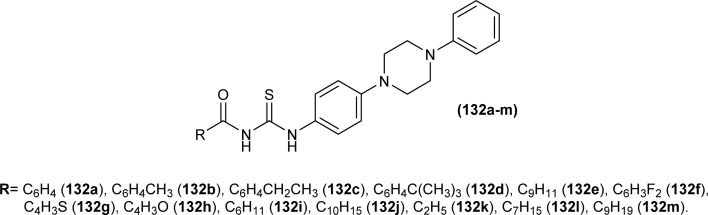
Piperazine containing thioureas.

A series of piperazine-containing thioureas were synthesized by Sashankh *et al.* for the treatment of colon and rectal cancer. Cytotoxic activity of all compounds was tested against colon cancer cells (HCT116, HCT116+ch3 and SW620). All compounds exhibited intercalation binding with CT DNA, from the series compound (132b) was found to be a more potent anticancer agent against HCT116+ch3 cells than Cisplatin and showed less cytotoxicity toward normal FHC cells. The high cytotoxic activity of the compound (132b) was due to the presence of planar phenyl rings and electron-donating methyl group present on the phenyl ring (Fig. 59).^61^

**Fig. 60:**
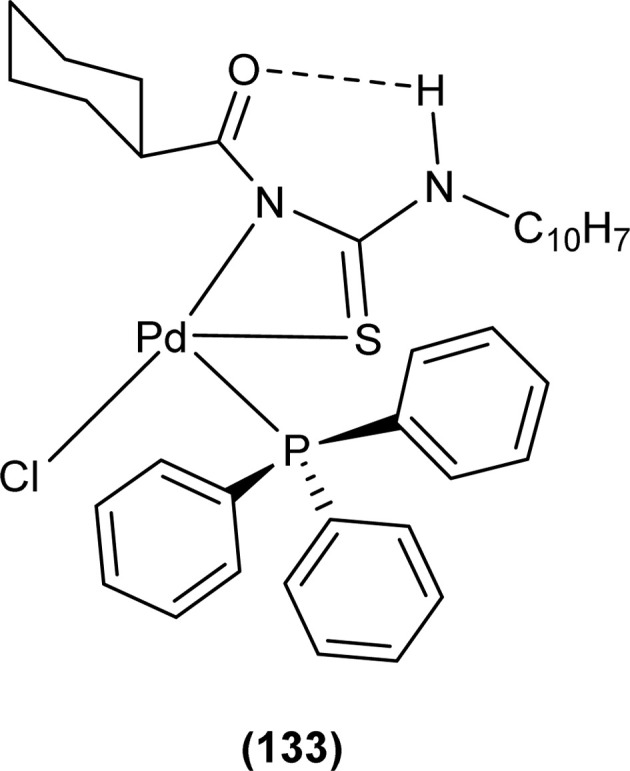
Palladium complex containing acyl thiourea ligand.

A series of Pd metal complexes synthesized by Dorairaj *et al.* were evaluated for their anticancer activity against MCF7 (breast), HeLa (cervical), A549 (lung) cancer HEK-293 (human embryonic kidney) normal cells. The activity of the compounds was found to a dose-dependent when incubated for 24 h, and IC_50_ values of the synthesized complexes were in the range of 8.6–12.4 μM against HeLa, MCF7 and A549 cells and selectively targeted cancer cells. Anticancer activity of the complexes was comparable to that of Cisplatin. Compound (133) was more potent than other complexes with IC_50_ values of 8.6 (HeLa), 8.8 (MCF7) and 9.4 (A549) μM. It was also found that the complexes showed better activity than the acyl thiourea ligands, the reason being their better binding ability (Fig. 60).^78^

Ligand (77) and metal complexes synthesized by Muhammed *et al.* were screened for anticancer activity against CEMSS leukemia cells. Two complexes [Pd(L)_2_(dppp)] and [(dppe)Pd(μ-L)_2_CoCl_2_(H2O)_2_] (78) showed moderated activity having IC_50_ range from 12.3 ± 0.75 to 25 ± 1.1 μM, when the cells were treated with it for 72 hours (Fig. 21).^88^

#### 6.1.9 Antihyperthyroidism activity

Effect of acyl thiourea ligand *N*-((3,5-dichloropyridin-2-yl)carbamothioyl)pivalamide (83) and its complexes with Zn(ii) metal LZnCl and (L)_2_ZnAC were investigated for antihyperthyroidism by Shams *et al.* All these compounds were found effective and showed a prominent decrease (*P* ≤ 0.05) in the level of T_3_ and T_4_ thyroid hormones after 20 days of using these compounds. Thus potential use of these compounds in the future to control hyperthyroidism was proposed (Fig. 26).^93^

#### 6.1.10 Anti-inflammatory activity

**Fig. 61:**
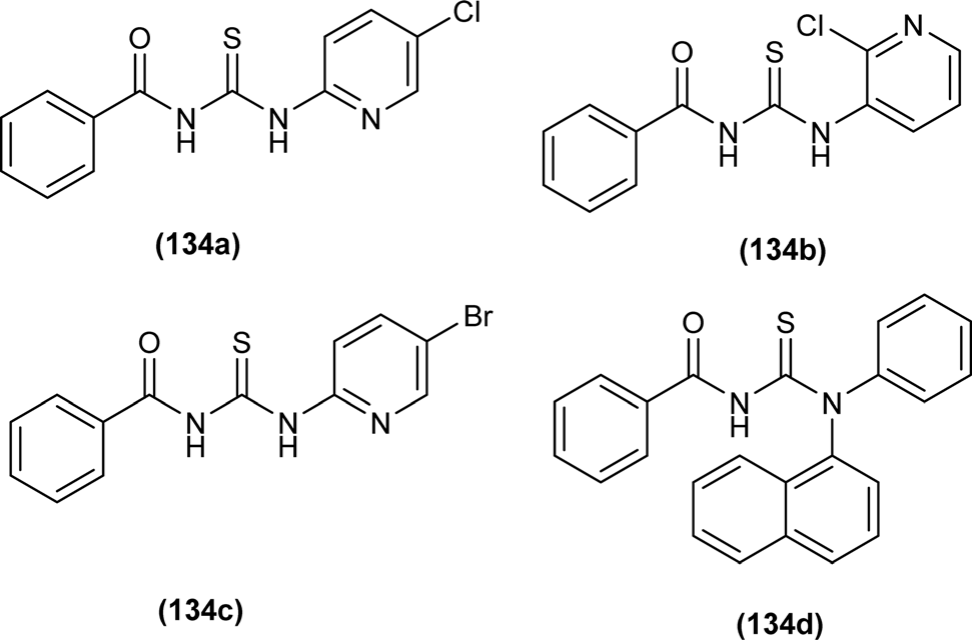
Structure of acyl thioureas 134a–d.

The anti-inflammatory effect of the synthesized compounds was determined by incubating them with and without LPS. It was found that all the acyl thioureas show no activity in the dark, macrophages were activated by LPS but there is no change in the production of TNF and IL6 levels. When the samples were illuminated for 5 min the results were still the same as in dark conditions, but after 10 min of illumination, compounds (134b and 134d) showed potent anti-inflammatory effects by decreasing the production of TNF (134b) significantly decreased the production of IL6 compared to (134d) after 10 min of illumination, thus indicating that (134b) was a potent photodynamic anti-inflammatory agent because it decreased the production of both TNF and IL6 after 10 min of illumination. Compound (134d) was effective only when used in high concentration and only decreased the production of TNF after 10 min of illumination, and all the remaining acyl thioureas were not effective both in dark and illuminated situations. The high photodynamic anti-inflammatory activity of the (134b) was due to the presence of electron-withdrawing group chlorine at position 2 of the pyridine ring (Fig. 61).^67^

**Fig. 62:**
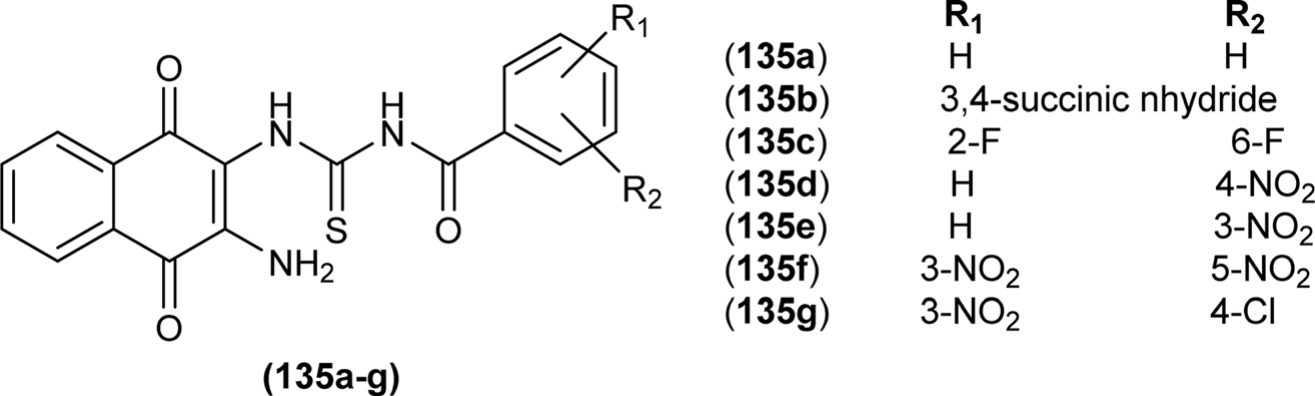
1,4-Naphthoquinone acyl thiourea hybrids.

The anti-inflammatory activity of seven novel 1,4-naphthoquinone acyl thiourea hybrids (135a–g) was investigated by evaluation of the production of 4 different cytokines (TNFα, IL6, IL12p40, GM-CSF) and was performed in presence and absence of LPS (Fig. 62). In the control negative group the production of cytokines was zero while in the positive control group production of cytokines was of high values, for example maximum production of different cytokines are TNFα, IL 6, IL12p40 and GM-CSF 7800, 5500, 6500 and 800, respectively. From these analyses it was concluded that the seven acyl thiourea exhibit potent anti-inflammatory activity and activity was found dose-dependent, thus by increasing the concentration, the anti-inflammatory activity of the acyl thiourea increases.^22^

**Fig. 63:**
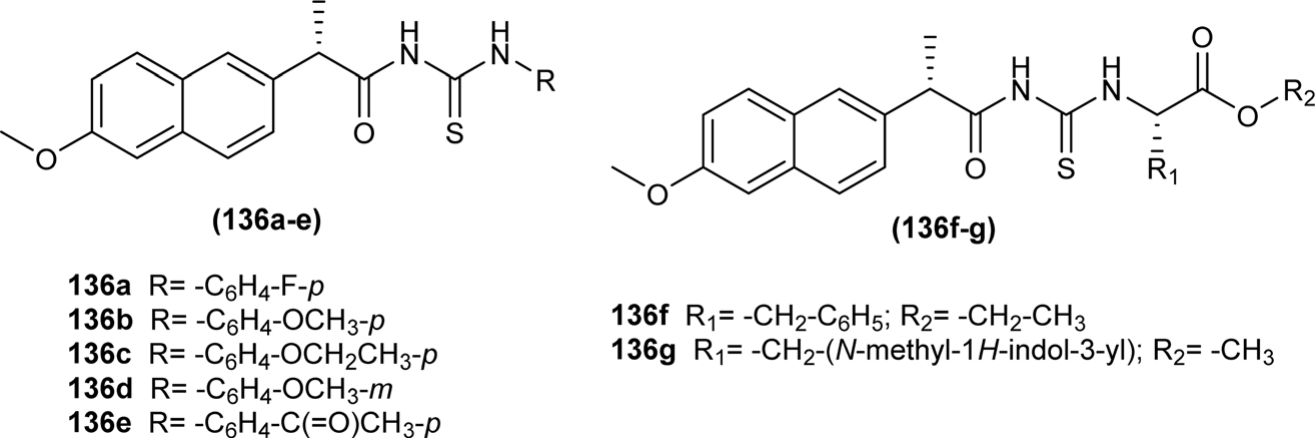
Naproxen substituted acyl thioureas.

All acyl thiourea derivatives of naproxen (136a–g) exhibit prominent anti-inflammatory activity with percentage inhibition in between 47% to 54% at the fourth hour following injection of carrageenan. Amongst the series compound (136d) at the highest dosage show percentage inhibition of 41.55% and 54.01% in the third and fourth hour, respectively. While compound (136g) was found more potent anti-inflammatory agent at the fourth hour after an injection of carrageenan with a percentage inhibition of 54.12% (Fig. 63).^120^

**Fig. 64:**
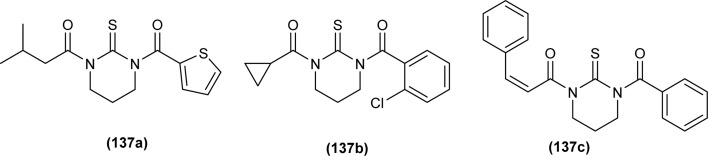
Structures of cyclic diacyl thioureas.

Spallarossa *et al.* synthesized a series of mono and diacyl thioureas as modulators of cystic fibrosis transmembrane conductance regulator (CFTR) protein. The synthesized compounds when investigated alone, very slightly affect the conductance of F508del-CFTR. When acyl thioureas were used in combination with Lumacaftor (LUM), certain derivatives have the potential to boost the effectiveness of the approved modulator. Some derivatives such as (137a) (+5%), (137b) (+8%), and (137c) (+16%) exhibited prominent additive effects. Among the series compound 137c was found more effective diacyl thiourea in enhancing the effect of LUM (Fig. 64).^121^

**Fig. 65:**
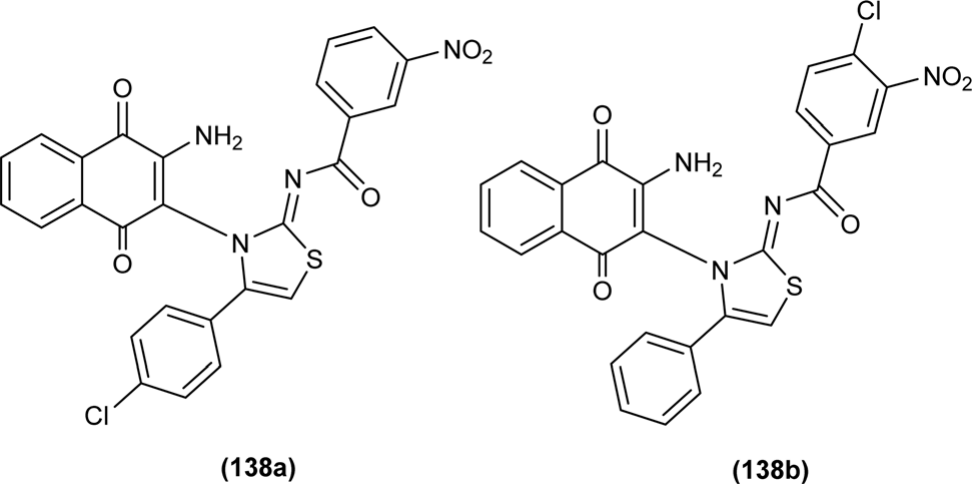
Structures of naphthoquinone–thiazole hybrids.

Naphthoquinone thiazole hybrids synthesized by Efeoglu *et al.* were screened for their anti-inflammatory potential. All compounds showed anti-inflammatory activity and among the series compounds (138a and 138b) were found more effective, these compounds act by lowering the production of inflammatory cytokines (IL-6 and TNF-α) in LPS-stimulated cells. An inverse molecular docking study was performed to find the mechanism of action, and PI3K was found as a potential target for synthesized hybrid derivatives (Fig. 65).^47^

**Fig. 66:**
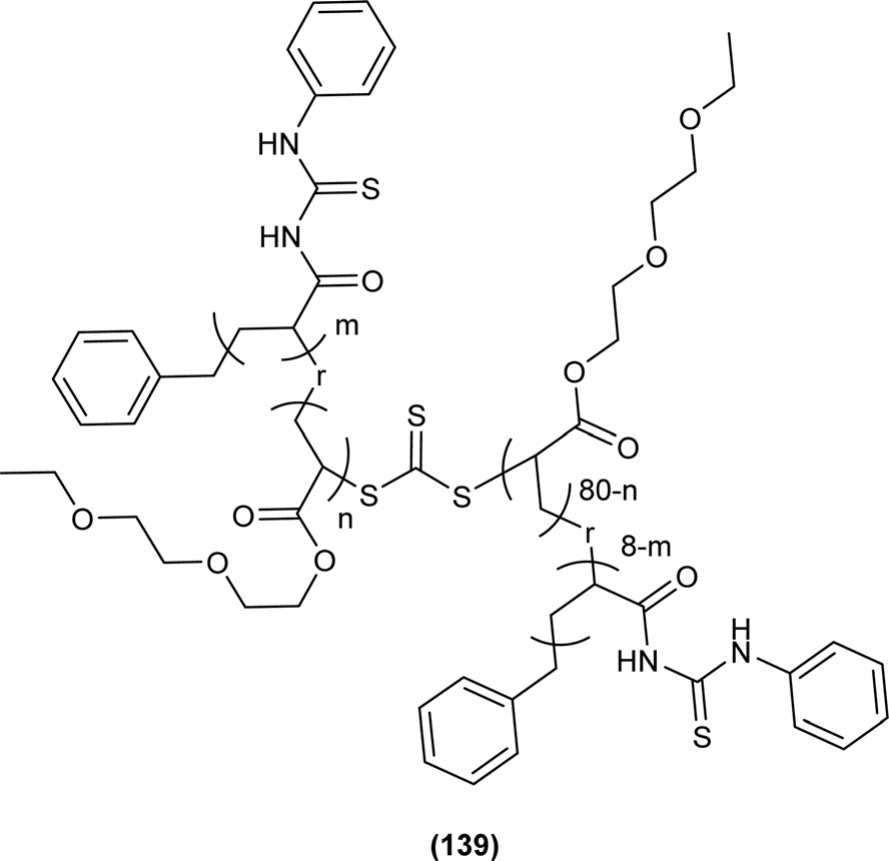
Copolymer containing acyl thiourea moiety in side chain.

Copper-coordinated metallo-supramolecular polymer, a random copolymer, comprises of poly(oligo(ethyleneglycol)ethylacrylate-*r*-acylthiourea)(P(OEGEA-*r*-ATU)) (139) having acyl thiourea moiety in side chain and act as a ligand to coordinate with Cu metal (Fig. 66). Compound (139) and Cu complex produced rod-like nano-objects due to coordination interaction and self-assembly of the complex. Coordination interaction of Cu with acyl thiourea moiety of (139) affects polymer chain configuration and gives rise to rod-like nano-objects by nanoprecipitation procedure.^122^

#### 6.1.11 Anti-hemolytic activity

Synthesized compounds (62a–e) by de Dorairaj *et al.* were found to show less than 5% lysis effect on Red blood cells (RBCs), indicating its potential as anti-hemolytic agent. It was also found that the acyl thiourea ligands show less hemolytic properties than metal complexes, thus ligands are more potent anti-hemolytic agents (Fig. 12).^78^

#### 6.1.12 Anti-hemorheological activity

**Fig. 67:**
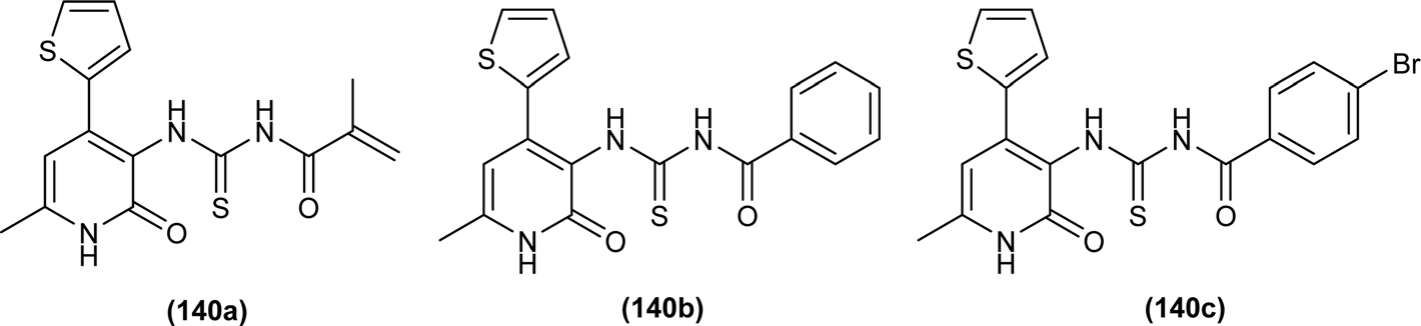
Acyl thiourea derivatives of 4-(thien-2-yl)-3-aminopyridine-2(1*H*)-one.

*In vitro*, hemorheological study of acyl thiourea derivatives of 4-(thien-2-yl)-3-aminopyridine-2(1*H*)-one (140a–c) showed that these compounds decrease the production of PGE2 and TXB2 in human platelets, which is directly responsible for the inhibition of cyclooxygenase-1 (COX-1) enzyme. A molecular docking study for antithrombotic activity also verified the result and free energy of acyl thiourea with selective site of proteins are higher than normal ligand interaction with proteins (Fig. 67).^123^

#### 6.1.13 Enzyme inhibition

*N*-Benzoylthioureapyrrolidine carboxylic acid derivatives (93aa–ae, 93ba–bb) synthesized by Poyraz *et al.* were investigated against AChE and BChE enzymes. All the derivatives had more than 65% inhibition ability at 10^−3^M concentration, compounds (92ab, 92ac, and 93ab) were most potent and had inhibitory ability greater than 88% at 10^−4^ M concentration against AChE enzyme. Synthesized derivatives had less than 50% inhibition against the BChE enzyme. From analysis of the most potent three compounds, it was concluded that the bicyclic compounds were better inhibitors than others. Overall compound (92ab) was concluded as the most potent inhibitor of AChE enzyme (Fig. 31).^63^

**Fig. 68:**
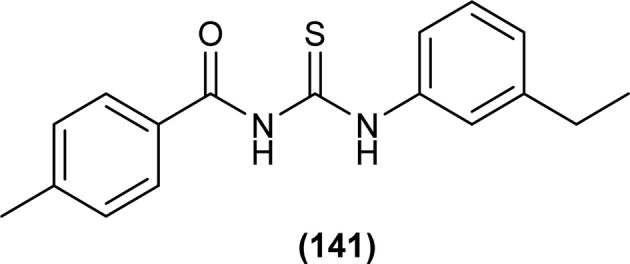
Structure of *N*-((3-ethylphenyl)carbamothioyl)-4-methylbenzamide.

*N*-((3-Ethylphenyl)carbamothioyl)-4-methylbenzamide (141) synthesized by Ahmed *et al.* showed strong binding interactions with carbonic anhydrase having a binding energy value of −6.6 kcal mol^−2^. Molecular docking study showed that carbonyl oxygen and thiocarbonyl sulfur in (141) formed hydrogen bonds with amino acids ASN67, GLN 92 and PRO 201, respectively. Pi-donors, hydrogen interactions of phenyl ring with amino acid ASN62 and pi-sigma interaction of phenyl with LEU198 were also shown by docking studies (141) also showed alkyl and pi-alkyl interactions with amino acids VAL121, HIS119, ALA65 and HIS96. From various intermolecular interactions and binding energy values, it was concluded that the compound (141) showed the best inhibition potential against carbonic anhydrase (Fig. 68).^66^

**Fig. 69:**
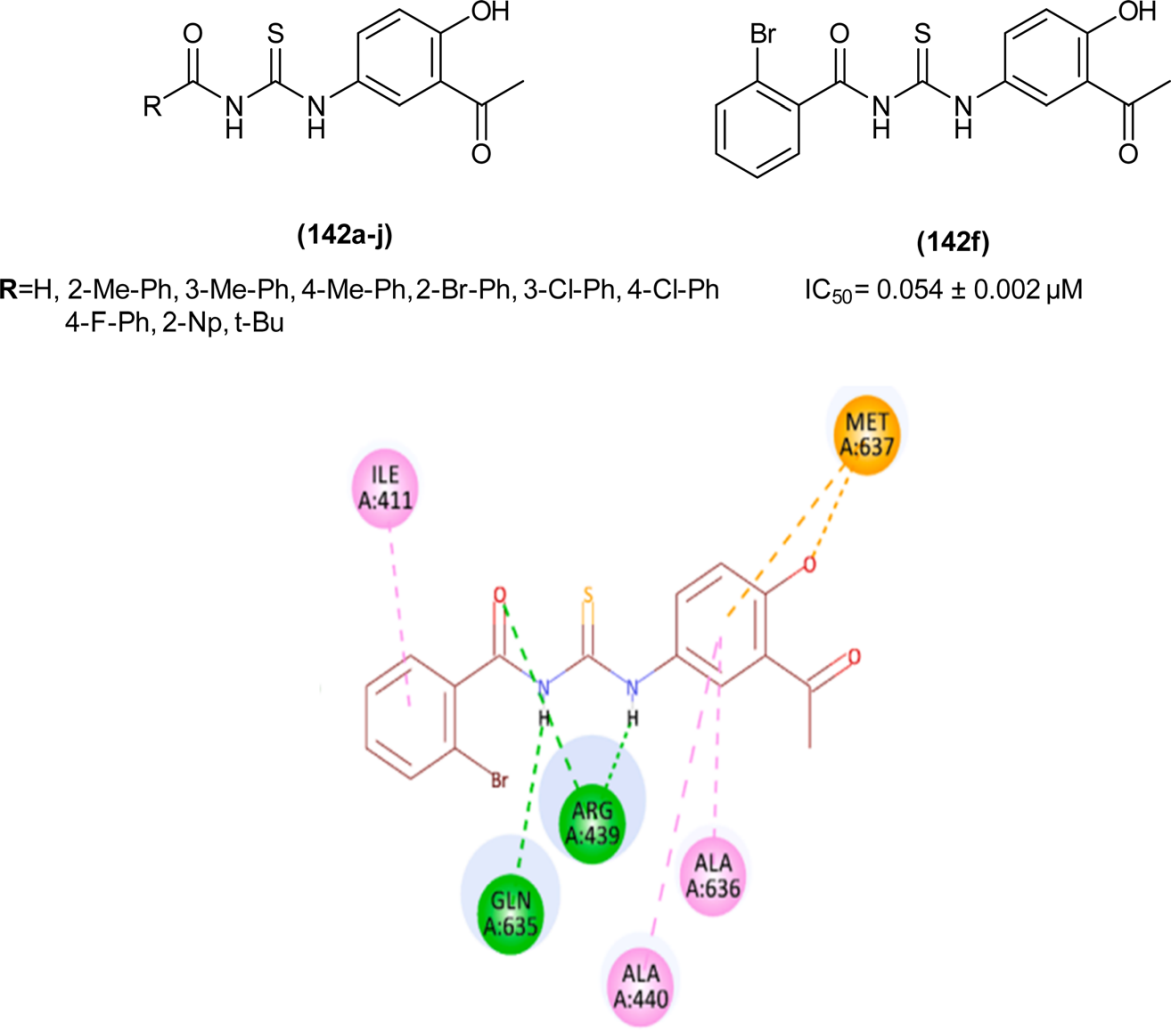
Acetylphenol-based acyl thioureas and interaction of compound 142f with various amino acid residues.

New acetylphenol-based acyl thioureas (142a–j) were synthesized by reacting different isothiocyanates with 1-(5-amino-2-hydroxyphenyl)ethan-1-one and investigated for urease inhibitory activity by Zahra *et al. In vitro* evaluation of the series revealed better inhibitory activity against urease enzyme than simple thiourea, and emphasized the role of different substituents present on nitrogen atom. Almost all compounds in the series had good activity but compound (142f) was the most potent agent with an IC_50_ value of 0.054 ± 0.002 μM, 413 times more efficient than standard drug against urease enzyme. The electron-donating substituent on the phenyl ring increased the inhibitory activity against urease. The presence of hydroxyl and acetyl groups was responsible for hydrogen bond interactions with different amino acids such as ARG609, HIS593, and MET637. *In silico* study also showed various types of intermolecular interactions like π-alkyl, π-sulfur and hydrogen bond formation with amino acids such as MET637, ARG439, ILE411, ALA440, GLN635 and ALA636. The stability of protein and its complex with inhibitor was also confirmed from molecular docking simulations. Pharmacokinetic study suggested that the tested compounds were safe to develop as drugs and could pass blood–brain barrier (Fig. 69).^124^

**Fig. 70:**
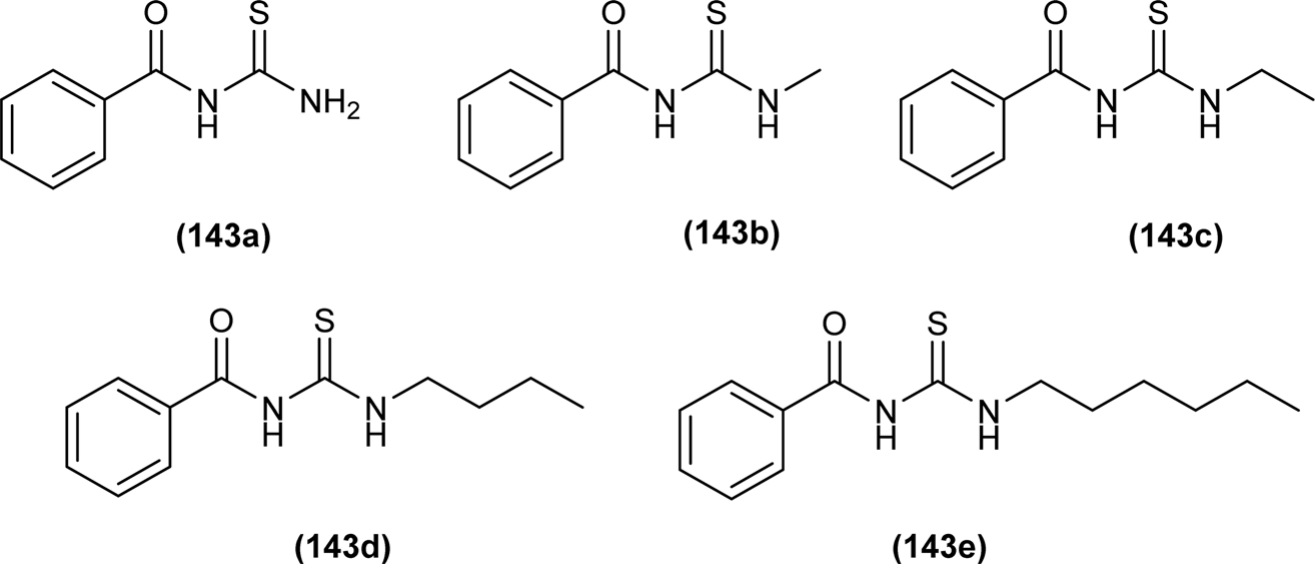
*N*-Alkylated benzoyl thiourea derivatives.

Tavares *et al.* performed biophysical and theoretical studies to investigate the effect of *N*-alkyl chain length in acyl thiourea on urease inhibition. Analysis from IC_50_ values, theoretical studies, and binding constant showed that the compound (143a) without having an alkyl chain (R=H) on the nitrogen atom was more potent than other alkyl chains containing acyl thioureas. Anti-urease inhibition activity decreased as the length of the alkyl chain increased, so the inhibition activity order of different acyl thioureas was (143a > 143b > 143c > 143d > 143e). (143a) -complex had binding constants value (*K*_b_) ranging from 7.95 to 5.71 × 10^3^ M^−1^ at different temperatures (22, 30, and 38 °C), H-bonding and van der Waal's forces were responsible for the stability of these complexes. (143a) Showed strong urease inhibitory activity in soil samples as efficient as NBPT with an average inhibition of 20% of urease activity. Computational studies of (143a) suggested that the molecule interacted at the allosteric site of the enzyme and appeared as a mixed inhibitor. It was concluded based on the biophysics and theoretical studies that increasing the chain length of the alkyl group did not favor urease inhibition (Fig. 70).^125^

**Fig. 71:**
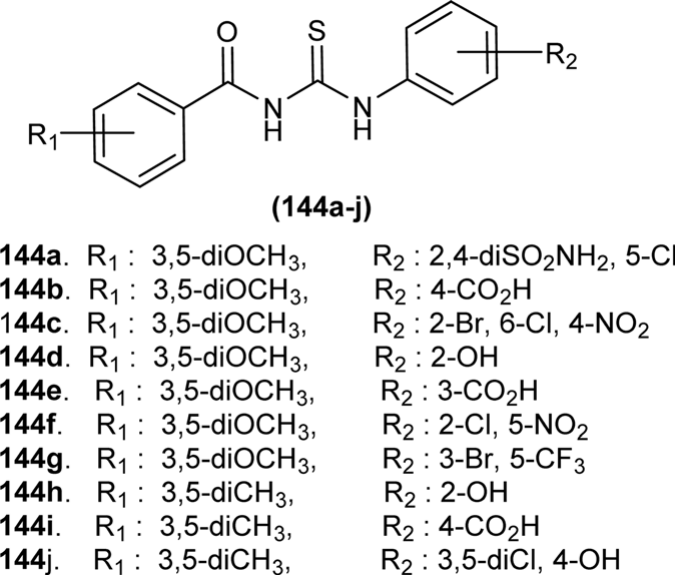
Structures of acyl thiourea derivatives 144a–j.

Hussain *et al.* synthesized 1,3-disubstituted thiourea (144a–j) derivatives and evaluated them for their carbonic anhydrase inhibiting ability against CA-II, hCA IX, and hCA-XII. All compounds showed good inhibitory activity against hCA-II in between 0.18 and 4.45 μM. Compounds (144f and 144i) showed prominent inhibition activity with IC_50_ values of 0.26 ± 0.03, and 0.38 ± 0.09 μM, respectively. Potency against carbonic anhydrase of compound (144f) was due to the presence of methoxy groups at *meta* position on ring A and B and chloro group at *para* position on ring B, while the presence of methyl group at *ortho* and *para* position on ring A and carboxylic acid group at *para* position on ring B was responsible for the high activity of the compound (144i). Compounds (142c and 142h) showed inhibition with IC_50_ values of 0.725 ± 0.068, and 1.24 ± 0.96 μM, respectively. All the compounds showed good activity against tumor-associated isoforms hCA-IX and hCA-XII with inhibition values between 0.17 to 14.58 μM. Activity of compound (144b) with IC_50_ value of 0.21 ± 0.09 μM was due to the presence of the carboxy group and methoxy groups. Compounds (144a, 144c, 144h, 144f, 144i, and 144j) with IC_50_ values 1.71, 1.01, 1.25, 4.93, 9.76, and 1.28 μM showed good inhibition activity compared to standard drug. Compounds having electron withdrawing groups at ring B exhibited more inhibitory activity than compounds having electron donating groups (Fig. 71).^126^

Acyl thioureas and thiosemicarbazides of 1-cycloalkane acid chlorides were synthesized and investigated against dihydrofolate reductase (DHFR) by Kholodniak *et al. In vitro* study revealed that cycloalkane substituted acyl thiourea did not show significant inhibitory activity while thiosemicarbazides (97a–h) were found more potent and percent inhibition value ranges between 28.34 and 90.32% for DHFR, except compound (97g). SAR analysis showed that diacylsemicarbazides were more potent inhibitors of DHFR than acyl thioureas. The inhibitory activity of compounds was determined by the nature of acyl group present, thus compounds (97a) cyclopropane, (97b) phenoxy, (97c) phenylthiol and (97f) furyl showed high activity because of the presence of the electron-donating groups, while compounds (97d, 97e, 97g and 97h) showed less activity due to presence of electron-withdrawing groups. Increasing the size of the moiety, increased the inhibitory activity of compounds against DHFR. Compound (97f) inhibited enzyme up to 90.32% due to the presence of cyclohexyl and pharmacophoric furan moiety (Fig. 34).^98^

**Fig. 72:**
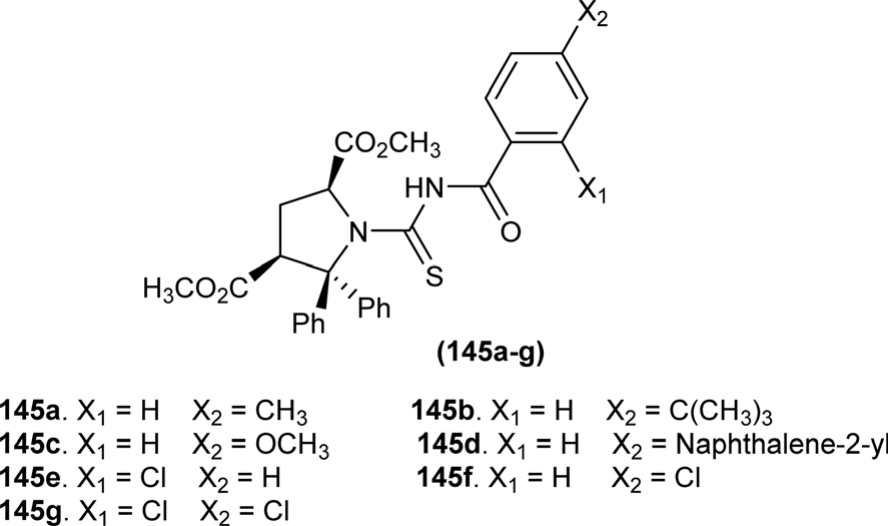
Structures of aroylthioureas 145a–g.

Novel acyl thiourea derivatives (145a–g) were synthesized and investigated against aldose reductase (AR), sorbitol dehydrogenase (SDH) and α-glucosidase enzymes by Ertano *et al.* All compounds showed good inhibition activity toward AR and SDH enzymes, compound (145c) (*K*_i_: 0.200 ± 0.024 μM) was found most potent against the AR enzyme. Acyl thiourea containing methoxy group at *para* position on benzene ring was 10.97 times more potent inhibitor than acyl thiourea having methyl group present at *para* position on the benzene ring. The sequence of the inhibitory effect of different substituents on benzene ring at the *para* position was OMe > Cl > *t*-butyl > methyl. Compound (145g) (*K*_i_: 0.114 ± 0.01 μM) exhibited the highest inhibitory activity against SDH. The presence of chlorine groups at the 2 and 4 positions on the benzene ring instead of the naphthoyl group at position 2 increased inhibitory activity about 61.75 times against SDH enzyme. This enhancement was due to the presence of electron-withdrawing inductive effect of an electronegative chlorine atom. Acyl thiourea having chlorine atoms present at both 2 and 4 positions of the benzene ring was more potent against SDH than in cases where Cl was attached at either 2 or 4 positions, due to the synergistic effect of two chlorine atoms. Similarly, compound 145g (*K*_i_: 0.055 ± 0.01 μM) showed the best activity against α-glucosidase. Attachment of different groups to the benzene ring at *para* position showed inhibition activity against α-glucosidase in the following order, methoxy > methyl > chloro > *tert*-butyl (Fig. 72).^127^

Compound (44) was found good inhibitor of urease enzyme with IC_50_ value in the range 0.0389 ± 0.0017 μM as compared to standard thiourea (18.2 ± 0.297 μM). The synthesized acyl thiourea (44) not only showed better antiurease activity compared to thiourea but also this compound was more potent than complexes of corresponding acyl thiourea. From docking studies, it was observed that compound (44) showed urease inhibition activity due to the presence of two H-bonding interactions. Free radical scavenging activity of the compound (44) was moderate (55.62%) compared to vitamin-C (94.90%) at 100 μg mL^−1^. *In silico* investigation of compound (44) revealed hydrogen bonding interactions in ligand–protein complexes during compound (44) binding with RNA, and found as a potent groove binder with DNA (Fig. 5).^68^

**Fig. 73:**
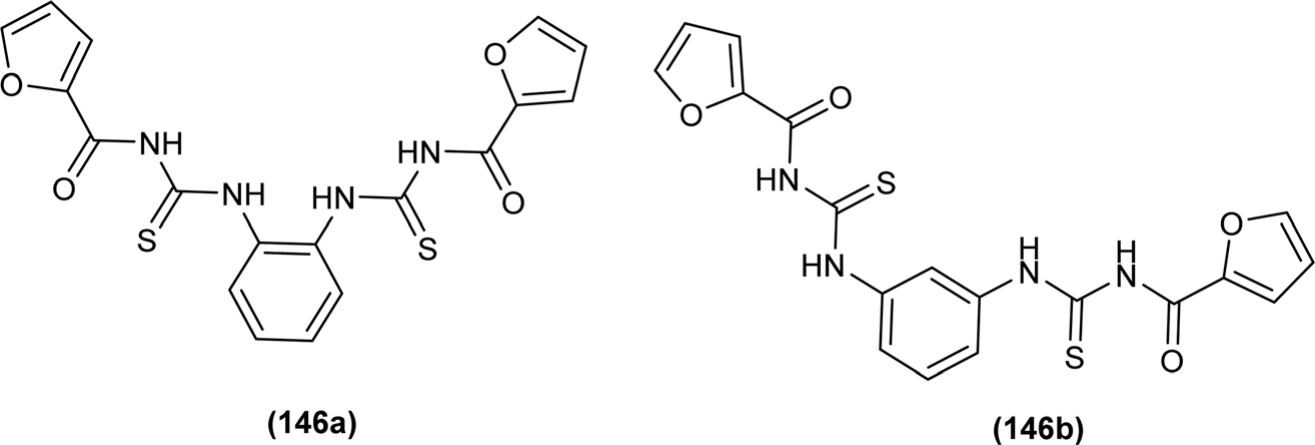
Structures of 1-(2-furoyl)thioureas.

Novel 1-(2-furoyl)thiourea derivatives were evaluated against *Bacillus pasteurii urease* (pdb id: 4ubp) by using a molecular docking study. All synthesized compounds showed urease inhibition activity, and compounds (146a and 146b) showed best docking score due to the presence of two thiourea moieties in molecules. This suggested that acyl thiourea having more than one thiourea moiety per molecule increased the inhibition activity against urease protein (4ubp) (Fig. 73).^115^

**Fig. 74:**
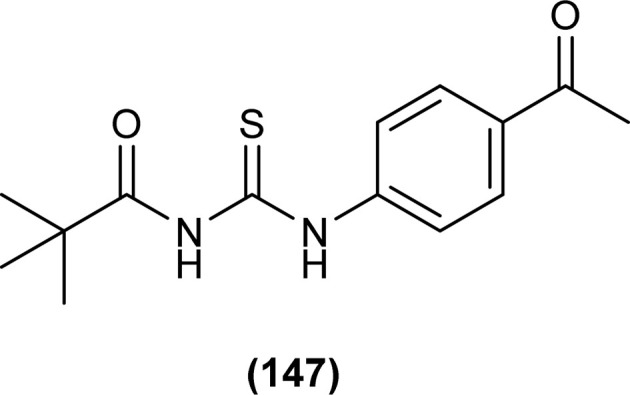
Structures of *N*-((4-acetylphenyl)carbamothioyl)pivalamide.

*N*-((4-Acetylphenyl)carbamothioyl)pivalamide (147) was found effective inhibitor of butyl cholinesterase (BChE), acetyl cholinesterase (AChE), alpha amylase, and urease enzyme. Inhibitory activity of the compound (147) was 85% for butyrylcholinesterase and acetylcholinesterase, while the inhibitory activity was about 73.8% and 57.9% against urease and α-amylase, respectively. IC_50_ values of compound (147) were lower, 26.23 and 30.9 ppm for AChE and BChE, respectively but higher for α-amylase and urease enzymes (160.33 and 91.5 ppm respectively). Docking results also verified the above results, AChE and BChE showed strong bonding interactions with compound (147) having binding energy −7.5 kcal mol^−1^ and −7.6 kcal mol^−1^, respectively (Fig. 74).^128^

**Fig. 75:**
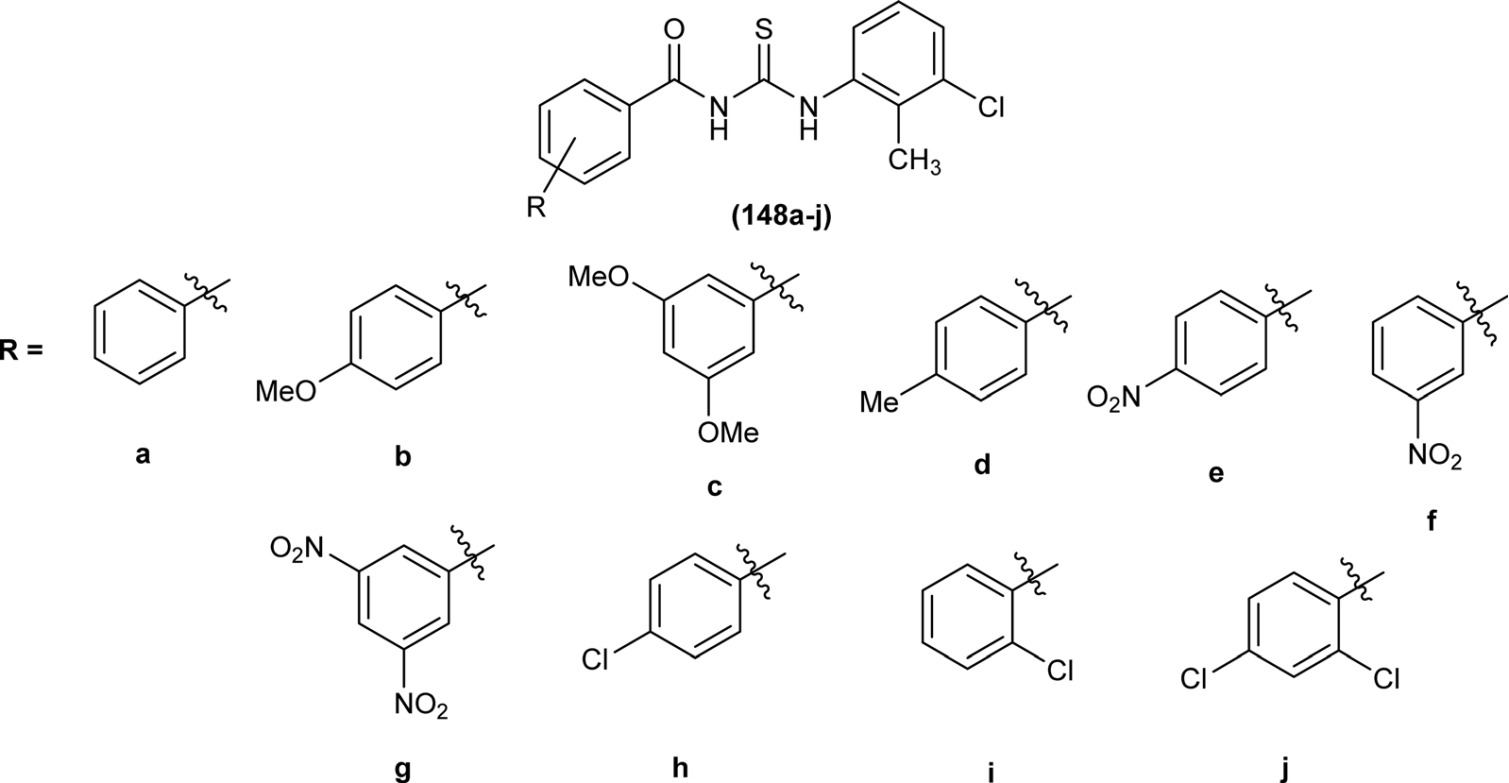
1-Aroyl-3-(3-chloro-2-methylphenyl)thiourea hybrids.

1-Aroyl-3-(3-chloro-2-methylphenyl)thiourea hybrids (148a–j) were synthesized as effective inhibitors of urease by Rasheed *et al.* All synthesized compounds exhibited good inhibitory activity against jack bean urease (JBU) having IC_50_ values in the range of 0.0019 ± 0.0011 to 0.0532 ± 0.9951 μM as compared to standard thiourea with IC_50_ 4.7455 ± 0.0545 μM. Compounds (148i and 148e) were concluded as most potent inhibitors of urease with IC_50_ values 0.0019 ± 0.0011 μM and 0.0038 ± 0.0784 μM, respectively (Fig. 75).^129^

**Fig. 76:**
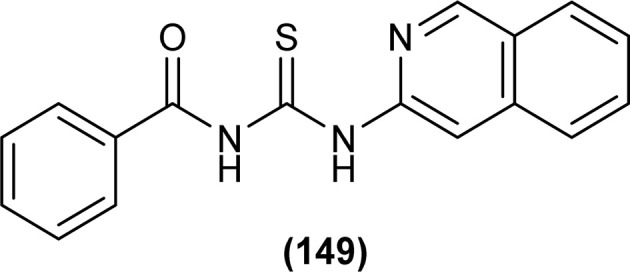
Quinoline substituted thiourea.

Novel acyl thiourea (149) was synthesized and subjected toward drug-likeness property and binding affinity with σ1R crystal structure 5HK1. Compound showed a good drug-likeness score of about 0.73 and a TPSA value of 86.11 Å. A molecular docking study revealed that stable conformation had a binding energy score of −10.5 kcal mol^−1^ and involved two H-bond interactions with amino acids GLU 172, TYR 103 of 5HK1. However, *in vitro* binding affinity was greater than 10 and thus not correlated with binding affinity determined through docking study (Fig. 76).^130^

**Fig. 77:**
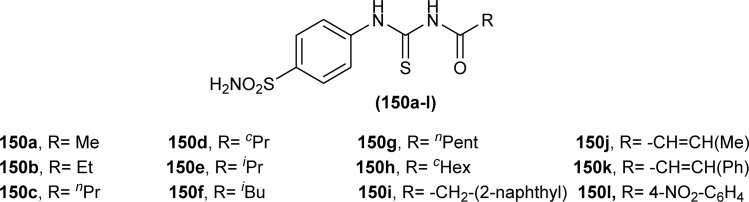
*N*-((4-Sulfamoylphenyl)carbamothioyl)amides.

A series of acyl thiourea (150a–l) was synthesized by acylation of 4-thioureidobenzenesulfonamide with various acid chlorides (Fig. 77). These compounds were investigated for their inhibition activity against three α-class cytosolic human (h) carbonic anhydrases (CAs) (EC 4.2.1.1) hCA I, hCA II and hCA VII and bacterial β-CAs from *Mycobacterium tuberculosis* (MtCA1–MtCA3). Majority of the compounds showed better inhibition activity against human carbonic anhydrase with inhibition constant value *K*_i_ = 13.3–87.6 nM, 5.3–384.3 nM, and 1.1–13.5 nM for hCA I, hCA II and hCA VII, respectively. These compounds also inhibited mycobacterial enzymes MtCA1, MtCA2 and MtCA3 with *K*_i_ values in the range of 95.2–6669.2, 3.4–57.1, and 446.6–9396.5 nM respectively, which revealed that the compounds had poor activity against MtCA3.^131^

A molecular docking study of *N*-((2-acetylphenyl)carbamothioyl)benzamide (47) was performed to determine its inhibitory potential against COVID-19 coronavirus's primary protease. Binding energy of the acyl thiourea ligand with protein 6LU7 was −4.93 kcal mol^−1^ due to hydrogen bond interactions of N and O of the ligand with amino acids Lys137, Gly138, and Cys128 (Fig. 7).^70^

**Fig. 78:**
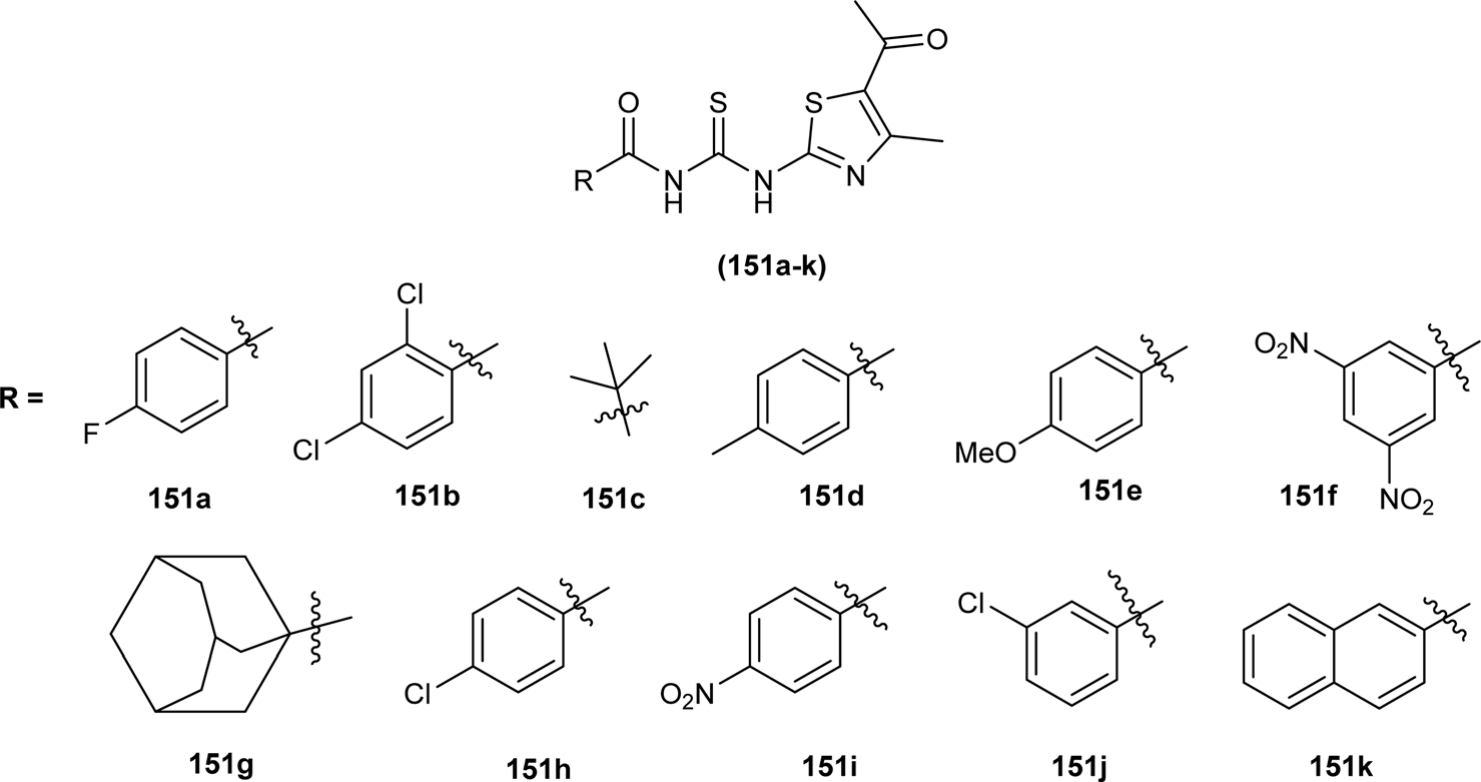
Thiazole-linked acyl thiourea derivatives.

A series of thiazole-linked acyl thioureas (151a–k) were synthesized and investigated for the inhibition of alkaline phosphatase, all synthesized compounds manifested good inhibition activity against AP, while compounds (151c, 151g and 151h) in series were found most potent inhibitors of AP with IC_50_ value of 0.057, 0.019 and 0.091 μM, respectively. The presence of 3 methyl groups, a carboxamide group and a chlorine atom in compounds (151c, 151g and 151h) was proposed as the reason for better activity. The docking study also supported the above results and it was found that the compounds (151c and 151g) showed strong binding of −32.18 and −30.09 kJ mol^−1^, respectively (Fig. 78).^132^

Naphthalene-based acyl thiourea conjugates (122a–j) were synthesized and evaluated as inhibitors of alkaline phosphatase by Saeed *et al.* All compounds exhibited good inhibitory activity against AP with IC_50_ values between 0.365 ± 0.011 and 4.225 ± 1.054 μM. Compounds (122h and 122a) were found most potent inhibitors of AP with IC_50_ values in the range of 0.365 ± 0.011 and 0.436 ± 0.057 μM, respectively. The presence of hydroxyl and phenyl groups in compounds (122h and 122a), at *ortho* position was deemed responsible for the better inhibitory activity. The docking study revealed that compounds (122h and 122a) showed the highest binding score of −8.1 kcal mol^−1^ and −7.5 kcal mol^−1^, respectively (Fig. 52).^116^

**Fig. 79:**
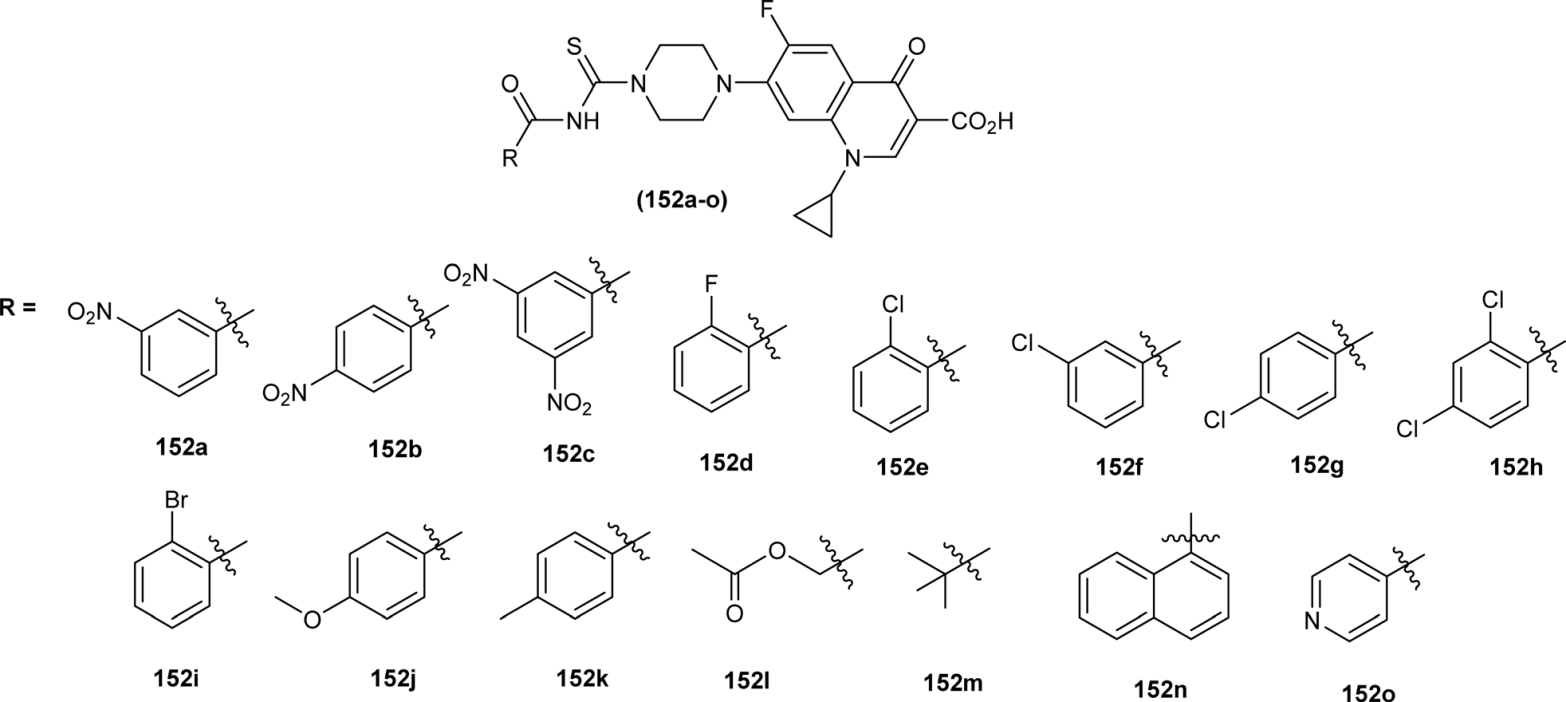
1-(Acyl/aroyl)-3-(ciprofloxacinyl)thioureas.

Novel series of 1-(acyl/aroyl)-3-(ciprofloxacinyl)thioureas (152a–o) were synthesized and evaluated for activity against carbonic anhydrase (CA-II) and 15 lipoxygenase (LOX) enzymes by Saeed *et al.* Compounds (152g) followed by (152f) were found most potent inhibitors with IC_50_ values of 0.97 ± 0.11 and 1.95 ± 0.12, respectively. None of the compounds were found effective against 15-LOX with % inhibition of all compounds less than 50% (Fig. 79).^133^

*In vivo* investigation of a new series of acyl thiourea (157a–l) was performed for acetyl hydroxylate synthase (AHAS) enzyme activity inhibition. Almost all compounds showed inhibition activity at a concentration of 100 mg L^−1^. Amongst the series compounds (157b and 157f) were found most potent inhibitors with % inhibition of 36.17% and 37.08%, respectively (Fig. 84).^134^

Novel series of Nimesulideiminothiazolines conjugates (8a–j) was synthesized and investigated for inhibition of acetylcholinesterase (AChE), butyrylcholinesterase (BChE), carbonic anhydrase I (hCA I) and carbonic anhydrase II (hCA II). All derivatives inhibited AChE and BChE much better than standard Tacrine (TAC), except compound (8e). *K*_i_ values for these compounds ranged from 81.48 ± 8.25 to 137.84 ± 11.04 nM against AChE. The most potent compounds were (8a, 8d and 8f) with *K*_i_ values 81.48 ± 8.25, 86.35 ± 6.92, and 90.24 ± 9.55 nM, respectively. Inhibition activity *K*_i_ of derivatives against BChE ranged from 61.35 ± 4.62 to 105.83 ± 11.08 nM, and the most effective compounds in the series were (8a, 8i, and 8d) with *K*_i_ values of 61.35 ± 4.62, 68.03 ± 8.85, and 70.12 ± 5.38 nM, respectively. Same was the case for inhibition of hCA I and hCA II, all the synthesized acyl thiourea derivatives were found more effective than the standard drug Acetazolamide (AZA) with *K*_i_ values ranging from 10.36 ± 1.56 and 46.96 ± 6.37 nM and 12.68 ± 1.78 to 49.65 ± 5.38.51 nM for hCA I and hCA II, respectively (Scheme 3).^43^

**Fig. 80:**
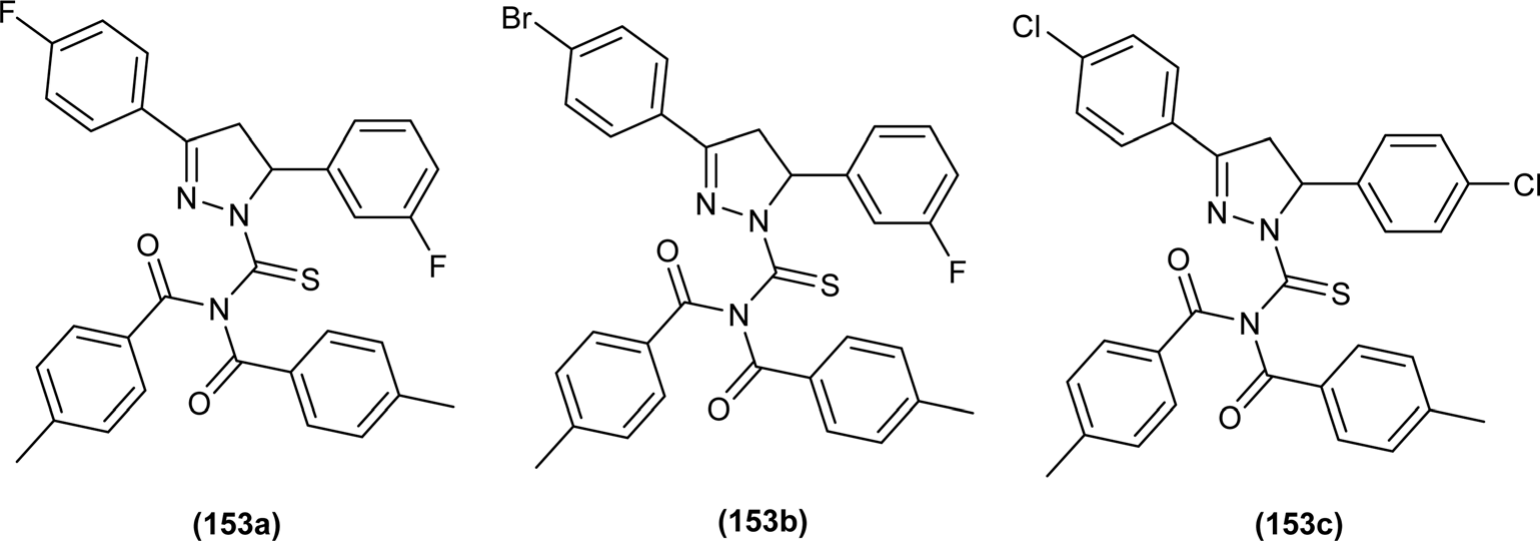
Pyrazolinyl based acyl thioureas.

A novel series of pyrazolinyl-linked acyl thiourea derivatives were synthesized by Saeed *et al.* and evaluated for their inhibitory activity against urease, amylase, and α-glucosidase. Among the series, compounds (153a and 153c) were found more potent inhibitors of urease with IC_50_ values of 54.2 ± 0.32 and 43.6 ± 0.25 μM, respectively. Whilst compounds (153a and 153b) exhibited prominent activity against α-glucosidase with IC_50_ values of 68.3 ± 0.11 and 90.3 ± 1.08 μM, respectively (Fig. 80).^135^

A series of iminothiazolidinone (26a–j) was evaluated as an inhibitor for carbonic anhydrase II enzyme. The IC_50_ values ranged from 1.545 ± 0.016 to 67.542 ± 2.714 μM. Compound (26e) exhibited better inhibitory activity against CA II with IC_50_ value 1.545 ± 0.016 μM (Scheme 7).^50^

Amantadine thiazolidinone derivatives synthesized by Ahmad *et al.* were found to exhibit good inhibitory activity against the enzyme elastase with IC_50_ values ranging from 0.221 ± 0.059 to 3.257 ± 0.541 μM. Compound 5 g showed highest potency against elastase with an IC_50_ value of 0.124 ± 0.022 μM which was 50 times higher than Oleanolic acid's value of 5.996 ± 0.882 μM.^51^

Triazole of acyl thiourea (36) synthesized by Babar *et al.* was investigated against Jack Bean Urease. Compound (36) was found 100 times more potent than standard acetohydroxamic acid (IC_50_ = 15.92 ± 0.035 μM) with IC_50_ value of 0.21 ± 0.2 μM, The activity of the compound was attributed to presence of 1,2,4-triazole moiety (Scheme 10).^54^

(*Z*)-4-Bromo-*N*-(4-butyl-3(quinolin-3-yl)thiazol-2(3*H*)-ylidene)benzamide (13b) was investigated against elastase. The IC_50_ 1.21 μM as compared to standard oleanolic acid with IC_50_ value of 13.45 μM authenticated the higher potency of the synthesized compound (Scheme 4).^44^

Novel quinolinyl iminothiazolines (13a–j) were investigated for their inhibitory activity against alkaline phosphatase. All the synthesized compounds exhibited good to excellent activity with IC_50_ values ranging from 0.337 ± 0.015 to 8.681 ± 0.908 μM. *N*-benzamide quinolinyl iminothiazoline (13g) was found most effective against with IC_50_ value 0.337 ± 0.015 μM (Scheme 4).^45^

*In silico* study of bis(acyl thiourea) derivatives (55–59) against various enzymes such as urease, VEGFR2, EGFR and SARS-CoV-2 main protease showed that the binding energy of the compounds (55–59) was much better than co-crystal ligand. Binding energies for the interaction of compounds (55–59) with active sites of enzymes were found in the ranges −(4.77–6.60), −(6.23–9.10), −(5.70–8.37) and −(6.45–8.82) for urease, VEGFR2, EGFR and SARS-CoV-2, respectively (Fig. 10).^73^

**Fig. 81:**
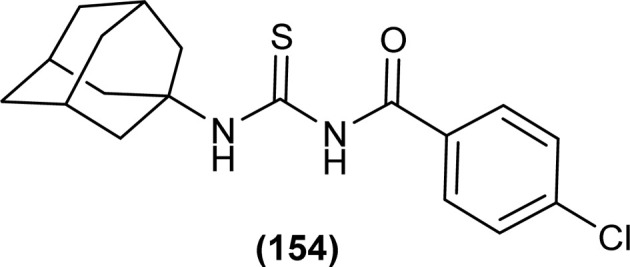
Amantadine linked acyl thiourea.

Arshad *et al.* synthesized and characterized three novel amantadine-linked acyl thiourea derivatives and screened for their anti-glioma, DNA binding and anti-elastase potentials. Studies showed that these thioureas proved as good DNA binders through mixed types of interactions. Comparatively compound (154) was found as stronger DNA binder through groove binding and partial intercalation as endorsed by DNA viscosity trend and low diffusion coefficient (*D*_0_). Similarly, among the three, compound (154) was found a more potent inhibitor of elastase enzyme with an inhibition zone of 86.5% and IC_50_ value of 1.48 μM. Newly synthesized amantadine linked acyl thiourea derivatives also exhibit potent anticancer activity against malignant glioma MG-U87 cells (Fig. 81).^136^

Naphthoquinone based iminothiazoline derivatives (14a–e) synthesized by Efeoglu *et al.* were found as effective inhibitors of carbonic anhydrase I and II, and acetylcholinesterase (AChE) and butyrylcholinesterase (BChE). Inhibitory constant *K*_i_ values of synthesized derivatives were in the range of 67.86–161.60 nM for hCA I, 55.27–87.48 nM for hCA II, 26.12–98.42 for AChE and 45.03–84.43 nM for BChE. Molecular docking study of derivatives showed that the compounds interact with the active sites of ChEs and hCAs (Fig. 2).^46^

#### 6.1.14 Herbicidal activity

**Fig. 82:**
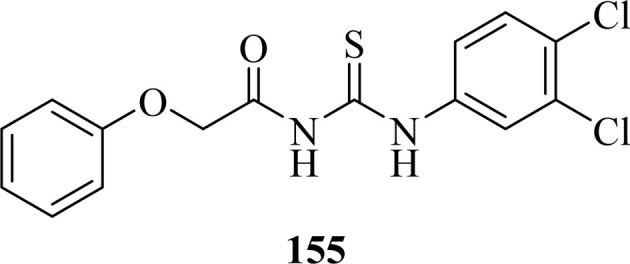
Structure of 1-(3,4-dichlorophenyl)-3-(2-phenoxyacetyl)thiourea.

Sonoda *et al.* synthesized aryloxy thiourea 1-(3,4-dichlorophenyl)-3-(2-phenoxyacetyl)thiourea (155) and its derivatives from different substituted phenols, 2-chloroacetamide and *N*-aryl isothiocyanates and investigated for the radicle elongation inhibitory activity, compound (155) was found as most potent inhibitor of *Orobanche minor* seeds germination (Fig. 82).^40^

**Fig. 83:**
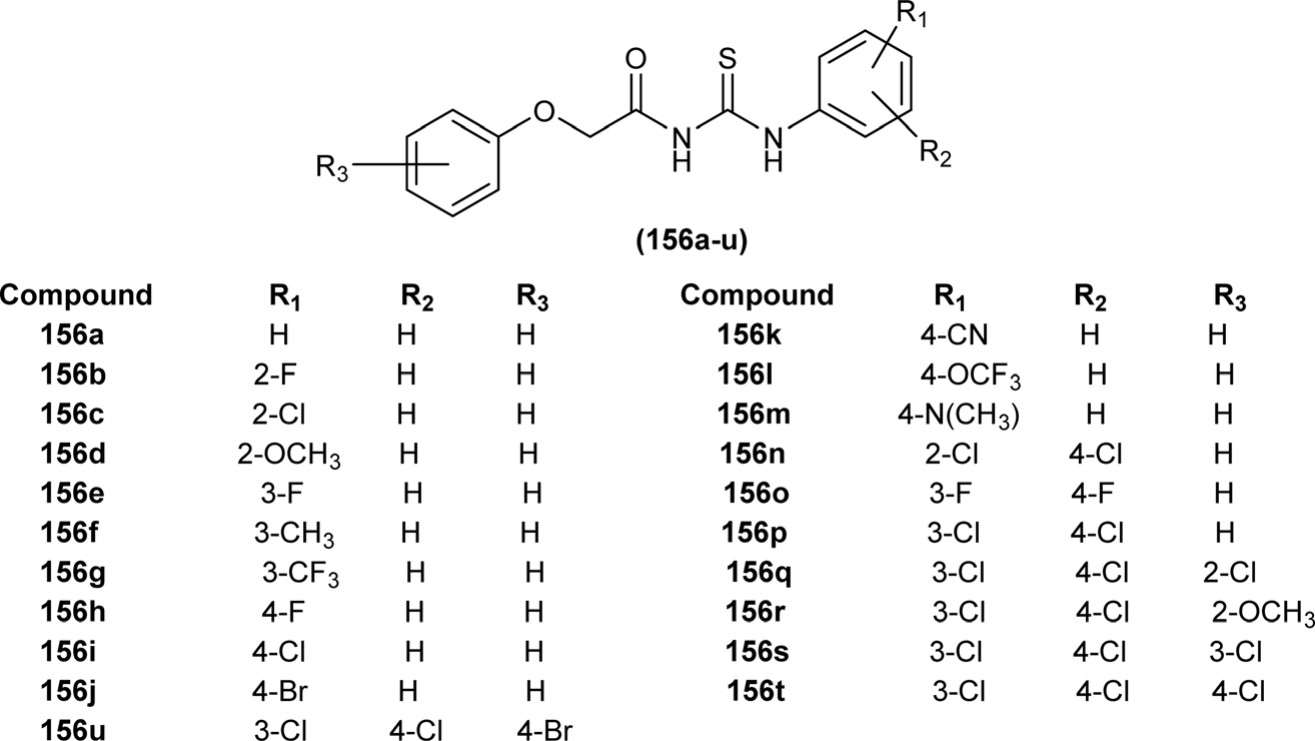
Aryloxyacetyl thiourea derivatives.

Inhibitory activity of the new *N*-acylated thiourea series (156a–u) was evaluated against *O. minor* radicle elongation. 17 out of 21 exhibited inhibitory activity in dose dose-dependent manner, except compounds (156a, 156c, 156d and 156f) which did not exhibit any inhibitory activity. Compounds (156s and 156t) having chlorine substituents on benzene ring of phenoxy moiety had an inhibitory activity of less than 30% at a concentration of 1 ppm (Fig. 83).^137^

**Fig. 84:**
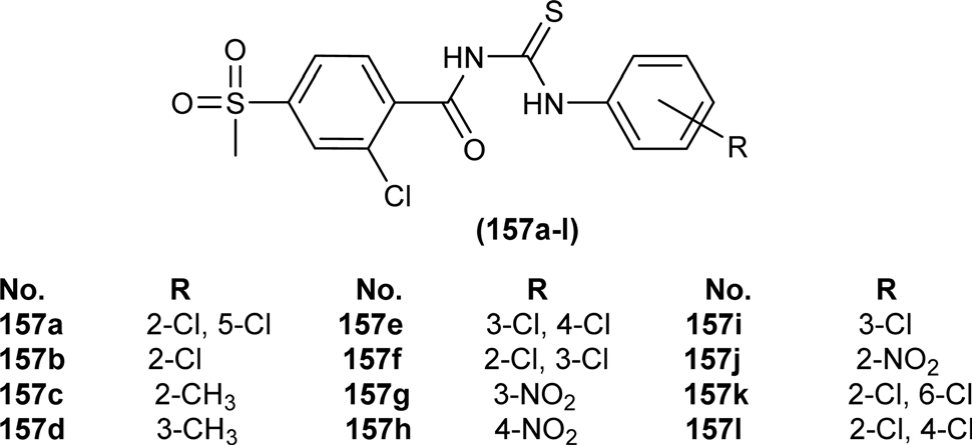
2-Chloro-4-(methylsulfonyl)phenyl based thioureas.

A new series of acyl thiourea derivatives (157a–l) exhibited good herbicidal activity against *Digitaria adscendens* and *Amaranthus retroflexus* with a % inhibition range from 30.48 ± 2.13 to 82.10 ± 1.77 at a concentration of 100 mg L^−1^. The activity of the synthesized compounds was found to be dose-dependent. Results analysis for pre-emergence applications showed that all compounds were 40% effective, while compounds (157b and 157f) exhibited a 78 and 69% inhibition rate, respectively against *D. adscendens*. The inhibition rate of the series was not good against *A. retroflexus* except for compounds (157b and 157f) which showed percent inhibition of 68 and 60%, respectively. For post-emergence application, all the synthesized compounds (157a–l) showed less than 40% inhibitory activity against *D. adscendens* and *A. retroflexus* (Fig. 84).^134^

#### 6.1.15 Insecticidal activity

**Fig. 85:**
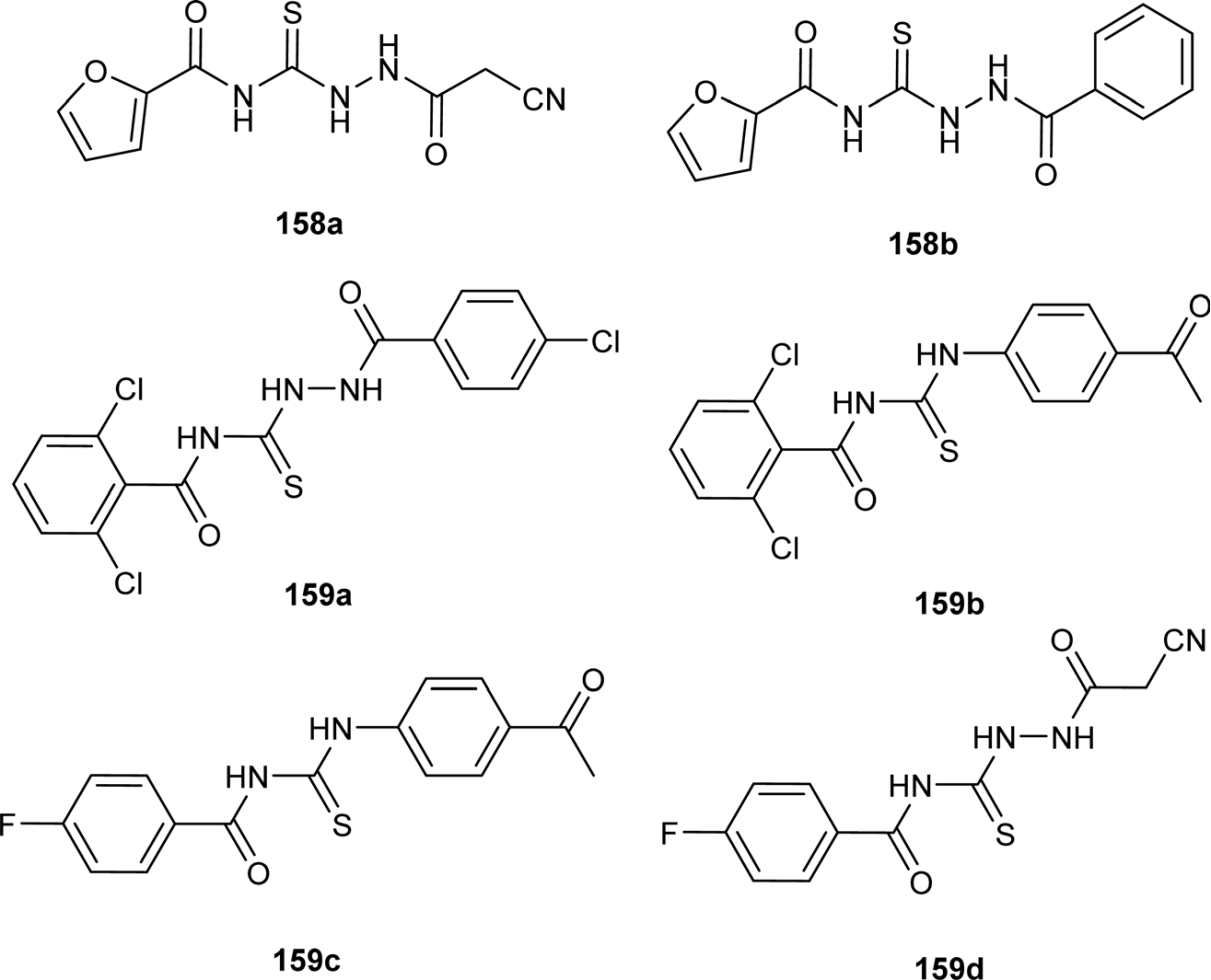
*N*-Acylated thioureas and thiosemicarbazides.

Novel thiourea and thiosemicarbazide having acyl thiourea functionality were synthesized and evaluated as insect (*Spodoptera littoralis*) growth regulators by Abdelhamid *et al.* Compounds (158a–d and 158a–d) tested against second larvae of insect showed diverse activity range from low LC_50_ values 26.63 ppm with high activity to very high LC_50_ values 313.11 ppm with low activity. Compound (159d) (LC_50_ 26.63 ppm) showed very close activity with the standard Lufenuron (LC_50_ 17.01 ppm) against the second larva of the instar insect. Compounds also show toxicological activity against fourth larvae and LC_50_ values ranged from low to very high (145.90 to 1588.36 ppm). Compounds (159a and 159d) with LC_50_ values 145.90 and 148.56 ppm showed comparable activity to that of Lufenuron with LC_50_ values 103.12 ppm against 4th larvae of insect. SAR study revealed that compound (159d) manifested highest activity due to the presence of electron-withdrawing fluorophenyl and cyano groups in the molecule. While the activity of compound (159a) was due to the presence of the dichlorophenyl group. Oxadiazole derived from thiosemicarbazide (158a and 158b) by cyclization was also found effective as a growth regulator and compound (158c) showed better insecticidal activity with LC_50_ value 73.35 ppm against second larvae instar (Fig. 85).^49^

**Fig. 86:**
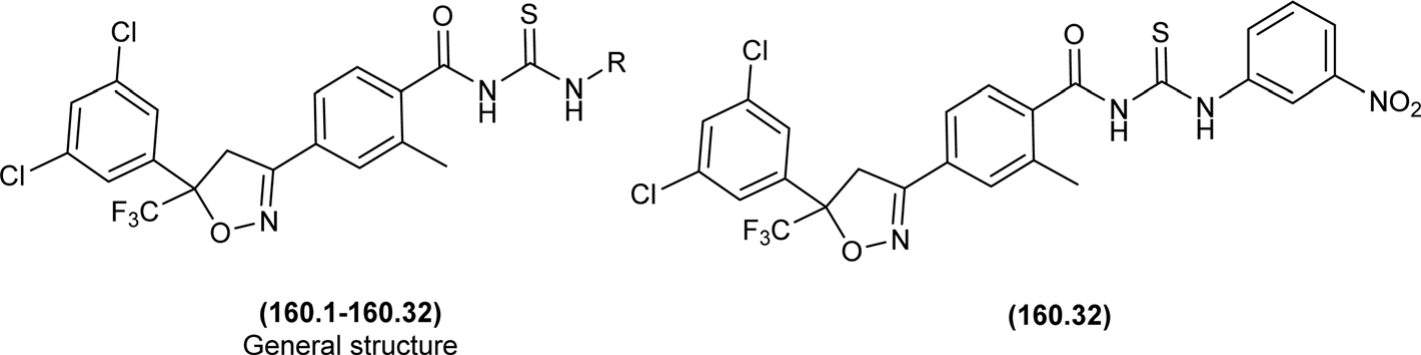
Isoxazoline containing acyl thioureas.

A novel series of isoxazoline-containing acyl thiourea (160.1–160.32) was evaluated for their insecticidal activity against *Plutella xylostella*. All compounds showed good activity with LC_50_ values ranging from 0.26 to 59.89 mg L^−1^. Insecticidal activity was dose-dependent and exhibited 100% mortality at 100 mg L^−1^ and 80% mortality was shown by half of the compounds at 10 mg L^−1^. From SAR study and structure optimization, a new compound (160.32) was designed and synthesized which was found more potent against *P. xylostella* having an LC_50_ value of 0.26 mg L^−1^ and was better insecticidal agent than standard Ethiprole (LC_50_ = 3.81 mg L^−1^), Avermectin (LC_50_ = 12.32 mg L^−1^), and compounds (160.1–160.31) (Fig. 86).^138^

**Fig. 87:**
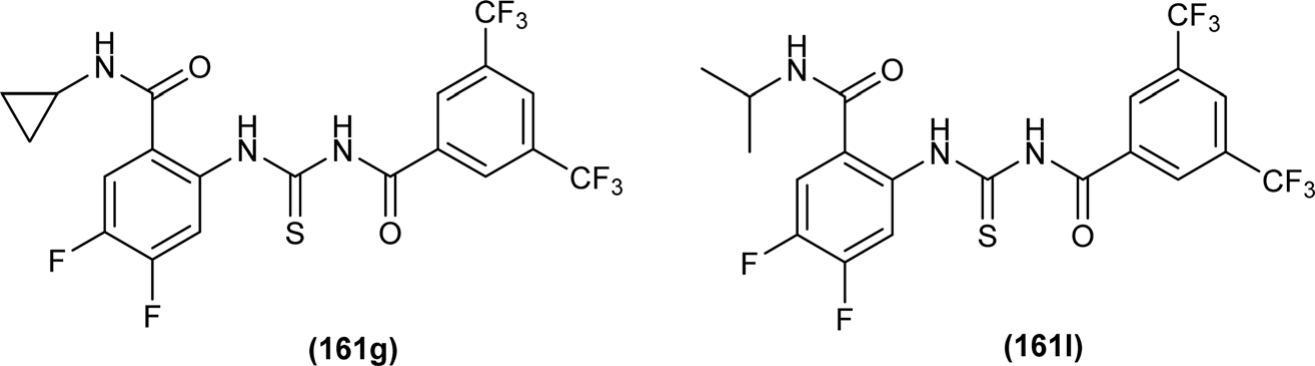
Anthranilic diamides containing acyl thiourea substructure.

A series of novel acyl thiourea derivatives (161a–s) was evaluated for their mosquito larvicidal activity against *C. quinquefasciatus* and *A. aegypti* 3rd instar larvae. Compound (161l) showed the highest larvicidal activity with LC_50_ values of 0.0044 mM and 0.0070 mM, and LC_90_ values of 0.0058 mM and 0.0103 mM against *C. quinquefasciatus* and *A. aegypti*, respectively. Compound (161g) was also a potent insecticidal agent with LC_50_ values of 0.0068 mM and 0.0085 mM against *C. quinquefasciatus* and *A. stephensi* (Fig. 87). Morphological changes which ultimately lead to the death of the larvae were setae and anal ventral brush damage, abdominal alterations and extended necks.^139^

**Fig. 88:**
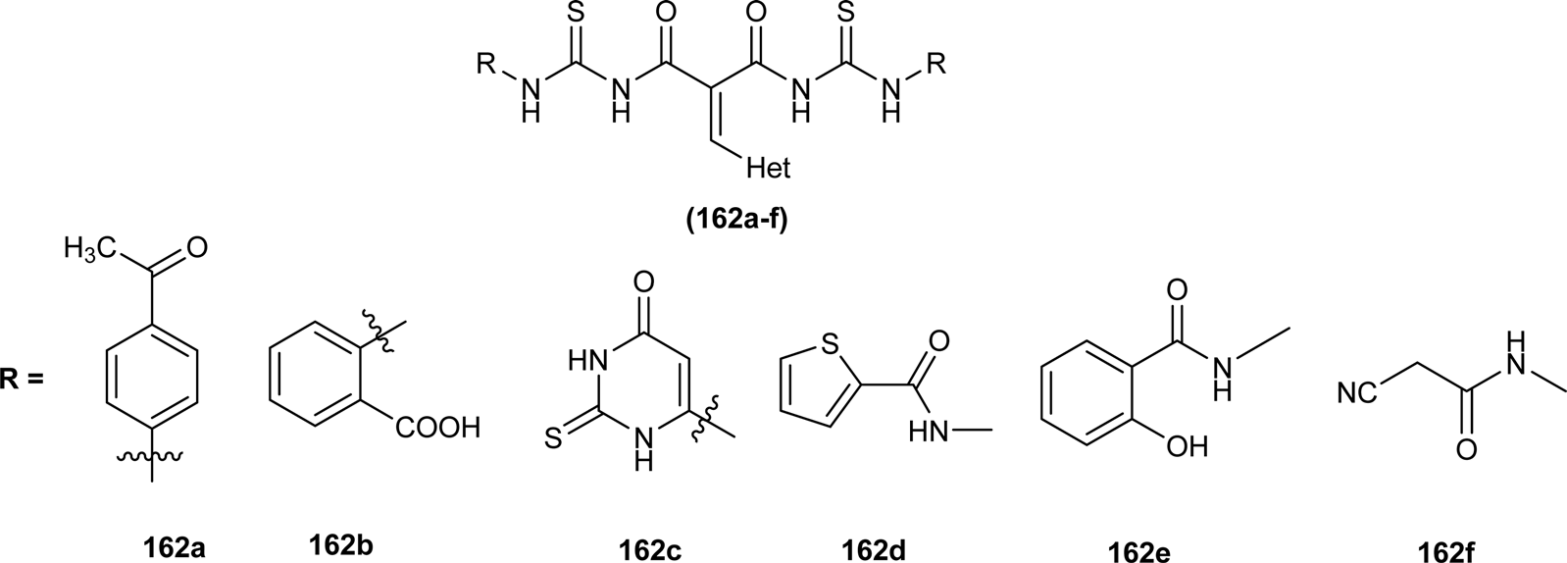
*N*-Heterocycle containing acyl thioureas.

Novel nitrogen heterocycles containing acyl thioureas (162a–f) were synthesized and evaluated for insecticidal activity against third-stage instar larvae *P. interpunctella* and *Nilaparvata lugens*. Most of the compounds exhibit weak activity, while compound (162c) showed 100% activity at conc. of 400 mg mL^−1^ against both larvae. Compound (162b) also showed prominent insecticidal activity against *N. lugens* comparable to thiamethoxam at 200 mg mL^−1^. Similarly, compound (162f) was found 56.6 and 60% active against *P. interpunctella* and *N. lugens* at 400 mg mL^−1^, respectively (Fig. 88).^140^

### 6.2 Proton conductivity

**Fig. 89:**
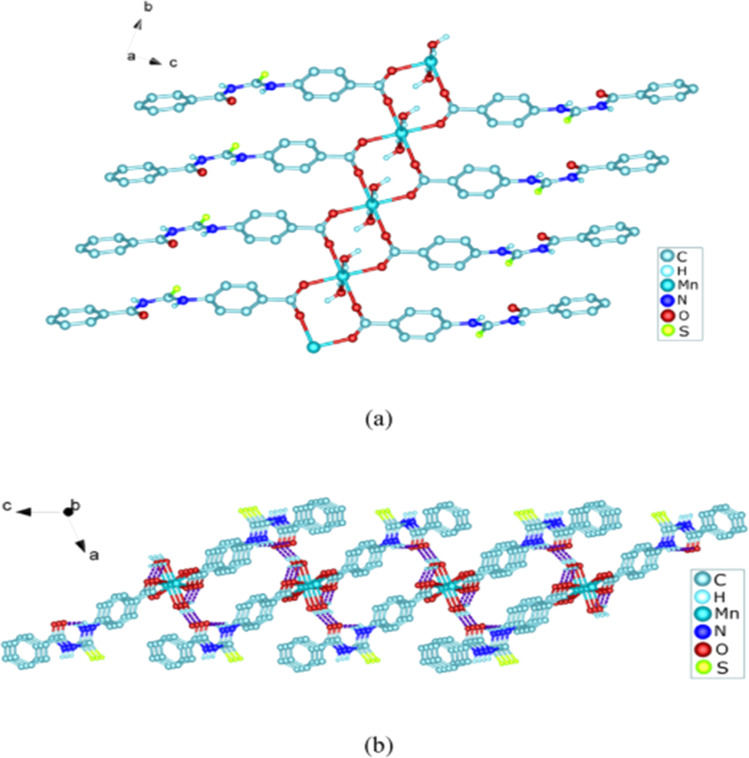
(a) Chain structure of 1; (b) 3D packing structure of 1 supported by π–π interactions and H-bonds.

Two metal–organic frameworks [Mn(H_2_BBT)_2_(H_2_O)_2_]_*n*_ (H_3_BBT = *N*-benzoyl-*N*′-(4-benzoxy)thiourea) (1), and [Cd(H_2_BBT)_2_]_*n*_ (2) were synthesized by Ye *et al.* Structures were confirmed by single crystal XRD analysis while chemical and water stability was confirmed from powder X-ray diffraction. The proton conductivity increased with the increase in humidity and temperature. With the increase of temperature, the dissociation of water molecules increased which in turn increased the proton source and led to increased proton conductivity of the MOFs. In this study, it is found that various functional groups –NH, –C=O and –C=S groups, which were not involved in coordination, formed a hydrogen bond network intermolecularly with adsorbed water molecules and this inherent hydrogen bonding was responsible for the proton conduction (Fig. 89).^141^

Zhao *et al.* synthesized {[Pr(H_2_L)_2_(NO_3_)(H_2_O)_2_]·2H_2_O·MeOH}_*n*_, from studies it was confirmed that high thermal stability and extensive intermolecular hydrogen bonds in the synthesized molecules promoted proton transfer. The proton conductivity of prepared MOF was temperature and humidity-dependent. MOF had low proton conductivity at low temperatures and independent of humidity, because of ineffective dissociation and transportation of adsorbed water molecules. The conductivity of MOF increased by increasing the temperature. Proton conductivity analysis and AC impedance test revealed that MOF had a high *σ* value 10^−4^ S cm^−1^ at 100 °C in the range of 75–98% RHs, authenticating good proton conductivity.^77^

### 6.3 Polymerizable acyl thiourea as reducing agent in self-cured composites

**Fig. 90:**
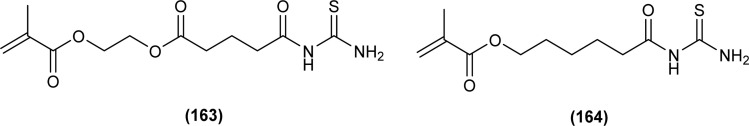
Structures of polymerizable acyl thioureas.

Two polymerizable acyl thioureas (163 and 164) synthesized by Lamparth *et al.* were evaluated for reducing properties in self-cured composites. Composites CA1-3 and CB1-10 were synthesized by adding 50 wt% of a barium–aluminum–borosilicate and 10 wt% of a spherical SiO_2_–ZrO_2_ to monomer mixtures A1-3 and B1-10. Investigation of the synthesized composites showed that the increase of copper content increased the kinetics of polymerization and DBCs at specific concentrations of the acyl thiourea (163) and CHP. Composites SCC1–3 having 50, 100 and 200 ppm of Cu(acac)_2_ achieved final double bond conversion (DBCs) after polymerization of 34.9%, 40.2%, and 47.5%, respectively. Similarly, results were also obtained by fixing the amount of copper, and gradually increasing the amount of acyl thiourea (163) and CHP. Storage of the composites at 37 °C for 45 min (dry) and at 37 °C in water for 24 h further enhanced the DBCs. For a known quantity of Cu(acac)_2_, an increase in the concentration of acyl thiourea (163) and CHP improved flexural modulus of composites. Comparing composites SCC1–3 and SCC4–6, SCC4–6 had a higher flexural modulus than SCC1–3 because of the high concentration of Cu(acac)_2_. Composites SCC5 and SCC7 were made from acyl thiourea 163 and 164, respectively had similar mechanical properties. The combinations of acyl thiourea (163 and 164), CHP and Cu(acac)_2_ were found to be highly efficient redox initiator systems. By adjusting the concentration of different constituents of composites optimal working time was successfully formulated (Fig. 90).^142^

**Fig. 91:**
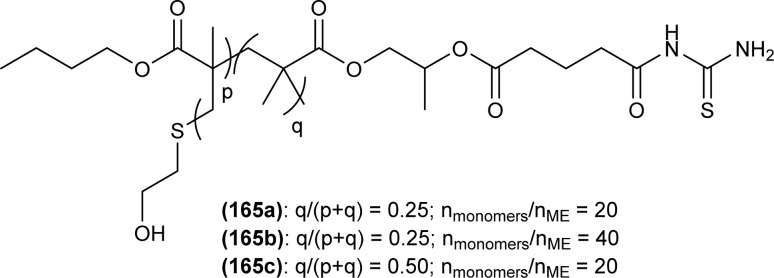
Oligomers of acyl thiourea.

Catel *et al.* synthesized acyl thiourea oligomers (165a–c) by the process of cotelomerization from butyl methacrylate and acyl thiourea methacrylate. Studies revealed that both oligomers (165a and 165b) were good reducing agents. The direct relation between the amount of Cu(acac)_2_ and flexural modulus was observed, while CHP did not affect the mechanical strength of composites. The enormous addition of oligomer (165a) slightly increased the flexural modulus. In this case, the DBC_AW_ of composites was not affected significantly by the amount of (165), CHP and Cu(acac)_2_, but a clear trend was observed in the case of DBC_AIP_. DBC_AIP_ value of composites having acyl thiourea oligomers was lower than composites having simple thiourea moiety. Increasing the concentration of Cu(acac)_2_, CHP, and acyl thiourea oligomers enhanced the polymerization kinetics. Acyl thiourea oligomer (165a, SCC1 and 2) containing composites had a higher working time than composites containing simple thiourea (ATU1, SCC8 and 9 or ATU, SCC10). It was observed that the (165a) has a lower leaching ability from cured composites than ATU (Fig. 91).^143^

### 6.4 Photoluminescence properties of complexes containing acyl thiourea

Compounds (68a–e) photoluminescence properties were studied by Tudor *et al.* in solid-state samples and dichloromethane solution form. All eight heteroleptic copper(i) complexes show strong solid-state emission compared to the solution in DCM, probably due to the rigid structure of metal complexes in the solid state that decreases the nonradiative decay pathway after excitation (Jahn–Teller distortions). Cu(i) complexes emission spectra in solid state show luminescence at *λ*_max_ 490–530 nm, under UV irradiation (340 nm). Yellow emission was observed when irradiated with *λ*_exc_ = 365 nm. The emission spectra of Cu(i) complexes are almost the same but the bulkiness of PPh_3_ and acyl thiourea ligands affect the emission spectra. For example, emission of complexes (68aa and 68ab) (509 and 516 nm) 1 : 2 (PPh_3_/67) lower in energy than complexes (68ca and 68cb) (501 and 500 nm) 2 : 1 (PPh_3_/67). The nature of halide ions also affects the emission energy of complexes mainly due to the involvement of a halide-to-ligand charge transfer (XLCT) in excited states. Thus, the emission band of derivative (68ab) red-shifted up to 7 nm having Br^−^ as compared to the chlorine containing derivative (68aa). It is important to mention that the complex (65e) has only acyl thiourea ligands and has the highest quantum yield (11%) in the solid state than other Cu(i) metal complexes of acyl thiourea (Fig. 14).^80^

### 6.5 As a precursor for spin-coated PbS thin films

**Fig. 92:**
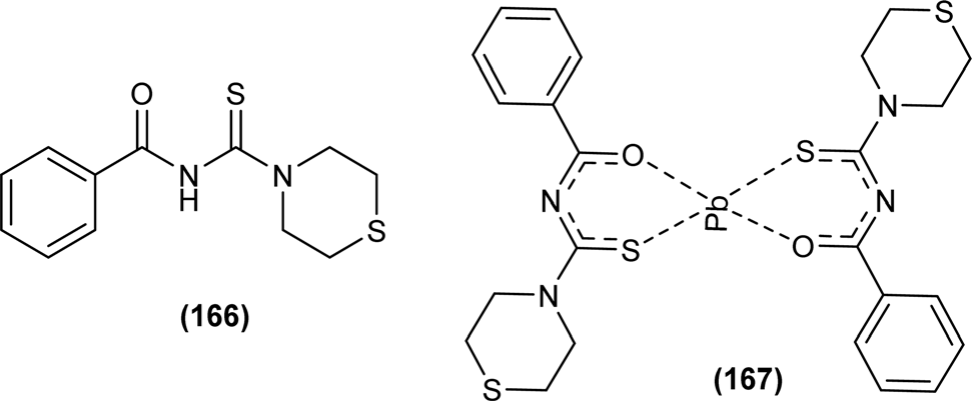
*N*-(Thiomorpholine-4-carbothioyl)benzamide and its Pb(ii) complex.

*N*-(Thiomorpholine-4-carbothioyl)benzamide (166) and its corresponding lead(ii) complex (167) were synthesized and characterized by spectroscopic techniques and single crystal XRD by Ketchemen *et al.* as a precursor for the deposition of lead sulfide thin films at ambient temperature (250 °C). Analysis study showed that crystalline PbS thin film formed in cubic phase, and compared to bulk PbS it shows blue shift in absorption maxima. By considering these properties of PbS thin films it can be used in photocatalysis. Complex (167) crystallizes in a triclinic crystal system adopting a *P*1̄ space group. Two acyl thiourea ligands coordinated with central metal atom Pb through the O and S atoms of carbonyl and thiocarbonyl (Fig. 92).^144^

### 6.6 As catalyst

Oxorhenium(v) complexes (70a-b) synthesized by Keskin *et al.* were evaluated for oxidation of Dimethylsulfide(DMS) using ^*t*^BuOOH as an oxidant. From the study, it was found that all the oxorhenium complexes show oxidation properties. Catalytic oxidative properties of the complexes having acyl thiourea ligands (propyl and butyl base ligands) cannot be distinguished because of the same electronic properties. Complex (70a) [Re^V^_2_O_2_L_2_^1^(OCH_3_)_2_] was a more active oxidizing agent than other complexes, however, Complexes (70aa, 70ba, 70ab, and 70bb) were also found efficient for the oxidation of dimethylsulfide, with good conversion rate in 1 h (Fig. 15).^81^

Acyl thiourea complexes of Ru(ii) and Ru(iii) were investigated for the reduction of nitrobenzene in the presence of NaBH_4_ as a catalyst in ethanolic solution. No reduction was observed in the presence of ligands and the absence of metal complexes. Ru(iii) complexes were found more effective in the reduction of nitrobenzene compared to Ru(ii) complexes of acyl thiourea which show less reduction activity. Complex [RuCl_2_(PPh_3_)_2_L^1^] (80a) converted 4-bromonitrobenzene into 4-bromoaniline in 30 min up to 99%, while complex [RuCl_2_(PPh_3_)_2_L^2^] did the same work in 1 h. Complexes [RuCl(CO)(PPh_3_)_2_L^1^] (80b) and [RuCl(CO)(PPh_3_)_2_L^2^] also show less catalytic activity for reduction of nitrobenzene and conversion to aniline occur up to 99% and 77% after 24 h. The lower catalytic activity of the latter two complexes was due to the presence of a strong coordinating CO ligand. Reduction of the nitro group occurs through intermediate *N*-hydroxylamine (Fig. 23).^90^

The catalytic activity of the Ru(iii) acyl thiourea complex (86) for the reduction of differently substituted nitroarenes was investigated by Uysal *et al.* Results suggest that the optimal conditions for reduction are 0.01 mol catalyst and 4 eq. NaBH_4_ in ethanol at 50 °C. Further, catalytic reduction exhibits high selectivity toward nitro group reduction without causing dehalogenation in the presence of halogen (Br, Cl) on arene moiety. But in the case of 1-fluoro-2-nitro benzene dehalogenation takes place, so the efficiency of the catalyst toward reduction for halogenated benzene is in the order F < Cl < Br depending upon the electronegativity of the halogen atom present. Similarly, when the electron-donating substituents –NH_2_ and –OH are present at the *meta* position then the efficiency of the catalyst is high but if the same substituents are present at the *para* position then the catalyst shows lower efficiency toward reduction (Fig. 27).^94^

### 6.7 Acid dissociation constant (p*K*_a_)

**Fig. 93:**
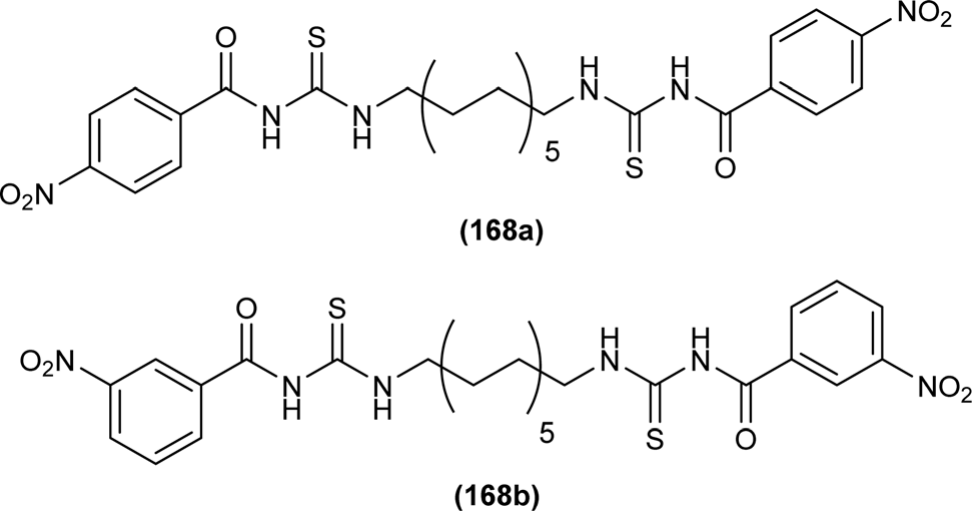
Structures of bis-acyl thiourea derivatives.

Bis-acyl thioureas (168a and 168b) were synthesized and their acid dissociation constant (p*K*_a_) was determined potentiometrically and spectrophotometrically by Efeoglu *et al.* using a hydro-organic solvent system of 50% (v/v) DMSO–water in presence of 0.1 mol L^−1^ NaCl in acidic medium at 25.0 ± 0.1 °C. using potentiometric data in HYPERQUAD software two different p*K*_a_ values for compound (168a) 7.06 ± 0.13, and 12.11 ± 0.06 were obtained, while for compound (168b) also two p*K*_a_ values 6.94 ± 0.11 and 11.17 ± 0.06 were determined. By using spectrophotometric data in HypSpec three different p*K*_a_ values for both compound (168a) 3.56 ± 0.08, 7.11 ± 0.08, and 12.30 ± 0.08 and compound (168b) 3.87 ± 0.01, 7.05 ± 0.01, and 11.82 ± 0.02 were obtained. Potentiometrically determined p*K*_a2_ values 7.06 ± 0.13 and 6.94 ± 0.11 and spectrophotometrically determined p*K*_a2_ values 7.11 ± 0.08 and 7.05 ± 0.01 for compounds (168a and 168b) belong to enthiol group in the molecule. Potentiometrically determined p*K*_a3_ values 12.11 ± 0.06 and 11.17 ± 0.06 and spectrophotometrically determined p*K*_a3_ values 12.30 ± 0.08 and 11.82 ± 0.02 for compound (168a) and (168b) relate to NH group in structure. Spectrophotometrically determined p*K*_a1_ values 3.56 ± 0.08 and 3.87 ± 0.01 for compounds (168a and 168b) may be due to enol group in acyl thiourea structure. The presence of electron-withdrawing NO_2_ group at the *para* position increases the acidity of the enol group and thus p*K*_a1_ decreases while that of p*K*_a2_ and p*K*_a3_ values increases slightly (Fig. 93).^145^

Acid dissociation constant values of the seven novel 1,4-naphthoquinone acyl thiourea hybrids (135a–g) were determined by using a computer-controlled automated titrator. After performing titration three p*K*_a_ values were found for three protonated sites LH, LH2, and LH3. By using data from titration and the HYPERQUAD computer program, the p*K*_a_ values for different protonated sites LH, LH2, and LH3 were p*K*_a1_ 3.77 ± 0.03–6.08 ± 0.04, p*K*_a2_ 8.93 ± 0.01–10.58 ± 0.01, and p*K*_a3_ 10.45 ± 0.03–11.10 ± 0.03, respectively. These p*K*_a_ values were related to NH_2_, NH and protonated carbonyl in naphthoquinone moiety (Fig. 62).^22^

### 6.8 As extractant

**Fig. 94:**
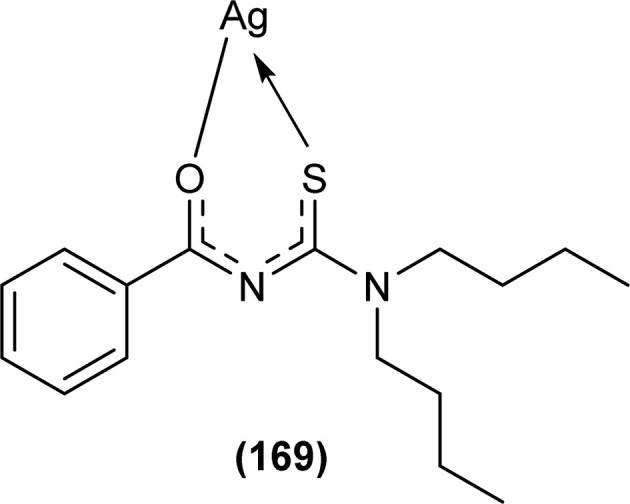
Acyl thiourea-Ag complex.

Jin *et al.* developed an ion-imprinted membrane (IIM) and utilized it as an adsorbent for the removal of Ag^+^ ions from an aqueous medium. As revealed by the dynamic adsorption study the synthesized membrane exhibited excellent separation and filtration performance when applied to the feed solution of lower conc. up to 12 mg L^−1^ and at high pH = 6. Co-existing metals presence in feed solution does not affect the removal of Ag^+^ efficiency of IIM, which reflects the selectivity of the membrane towards Ag^+^ ions. From kinetics study, it was found that the adsorption was monolayer chemisorption. Reusability investigation demonstrated that the adsorption capacity of IIM declined after five adsorption and desorption cycles. IIM surface chemically reacts with Ag^+^ ions through O and S of carbonyl and thiocarbonyl (169), respectively (Fig. 94).^146^

**Fig. 95:**
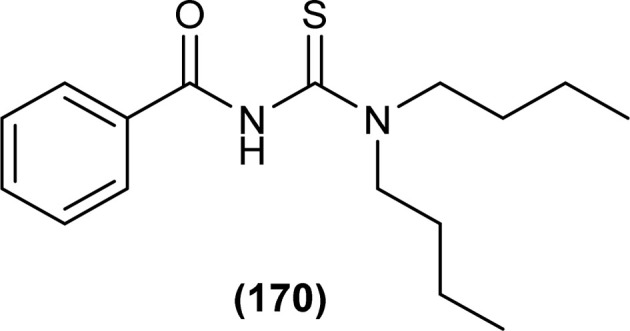
Structure of *N*-benzoyl-*N*′,*N*′-dibutyl thiourea.

Huang *et al.* synthesized polyvinylidene fluoride (PVDF)/*N*-benzoyl-*N*′,*N*′-dibutyl thiourea (170) composite membrane for Ag^+^ adsorption by mixing PVDF and (170) in DMF. Due to the addition of acyl thiourea along with the increase in active sites of the membrane, the roughness of the membrane also increased. Adsorption kinetics study of the membrane showed that the adsorption of silver ions was faster at initial 1.5 hours and slightly decreased to the value of 1.45 mmol g^−1^ at 5 hours. This showed the larger availability of active sites on the membrane surface at the initial stage of adsorption. The adsorption capability of PVDF/(170) composite membrane was also investigated for the adsorption of Ag^+^ and various other metal ions (Pb^2+^, Cu^2+^, Co^2+^, Ni^2+^, Zn^2+^, Mn^2+^, Cr^3+^ and Na^+^) to evaluate the selectivity of the membrane. It was found that the adsorption capacity of Ag^+^ was 1.44 mmol g^−1^, better than other ions. The adsorption capacity of membrane for silver ions decreased from 1.45 mmol g^−1^ to 1.23 mmol g^−1^ after ten cycles suggesting that the membrane has still 85% adsorption capacity. Thus PVDF/(170) composite membrane was proposed as reusable because it retained its maximum adsorption capacity after ten cycles of adsorption and desorption. High adsorption of silver ions was due to strong interactions among carbonyl and thiocarbonyl groups of acyl thiourea present in the membrane with Ag^+^ ions in aqueous solution. Thus PVDF/(170) composite membrane was found to be an efficient adsorbent for the removal of Ag^+^ ions from solutions (Fig. 95).^147^

**Fig. 96:**
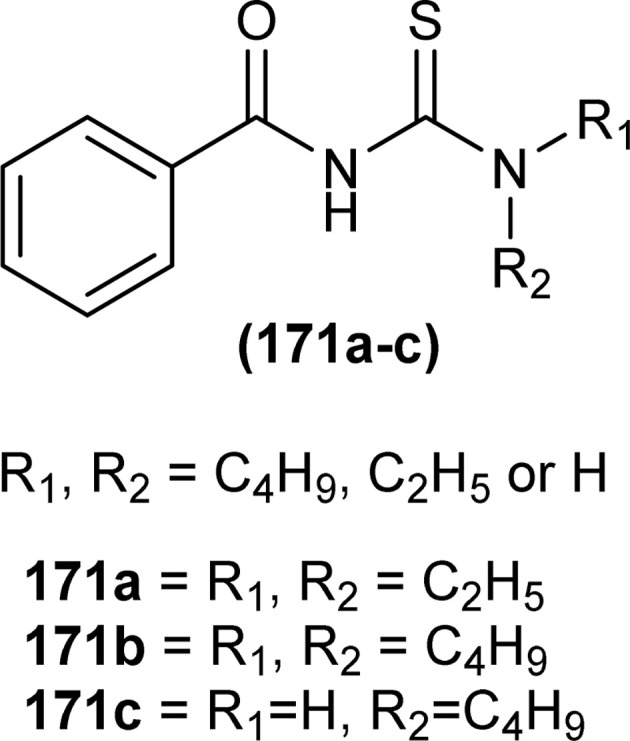
Acyl thioureas 171a–c, as an extractant for Cu(ii) ions removal.

Three novel acyl thioureas (171a–c) were synthesized and investigated as extractants for the removal of Cu(ii) ions from water. Experimental results declared compound (171a) as best extractant as compared to the two other acyl thioureas with a maximum extraction efficiency of 99.4%. Compound (171a) was found highly stable and reusable and its extraction ability lost about 5% after 8 consecutive extractions and the compound was also found highly selective for Cu(ii) ion (98%) in aqueous solution containing some other metal ions like Co(ii), Ni(ii), Zn(ii), Mn(ii), Cr(iii) and Na^+^, with extraction percentages for different ions by (171a) are 5.60%, 5.58%, 3.63%, 2.45%, 1.66% and 1.53%, respectively (Fig. 96).^148^

**Fig. 97:**
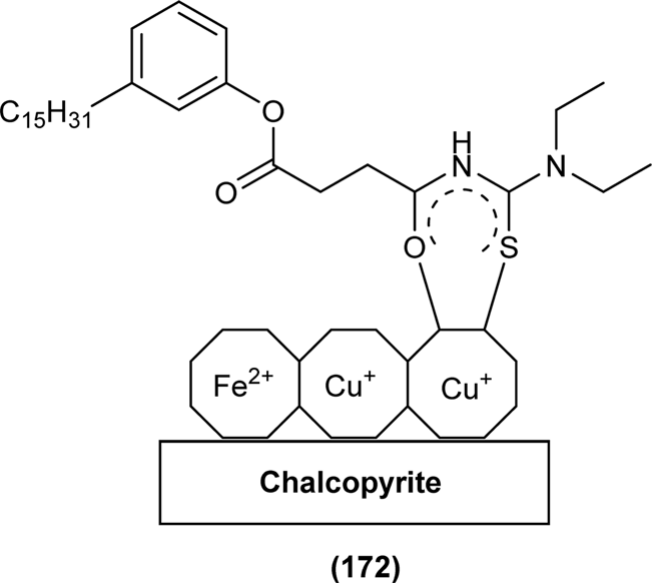
Chemisorption model of 3-pentadecylphenyl 4-(3,3-diethylthiouredo-4-oxobutanoate) on the surface of chalcopyrite.

Perera *et al.* synthesized and studied the flotation efficiency and surface absorption mechanism of 3-pentadecylphenyl 4-(3,3-diethylthiouredo-4-oxobutanoate) (172) on chalcopyrite and pyrite to separate selectively chalcopyrite from pyrite. Flotation experiment showed that collector (172) exhibits a stronger affinity for chalcopyrite as compared to its affinity toward pyrite and its performance as a collector was found higher than conventional collector potassium amyl xanthate (PAX). The enhanced selectivity and efficiency of (172) were due to chemisorption on the surface of chalcopyrite which increases its hydrophobicity. FT-IR findings demonstrated that (172) chemically react with chalcopyrite surface through O and S of carbonyl and thiocarbonyl, forming a compound with chemical structures such as C–O–Cu and C–S–Cu (Fig. 97).^149^

**Fig. 98:**
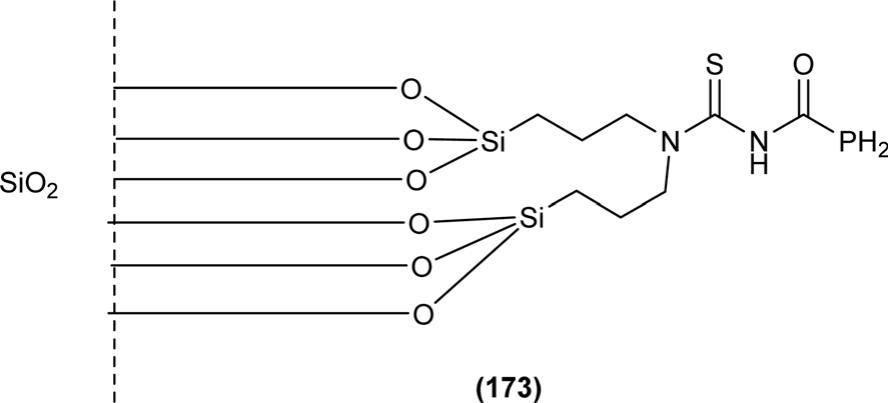
Silica-anchored acyl thiourea adsorbent.

*N*,*N*-Di(trimethoxysilylpropyl)-*N*′-benzoylthiourea was synthesized and then immobilized on the surface of silica gel to prepare novel silica-anchored acyl thiourea adsorbent (Fig. 98). From the study it was concluded that the (173) adsorbent recovered 95% of Pd and Pt from an aqueous solution at pH 2, so this potential makes the synthesized adsorbent a better-extracting agent for Pd and Pt from mining wastewater.^150^

### 6.9 Electrochemical study

MEP study of two novel acyl thiourea compounds (45 and 46) show that the negative potential of molecule localized on N of nitrile group, S and O of thiocarbonyl and carbonyl group, while positive potential localized on hydrogen atoms (N_1_–H_1A_, N_2_–H_2A_) of the urea moiety and hydrogen atoms of phenyl group. The presence of the nitro group affects the electron density of molecule and thus decreases the electron density of the cyano and thiocarbonyl group as well as affects the electron density of nitrogen of thiourea. Electrochemical properties were investigated by cyclic voltammetry analysis to determine redox potential. It was observed that the reduction potential of the cyano group decreased and shifted toward positive values because of the presence of nitro group (Fig. 6).^69^

Ethynylated-acyl thioureas (53 and 54) were synthesized and showed thermal stability up to 210 °C. Experimental electrochemical study showed that the compounds (53 and 54) undergo irreversible redox potential processes (Fig. 9).^72^

The preparation of solid-state Pb2+-ion selective electrodes based on PVC and liquid membranes using three 1-aroyl-3,3-disubstituted thioureas as ionophores was reported by Lazo-Fraga and coworkers.^151^ The electrodes showed Nernstian behavior with good sensitivities toward Pb(ii) ions detection. The sensing membranes were studied by SEM-EDS and the results have suggested the formation of aggregates through time related to complex species Pb^2+^-aroylthioureas. The effect of several substituents on the central thioureide core, including 1-benzoyl, 1-(2-furoyl), 3,3-diethyl, and 3,3-diphenyl, on the electrode performance was analyzed.^152^

### 6.10 Metallacages

**Fig. 99:**
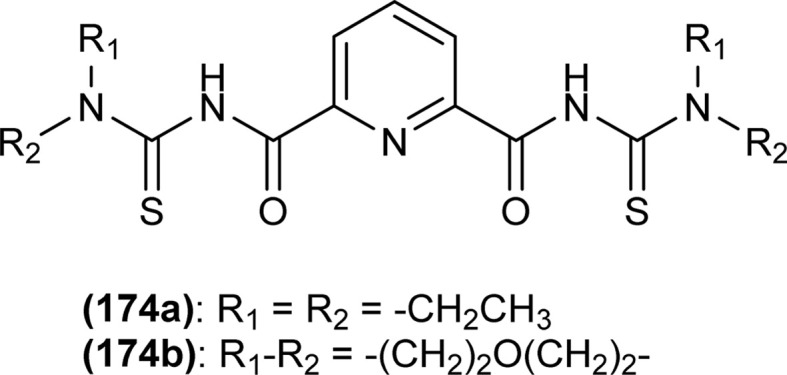
Structure of 2,6-dipicolinoylbis(*N*,*N*-dialkylthioureas).

Baitullina *et al.* synthesized 2,6-dipicolinoylbis(*N*,*N*-dialkylthioureas) (174a and 174b) based heterometallic gold metallacages as a platform for the development of nuclear medicine for different (radio)nuclides such as ^68^Ga, ^177^Lu, and ^198^Au. Synthesized metallacages were also screened for their anticancer activity against MCF-7, PC-3, U383 and U343 and it was found that the IC_50_ values of metallacages are comparable to auranofin. [Ga{Au(174a)}_2_]NO_3_ showed IC_50_ value 4.5 ± 0.7 μM against PC-3 (Fig. 99).^153^

## 7. Limitations

One of the most common way to synthesize acyl thiourea needs the employment of acid chloride in the reaction.^154^ Acid chlorides are highly unstable species; their synthesis needs extremely inert conditions and use of anhydrous solvents. The scope of solvents being used during the synthesis of acid chlorides is also limited as they need aprotic solvents like DCM, chloroform, toluene *etc.* Acid chlorides need to be dealt with extreme care as any impurity like water can convert them back to carboxylic acid. Acid chlorides also react directly with amine being used to form amides.^155^

The scope of substrates being used during the synthesis of acid chlorides is also limited *e.g.* in case 4-aminobenzoic acid the acid chloride being synthesized will react with the amino group and byproducts will be formed. Another case for byproduct occurs by the reaction of KSCN with water which forms ammonia, this subsequently reacts with acid chloride to form amide. When sufficient amount of acid chloride is not formed in the reaction, KSCN, aniline and acetone reacts to form 1 substituted 4,4,6-trimethyl-3,4-dihydropyrimidine-2(1*H*)-thione (175).^156^ These factors lower the yield and cause problems in purification therefore hamper the synthesis of acyl thiourea moiety.
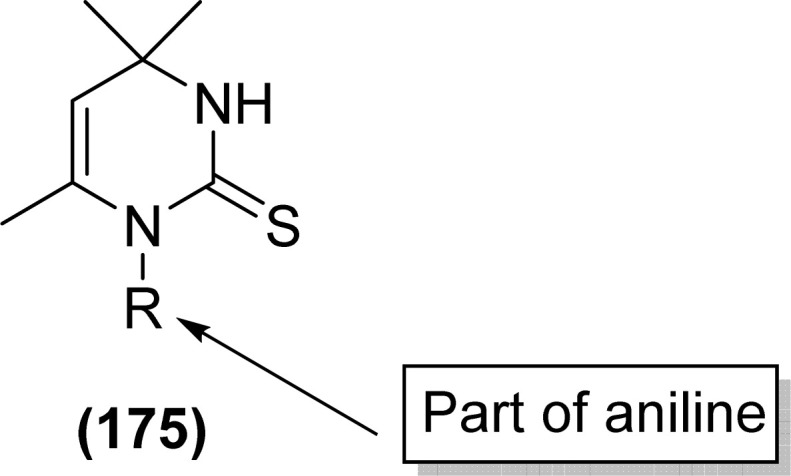


Leaching is one of the most prominent pathways to extract ores and consequently precious metals. Various ligands, such as thiourea based moieties, which can form stable complexes with these ores are used to perform this task. However, the major limitation of thiourea use is its instability in oxidative conditions during this process. Thiourea reversibly form formamidine disulfide under oxidation conditions, which further through various steps under oxidative conditions form SO_4_^2−^. This leads to high reagent consumption when using thiourea based scaffolds and therefore impedes its use in leaching technology.^157^

## 8. Conclusion

The foregoing discussion underscores the significant potential inherent in acyl thiourea derivatives. The versatility of this molecular scaffold in synthesizing biologically active compounds, metal complexes with drug-like properties, materials science applications, catalytic processes, and various other domains offers a compelling incentive for researchers to explore uncharted territories. By shedding light on these captivating aspects, this review aims to inspire further investigation into acyl thioureas, with the anticipation that ongoing research endeavors will yield novel insights and innovative solutions to address diverse challenges in the field of organic chemistry.

## Conflicts of interest

There are no conflicts to declare.

## Supplementary Material
